# Sustained formation movement and destruction of coacervates in a waste-free system driven by electricity

**DOI:** 10.1038/s41467-026-77229-1

**Published:** 2026-08-27

**Authors:** Svetlana Samokhvalova, Kalliopi Fourli, Sayed Suliman Shah, Malak Rafieq Jaber, Luis Calahorra Río, Guillermo Monreal Santiago

**Affiliations:** 1 UMR7140—Chimie de la Matière Complexe, Université de Strasbourg, CNRS, 4 Rue Blaise Pascal, Strasbourg, France; 2 IMDEA-Nanociencia, Faraday 9, Madrid, Spain

**Keywords:** Origin of life, Colloids, Self-assembly

## Abstract

Transient compartmentalization and motion are fundamental to life, and milestones in the development of complex chemical systems. Coacervates have been proposed as compartments in this context, but efforts to drive them out-of-equilibrium use exergonic reactions, which are limited by waste accumulation. Here, we present a strategy for the transient formation and movement of coacervates, using an electric potential as energy source. In a two-electrode system, the oxidation of a cysteine-containing peptide increases its multivalency, leading to complexation and coacervate formation on the anode. The electrochemically-formed coacervates move by electrophoresis to the cathode, where they dissolve by electrocatalytic reduction. The system reaches a steady state in which coacervates are simultaneously forming, moving, and dissolving in different regions of the cell. Since no waste is generated, this out-of-equilibrium regime is sustained for long times without decaying. The combination of transient formation and movement with compartmentalization opens the door for electricity-driven active transport.

## Abstract (web summary)

Coacervates are important models for protocells, but development of out-of-equilibrium systems is challenging. Here, the authors report the transient formation and movement of coacervates, with the electric potential between two electrodes used as the energy source.

## Introduction

Transient compartmentalization is a fundamental step in the development of complex systems with emergent, life-like properties^1,2^. Spatially segregating some of the components in a chemical reaction network can lead to multiple non-equilibrium regimes^3^, or to different outcomes in a community of replicators and parasites^4^. Transient compartmentalization can also be coupled to directional movement to transport chemicals against a chemical gradient. Biological examples of this are endo- and exocytosis, forms of active transport in which molecules move against a gradient by transient encapsulation into moving vesicles^5,6^.

In the cell, compartmentalization often takes place in membraneless organelles (MLOs), liquid droplets that form due to the interactions between proteins, nucleotides, and other biomolecules^7–9^. MLOs are involved in a variety of cellular processes, from RNA metabolism to protein synthesis. A common feature among some MLOs is that they are active matter: they consume energy from chemical reactions, resulting in their transient formation, movement, aging, or dissolution in response to certain stimuli, or after a period of time^10,11^.

Complex coacervates have often been used as synthetic mimics of MLOs^12–14^. Coacervates form in an aqueous solution of polymers, via spontaneous liquid-liquid phase separation (LLPS). This process is driven by attractive interactions (usually electrostatic), as well as counterion dilution^15,16^. Complex coacervate droplets have been shown to encapsulate molecules^17,18^, accelerate chemical reactions^19^, or control the aggregation of proteins^20^, similarly to their biological counterparts.

There has been a significant effort to prepare coacervates in an out-of-equilibrium regime, in order to mimic the active nature of MLOs^21,22^, or to develop synthetic protocells^23–26^. For instance, the groups of Keating^21^, Spruijt^27^, and Dhiman^28^ have shown timed and/or transient coacervate formation by reversible enzymatic phosphorylation. Walther et al. have demonstrated transient formation of DNA-based coacervates by strand hybridization and dissociation with a cross-linking nucleic acid that controls multivalency of the DNA strand^29^. Boekhoven et al. have used chemical fueling to achieve transient coacervate formation^22,30^, non-equilibrium shell formation^31^, and in a recent preprint, movement^32^. Finally, our group has recently used the decomposition of a volatile salt to trigger delayed coacervate formation and changes in coacervate properties over time^33^. Typically, the energy source driving coacervates out-of-equilibrium is an exergonic reaction, in which high-energy molecules (“fuel”) transform into low-energy “waste”. A common limitation of these systems is waste accumulation: the waste molecules accumulate in the reaction medium during reaction cycles, eventually reaching a concentration in which they have an undesirable effect on the behaviour of the system, e.g. preventing coacervate formation^22,27^. This problem is not exclusive of coacervates: practically all out-of-equilibrium systems that are driven by an exergonic reaction tend to be affected by waste accumulation, limiting for how long they can be operated^34^, or requiring a mechanism that removes the waste-products from the medium^31,33,35^.

An alternative approach to drive systems out-of-equilibrium while avoiding waste accumulation is to use an electric potential as the energy source^36^. In a recent example, Guan and coworkers^37^ have used this strategy to drive supramolecular polymerization through two redox reaction cycles. Their system is based on a thiol building block, which is soluble as a monomer but self-assembles into fibers when oxidized to a disulfide. By introducing two electrodes in the solution and applying an electric potential, the oxidation and reduction of the building block can take place at the same time but spatially separated from each other, causing assembly in the proximity of one electrode and disassembly next to the other. Since the two redox cycles only involve the transfer of electrons, and no net products are generated in the overall reaction, this system was described as “waste-free”. Importantly, the absence of waste allows the system to maintain a steady state of simultaneous self-assembly/disassembly for long periods of time (more than 4 h) with the electric potential as the only energy input.

We hypothesized that a similar strategy could be used to drive the formation of coacervates in an out-of-equilibrium regime. Two recent reports have shown that complex coacervates can be reversibly formed upon chemical oxidation or reduction^38,39^. In both cases coacervation was driven by an increase in the multivalency of one or both oligoelectrolytes. Particularly, Li et al. have shown that the oxidation of a cysteine-containing charged peptide can trigger the formation of a complex coacervate^39^, as the corresponding disulfide-linked dimer contains twice as many charges as the monomer. Even without redox-sensitive functional groups, the coacervate phase is susceptible to electric potentials due to its high charge density. Strong electric fields ( > 10 V/cm) have been shown to disrupt the internal structure of coacervates^40^, or trigger an exchange between polyelectrolytes^41^. Weaker electric fields (1–10 V/cm) do not have such an effect, but they can instead cause electrophoretic movement, which magnitude and direction depend on the surface charge of the coacervates^42–44^.

**Fig. 1:**
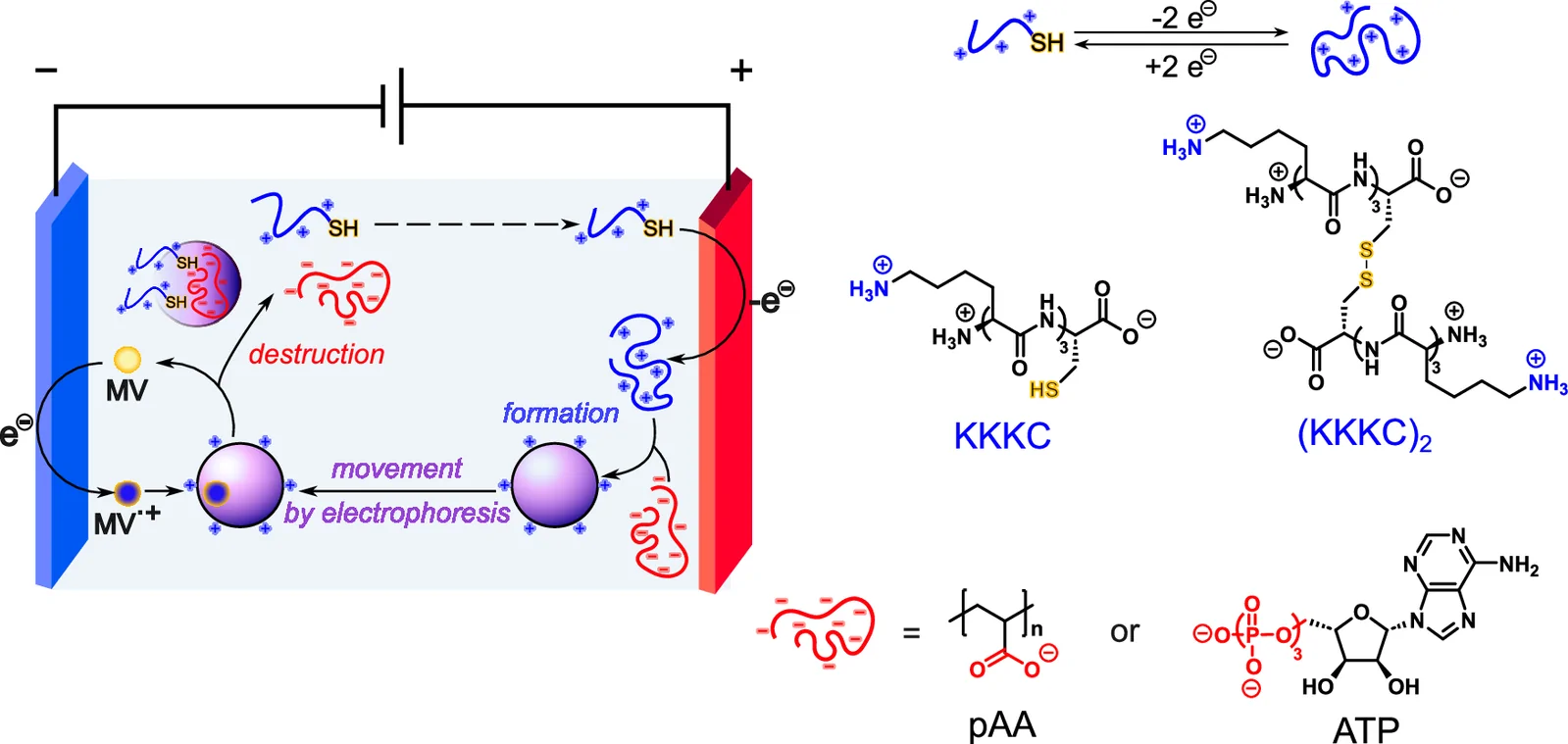
General scheme showing the electricity-driven formation, movement and destruction of (KKKC)_2_-based coacervates. The thiol group of the KKKC is oxidized at the anode to form a disulfide-bearing dimer (KKKC)_2_. The increase in the multivalency of the oligocation leads to its complexation with pAA or ATP, and to the formation of complex coacervates in the proximity of the anode. These coacervate droplets then move by electrophoresis towards the cathode. There, an electrocatalytic cycle mediated by methyl viologen (MV) leads to the reduction of (KKKC)_2_, dissolving the coacervates and recovering KKKC and the polyanion, which then diffuse back to the anode and participate again in coacervate formation.

Here, we report an out-of-equilibrium system fuelled by electricity, in which the formation, movement, and destruction of complex coacervates are driven only by the difference in electric potential between two electrodes (Fig. 1). Our system continuously produces coacervate droplets thanks to the anodic oxidation of a cysteine-containing peptide. These droplets then migrate over several millimeters to the cathode, where they are dissolved after an electrocatalyst-mediated reduction. The system is completely waste-free, and a continuous formation-movement-destruction regime can be maintained for longer than 16 h.

## Results and discussion

### Electrochemical oxidation and coacervate formation

**Fig. 2:**
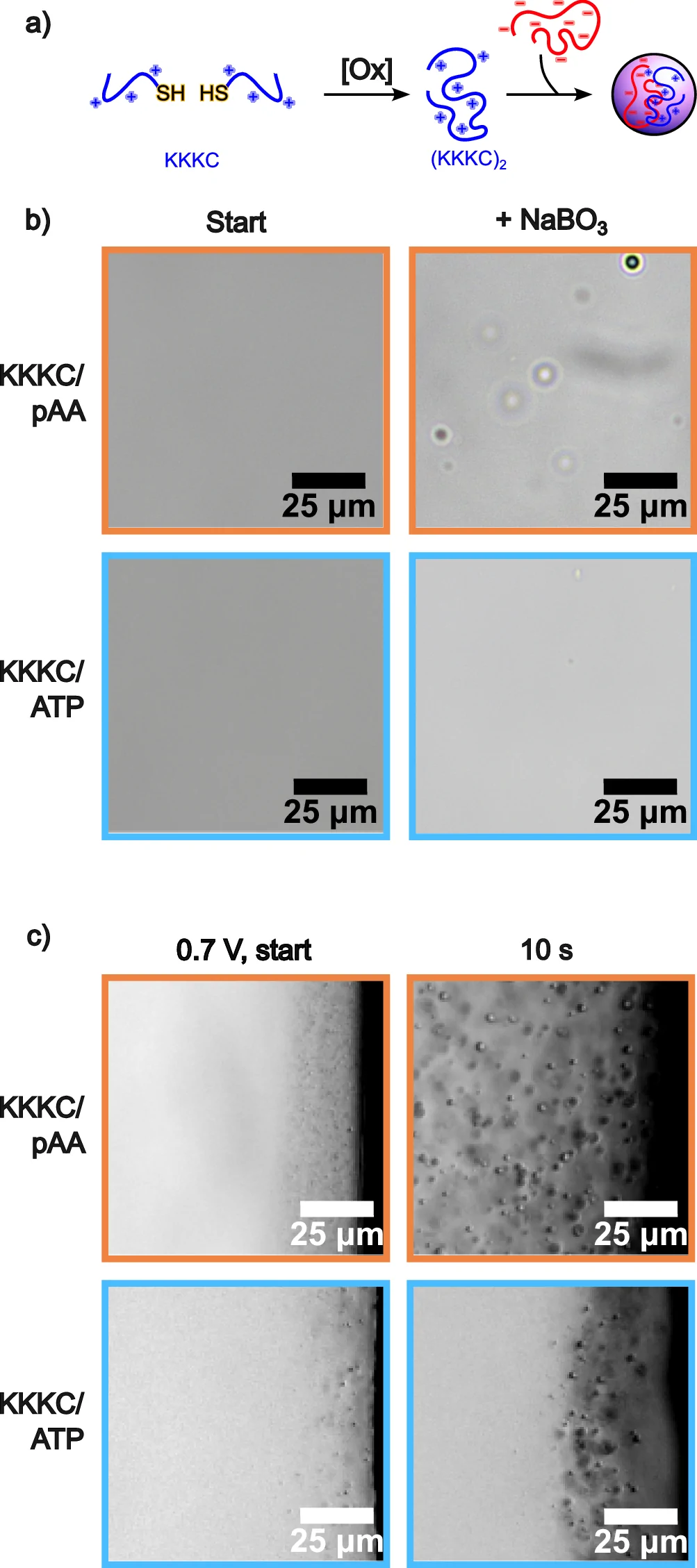
Coacervate formation triggered by the chemical or electrochemical oxidation of KKKC. **a** General scheme showing oxidation and coacervate formation in presence of a suitable oligo- or polyanion. **b** Micrographs of solutions containing KKKC and either pAA or ATP, before and after chemical oxidation (with 1.2 eq. of NaBO_3_). **c** Micrographs recorded next to the anode in a solution containing KKKC and either pAA or ATP, immediately after the application of an electric potential (0.7 V) and after 10 s. Note that the KKKC/ATP system forms coacervates when oxidized electrochemically but not after reaction with NaBO_3_. [KKKC] = [ATP] = 1.34 mM, [pAA] = 4 mM (in monomer units) [NaCl] = 100 mM in the samples with pAA, 10 mM in the samples with ATP. The size of scale bars is 25 µm.

We designed our system around a tetrapeptide (KKKC), containing three positively charged lysines and a cysteine (Fig. 1). Cysteine acts as the redox-responsive functionality: when oxidized to a disulfide, the peptide will be dimerized to (KKKC)_2_, a molecule with twice as many charges, and in principle, a higher propensity to form coacervates (Fig. 2a).

First, we explored the oxidation of KKKC and its effect on coacervate formation. Mixing KKKC with either pAA or ATP did not lead to coacervate formation in our experimental conditions. When KKKC was oxidized by the addition of NaBO_3_, the solution containing pAA became turbid, and we could observe the formation of coacervates by optical microscopy (Fig. 2b). On the other hand, the sample containing ATP did not form coacervates even after oxidation, indicating that complexation is less favorable in this case due to the lower multivalency of ATP. Oxidation with H_2_O_2_ also led to coacervate formation, while reduction with TCEP led to coacervate dissolution, confirming the redox-responsiveness of these coacervates (Fig. S1). Having confirmed that coacervation can be triggered by chemical oxidation, we studied if the same could be achieved electrochemically. For this, we prepared an undivided cell by introducing flat silver electrodes in a solution containing KKKC and pAA, and applied an external potential with a commercial power supply. The solution was not degassed, and therefore oxygen could act as a sacrificial electron acceptor as a counter-reaction to the oxydation of KKKC^45^. Note that, in the final system, the oxydation of MV is the counter-reaction (see below). As soon as the potential was applied, we observed the formation of coacervate droplets close to the surface of the anode (Fig. 2c). No droplets were observed in absence of either pAA or KKKC, confirming that these droplets were indeed the result of complex coacervation (Fig. S2). We used Ellman’s test (Fig. S3) and ^1^H-NMR spectroscopy (Fig. S4) to characterize their chemical composition, revealing that electrochemical oxidation also results in thiol oxidation and the formation of a disulfide dimer. Previous reports have shown that cysteine derivatives are difficult to oxidize on the surface of solid electrodes^46–48^. Particularly, Guan and coworkers did not observe the formation of disulfides on a glassy carbon electrode (GCE), only detecting irreversible oxidation at high potentials^49^. This was also the case for KKKC, for which we could not detect any electrochemical response by cyclic voltammetry (CV) or coacervate formation when using a GCE as a working electrode (Figs. S5 and S6). On the other hand, when the working electrode was changed to silver, we detected a broad anodic peak corresponding to the oxidation of thiols to disulfides at 160 mV (Fig. S7), consistent with our observations in the undivided cell. The reverse reaction could also be observed, as a broad reduction peak between −0.3 and −0.1 V. We ascribe the difference between the two electrodes to the high affinity of thiols for silver^50–52^.

Surprisingly, the KKKC/ATP system also formed coacervates upon electrochemical oxidation (Fig. 2c), even if it did not when oxidized with NaBO_3_. We initially hypothesized that this difference was due to the ionic by-products resulting from the chemical oxidation: the reaction between NaBO_3_ and KKKC produces borate salts, which increase the ionic strength of the solution, potentially preventing coacervate formation. However, adding an excess of different borate salts to electrochemically-formed coacervates did not result in coacervate dissolution (Fig. S8a). Increasing the pH of chemically-oxidized samples did not lead to coacervate formation either, which excludes the possibility of the pKa of ATP changing in the electric field (Fig. S8b)^53^. Preparing (KKKC)_2_ by electrochemical oxidation and mixing it with ATP once that the potential had been removed also led to coacervates, further excluding any possible effects due to the electric field (Fig. S8c). Finally, we examined the possibility that Ag^+^ ions, coming from the electrode surface, could be facilitating coacervate formation. To our surprise, adding small amounts of AgNO_3_ to (KKKC)_2_/ATP mixtures formed by chemical oxidation resulted in the formation of complex coacervates (Fig. S8d). In order to characterize this effect, we determined the salt resistance (SR) of (KKKC)_2_/ATP and (KKKC)_2_/pAA coacervates in presence and in absence of AgNO_3_ (Figs. S9 and S10). In all of the cases, the addition of Ag^+^ to the medium led to an increase in the SR of the coacervates, indicating an increase in their stability. We hypothesize that this stabilization is due to the formation of a complex between Ag^+^ and KKKC_2_, which links multiple peptides together, increasing their multivalency. The formation of complexes between Ag^+^ and disulphides has been previously described, markedly between multiple silver ions and disulphide-containing molecules^54^. Similar complexes formed between Ag^+^ and (KKKC)_2_ should be highly charged, and therefore much more prone to coacervation. Additionally, the Ag^+^ ions could act as reversible crosslinks, stabilizing the coacervate phase. The stabilizing effect of silver was more pronounced for coacervates with an excess of positive charges, confirming the binding between Ag^+^ and peptides. However, adding too large of an excess of either peptide or ATP/pAA quickly resulted in coacervate dissolution, indicating that the crosslinking due to Ag^+^ was weak compared to electrostatic repulsion. Importantly, when the same titrations were performed in presence of an excess of TCEP, no phase separation could be detected for any of the systems, even in presence of Ag^+^ and with no added NaCl. It is important to note that all the subsequent experiments in this work that involve coacervate formation and destruction were performed with KKKC:pAA and a salt concentration of 100 mM, conditions in which the electrochemistry-mediated formation of coacervates is independent of Ag^+^.

### Coacervate movement driven by electrophoresis

**Fig. 3:**
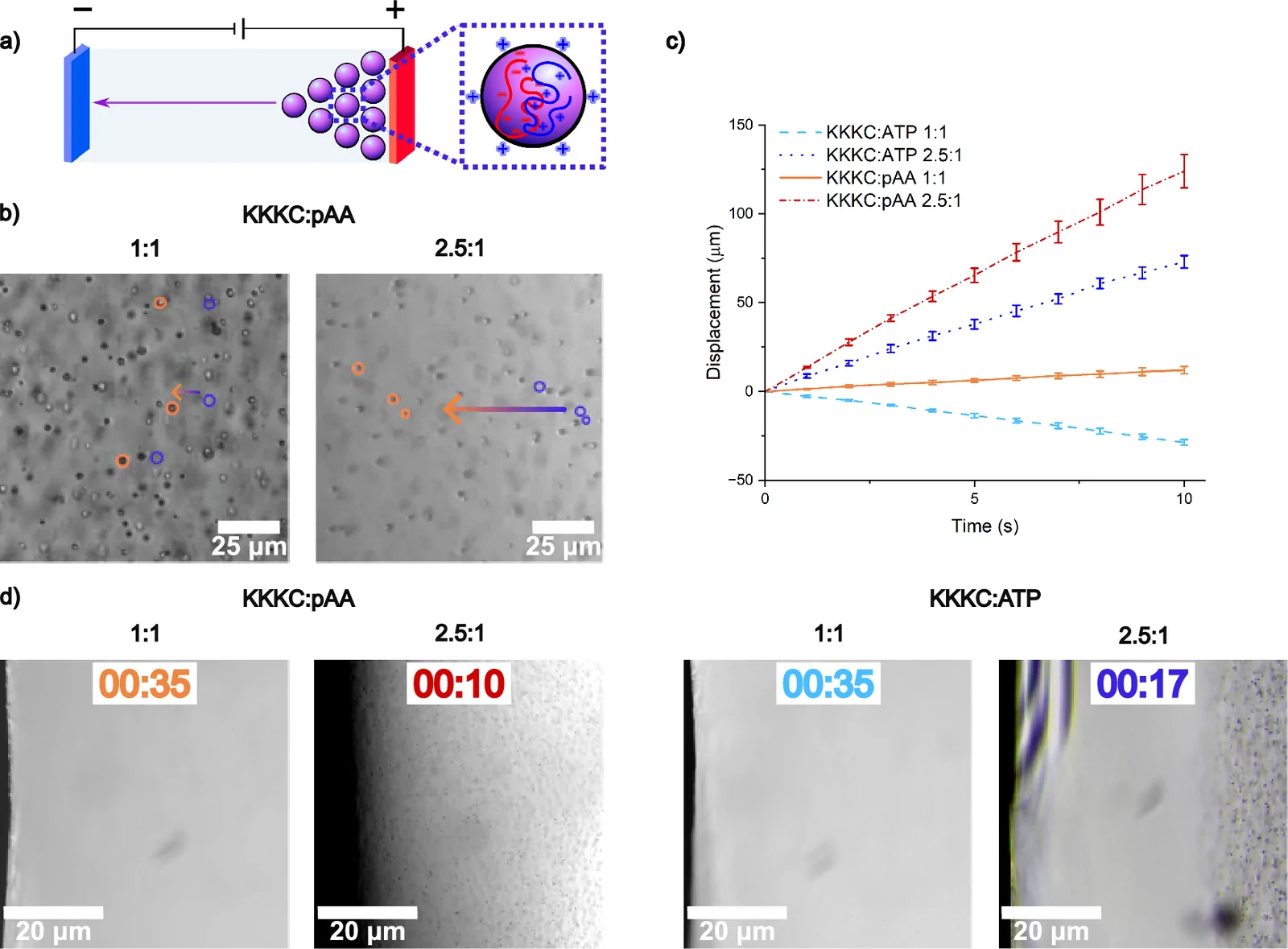
Electrophoretic movement of (KKKC)_2_-based coacervates in an electric field. **a** Scheme showing the movement of positive coacervates from the anode to the cathode. **b** Micrographs of electrochemically formed (KKKC)_2_/pAA coacervates with different ratios between positive and negative charges. The displacement of different droplets was measured over time: the blue circles represent their initial position, and the orange ones their position after 10 s. **c** Displacement over time of coacervate droplets with different compositions. Each point represents the average displacement of five droplets, and the error bars represent the standard deviation. Positive displacement values indicate movement towards the cathode. **d** Micrographs of the area next to the cathode for coacervates with different compositions, at specific times after the application of the electric potential. The strength of the electric field in all the measurements is 0.95 V/cm (voltage = 0.7 V). The initial concentrations are [ATP] = 1.34 mM, [pAA] = 4 mM (in monomer units) [KKKC] = 1.34 or 3.34 mM (for 1:1 or 2.5:1 charge ratios, respectively). [NaCl] = 100 mM in the samples with pAA, 10 mM in the samples with ATP. The size of the scale bars is indicated in the pictures. Times are reported as hh:mm.

Coacervate droplets can move in electric fields by electrophoresis^42,43^. Both the direction and the velocity of this movement depend on the ζ-potential of the droplets, and thus on the ratio between positive and negative charges in their composition. Determining the ζ-potential for coacervates cannot be done with conventional methods^42^, and requires an in-depth characterization of their viscosity, which we could not do in the current systems due to the large volumes of coacervate required. For this reason, we did not calculate the ζ-potential of these coacervates, and instead relied on electrophoretic speed as an indication of their relative surface charge. To determine the speed of (KKKC)_2_-containing coacervates and optimize their movement for a complete electrochemical cycle, we tracked the position of individual droplets over time, after the application of an electric field. (Fig. 3, Supplementary Video 1). When KKKC and pAA were mixed in a stoichiometric charge ratio (1:1) and an electric field of 0.95 V/cm was applied (0.7 V, 0.74 cm), the coacervates moved towards the cathode with a constant speed of ~1 µm/s, indicating that their overall charge was slightly positive but close to neutral. When KKKC was added in excess (2.5:1 charge ratio), their speed increased to 12.5 µm/s, and the droplets reached the cathode in ca. 10 min. This confirmed that the movement of the coacervates was mainly driven by electrophoresis, and that the non-stoichiometric mixing ratio between precursors was translated to a charge imbalance in the composition of the coacervates. For the systems containing ATP as the oligoanion, a 1:1 mixing ratio resulted in negatively-charged droplets (Fig. S11), consistent with other ATP-containing coacervates^42^. These droplets initially moved away from the anode due to droplet-droplet repulsion, but after an initial displacement they started moving back towards it. This resulted in a fixed front of coacervates that remained ~30 µm away from the electrode surface. Increasing the amount of KKKC in this system resulted in positively-charged coacervates that moved towards the cathode, but still at a slower speed than in the system containing pAA. Notably, all of the combinations resulted in spherical coacervates of approximately the same size, except for the KKKC/ATP system in a 1:1 ratio, which produced smaller droplets (Fig. S12)

These results show that there is a direct relationship between the composition of coacervates and their movement speed. Therefore, the mixing ratio between polycation and polyanion can be used to control the lifetime of the droplets: since coacervate destruction only takes place in the proximity of the cathode (see below), slower coacervates will have a longer lifetime and vice-versa.

### Coacervate destruction

Keeping a constant electric potential for longer than 10 min led to a continuous flow of coacervate droplets reaching the cathode. Even though we could not monitor the behavior of these droplets on the surface of the electrode (due to the latter blocking the light in our setup), we could detect their coalescence by the formation of a continuous coacervate phase in the bottom of the cell (Fig. S13). While some droplets sink to the bottom of the cell through its entire length, the coalesced phase was distinctly propagating from the cathode, indicating that coacervates were accumulating in that area instead of reacting. In other words, the rate at which coacervates were dissolved by the electrochemical reduction of (KKKC)_2_ was significantly slower than the rate at which they formed and reached the cathode. We hypothesize that this accumulation is caused by a difference in reaction rates between oxidation and reduction: while KKKC is soluble and its thiol group is easily accessible, most of the (KKKC)_2_ that reaches the cathode is in the form of coacervates, which potentially shield the disulfides from reaching the surface of the electrode. We confirmed this hypothesis by CV (Fig. S14): while the peak corresponding to the oxidation of KKKC was practically identical in presence or in absence of pAA, the current corresponding to disulfide reduction was significantly smaller when pAA was added. This indicates that a significant fraction of disulfides is unavailable for reaction after the coacervates are formed.

**Fig. 4:**
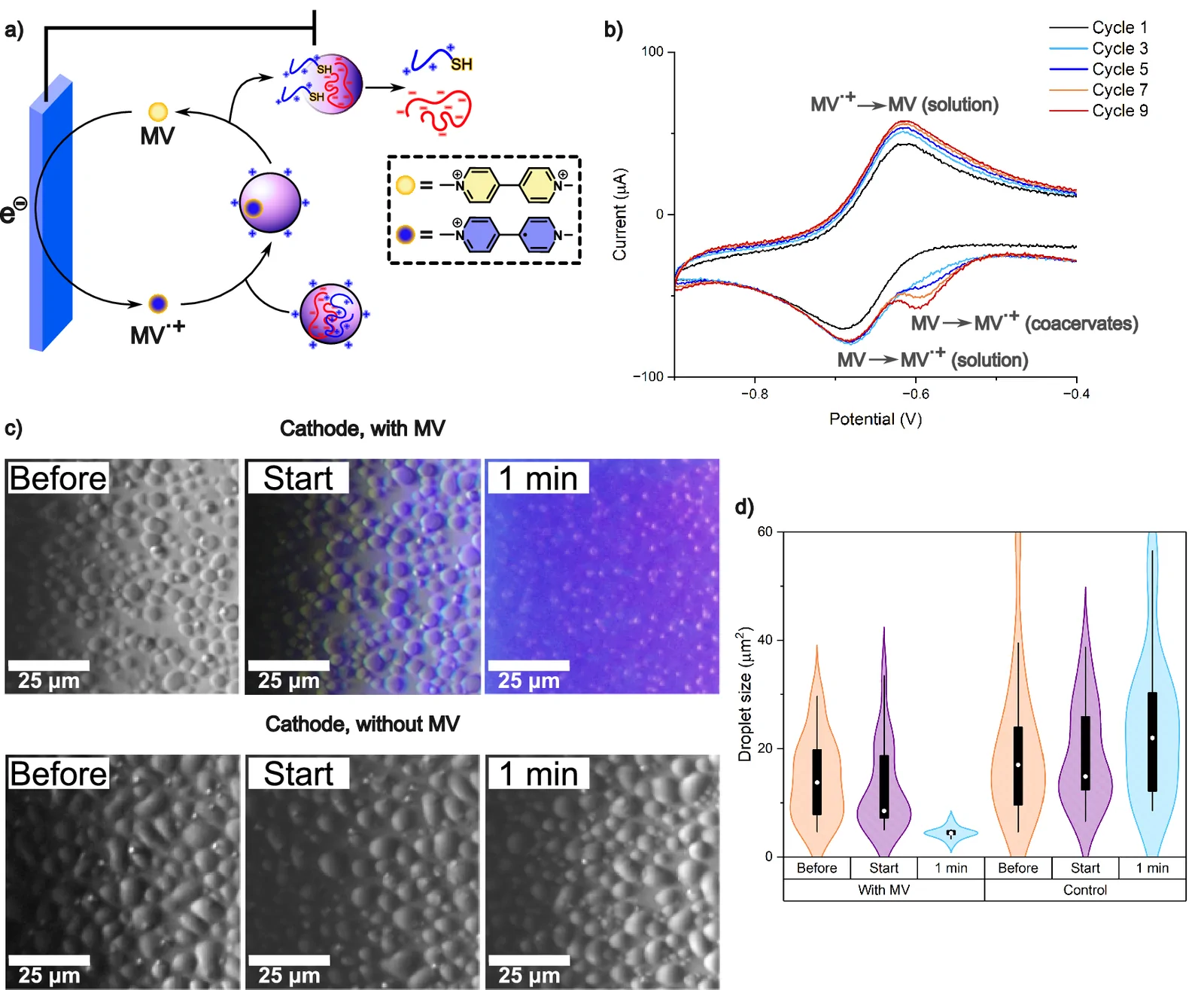
Destruction of (KKKC)_2_/pAA coacervates using an electrocatalytic cycle. **a** Scheme of the electrocatalytic cycle that results in the reduction of (KKKC)_2_, with MV as an electrocatalyst. MV^·+^ is first generated in the surface of the cathode by electrochemical reduction of MV. Then, it diffuses inside of a coacervate droplet, where it can react with (KKKC)_2_. This reaction regenerates MV and KKKC, and causes the dissolution of the coacervate. **b** CV scans (Ag working electrode, vs Ag/AgCl) for a mixture containing (KKKC)_2_/pAA coacervates and MV. Two cathodic peaks are observed for MV: −682 mV and −594 mV, corresponding to the reduction of MV in solution and in the coacervate phase, respectively. In contrast to the peak at −682 mV, the cathodic current of the peak at −594 mV increases with the number of cycles, due to the gradual accumulation of coacervates on the surface of the electrode. [pAA] = 2 mM (in monomer units), [KKKC] = 1.67 mM, [NaCl] = 100 mM, [MV] = 3.34 mM. **c** Dissolution of coacervate droplets by reaction with MV^·+^. The formation of MV^·+^ (with a characteristic blue color) can be observed immediately after the application of a 1.5 V potential. After 1 min, the average coacervate size decreases only in the sample containing MV (**d**), confirming the MV^·+^-mediated mechanism. [pAA] = 4 mM (in monomer units), [KKKC] = 3.34 mM, [NaCl] = 100 mM, [MV] = 6.68 mM. The size of the scale bars is 25 µm. The violin plot in panel d) shows the median (white dot), Q1-Q3 (black box) and Q0-Q4 (black line) for the area (in the micrograph) of 20 coacervate droplets per sample.

In order to facilitate the reduction of (KKKC)_2_ and the dissolution of coacervates, we used methyl viologen (MV) as an electrocatalyst. Methyl viologen is an organic divalent cation that can be reduced to the radical cation MV^·+^, which in turn reacts with disulfides, reducing them chemically^37^. Since MV^·+^ is small and relatively stable, it can diffuse in and out of the coacervates, reacting with (KKKC)_2_ and regenerating MV and KKKC (Fig. 4a). We tested this reaction by CV (Figs. 4b, S15 and S16). In presence of coacervates, a new cathodic peak appeared for MV at −594 mV, a slightly lower potential than its reduction in water (−682 mV). We attribute this peak to the reduction of the MV molecules that are compartmentalized into the coacervates. Since the coacervate phase is relatively hydrophobic, the coacervate droplets can sequester MV from the solution, acting as a new solvent and changing the ΔG of the reduction^19^. The intensity of the peak increases with each cycle of oxidation and reduction, consistent with the formation of more coacervates on the surface of the electrode. While the reduction of free MV in water is reversible, the cathodic peak at −594 mV appears to be irreversible. This is consistent with a chemical reaction consuming MV^·+^ as soon as it is formed^37^ – in this case, the reduction of (KKKC)_2_.

Next, we confirmed that the MV-mediated reduction of (KKKC)_2_ led to the dissolution of coacervates. For that, we prepared a sample containing KKKC, pAA, and MV, and generated coacervates by using a constant electric potential of 0.7 V. This potential already generates enough MV^·+^ to reduce (KKKC)_2_ and enable the recirculation of material (as shown below), but to clearly visualize the effect, we waited until some coacervate droplets had settled on the bottom of the cell next to the cathode and applied a higher potential (1.5 V). As soon as this potential was applied, we observed a blue color next to the cathode, characteristic of the MV^·+^ radical cation (Fig. 4c). The intensity of the color was significantly stronger within the coacervates than in the surrounding solution, indicating that the diffusion of MV^·+^ into the coacervate phase is a favorable process. Furthermore, the size of the coacervates decreased quickly after the application of the potential, confirming the MV-mediated reduction leads to the dissolution of the coacervates (Fig. 4d). Finally, we studied the chemical reduction and oxidation of (KKKC)_2_/pAA coacervates in presence of MV (Fig. S17), showing that the compartmentalization of MV in the coacervates does not affect their redox-responsiveness.

### Steady state

**Fig. 5:**
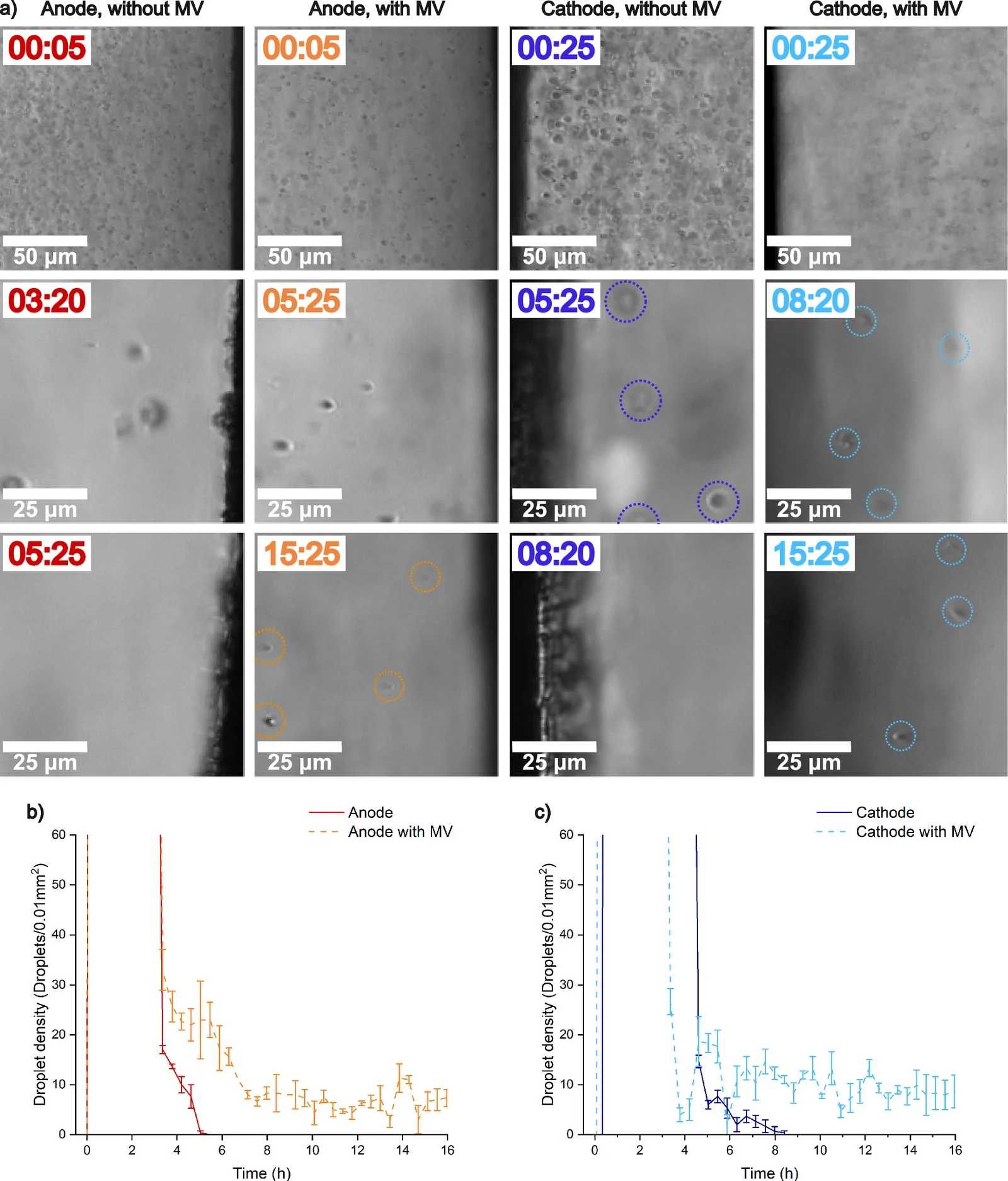
Emergence of a steady state with simultaneous coacervate formation, movement, and destruction. **a** Micrographs of (KKKC)_2_/pAA coacervates next to the anode and cathode, in presence and in absence of MV, at different times after application of the electric potential (0.7 V). The average number of droplets in a 100 ×100 µm area in the proximity of either anode or cathode was counted and plotted against time in (**b** and **c**). In presence of MV, both the generation of droplets on the anode and the supply of droplets to the cathode can be sustained for several hours, indicating that the system reaches a steady state of formation-movement-destruction. The number of droplets were obtained from manually counting the coacervates droplets in an area of 0.01 mm^2^. The data represented corresponds to the average number of three different areas at the same distance from the electrode, and the error bars represent the standard deviation. The number of droplets between 0 and 4 h (*n* > 60) was too high to count manually, but an estimation based on the grey-value of the image can be seen in Fig. S19. The size of the scale bar is indicated in the images. Times are reported as hh:mm.

When the coacervates are dissolved, free KKKC and pAA can return to the anode (by diffusion or by convective flows generated by the movement of coacervates) and participate in oxidation and coacervate formation again. In this way, the system can be driven into a sustained formation-movement-destruction regime. We characterized this regime in two systems containing KKKC and pAA. Only one of the systems contained MV, enabling the regeneration and recirculation of coacervate precursors, while the other one was taken as a control with only the “forward” part of the cycle (Figs. 5, S18, Supplementary Video 2, Supplementary Video 3). For each system, we prepared two samples and studied them for a long period of time during the application of an electric potential, in one case monitoring the proximity of the anode and in the other one monitoring the proximity of the cathode.

In an initial stage, both systems were almost identical, and we could observe the formation of coacervates next to the anode followed by their migration towards the cathode (Figs. 5a, S19). The number of droplets forming next to the anode increased for the first hour before starting to decrease as KKKC and pAA were depleted. As expected, the same trend was observed on the cathode, albeit with a short delay due to the movement along the cell. Additionally, the droplet density was slightly lower at the cathode than at the anode, as some of the coacervates sank to the bottom of the cell along the way, leading to a loss of material.

After this initial period, the number of coacervates continued to drop quickly in the system without MV, until no more droplets could be observed after 5 h at the anode (Fig. 5b) or 8.5 h at the cathode (Fig. 5c). We took this point as the moment in which the concentration of KKKC next to the anode was too low for oxidation, and the reaction could not continue without the recirculation of material. In the case of the system containing MV, the number of coacervates also decreased initially, but it never stopped completely. On the contrary, the system reached a steady state after 6 h, with a constant number of coacervates being produced and destroyed at the two respective electrodes. This steady state could be maintained for at least another 10 h without any significant changes, for a total of more than 16 h of simultaneous formation, movement and destruction. In order to confirm the role of MV in this cycle, we repeated the electrochemically-driven experiments in two samples without MV, waiting until the proximity of the anode was depleted of droplets in both of them (Fig. S20, Supplementary Video 4). We then added a solution of MV to one of the samples, and the same volume of water to the other. In the sample to which MV was added, we observed a quick increase in the density of droplets next to the anode, followed by a steady state similar to that reached in Fig. 5. On the other hand, no significant changes were observed in the sample where water was added, confirming that the formation of new droplets was mediated by MV and not simply an effect of mixing.

In order to quantify the regeneration of thiols by the reaction between MV and (KKKC)_2_, we prepared six coacervate samples by oxidation of KKKC in presence of pAA. We then applied an electric potential and added MV to three of them, while the other three were used as controls to which water was added. Immediately after the addition of MV, the concentration of thiols doubled, confirming the ability of MV to reduce the disulfides inside of the coacervates and regenerate KKKC (Fig. S21). As an additional characterization of the electrochemical reactions involved in this system, we performed chronoamperometric measurements, comparing the amount of total charge to the conversion of KKKC to calculate the Faradaic efficiency of the electrochemical cell (Fig. S22). In absence of MV, the Faradaic efficiency was very low (15 ± 10%), indicating multiple competing side-reactions and a low efficiency for thiol oxidation. On the other hand, when the same experiment was repeated in samples containing MV, the apparent Faradaic efficiency of the system was significantly higher for the first two min of the reaction (up to 30 ± 10%), due to a higher conversion of thiols to disulfides. It is worth remarking that, when MV is present in the system, the disulfides that are produced in the anode can react with the MV^·+^ produced in the cathode and convert back to thiols, decreasing the apparent conversion. Therefore, the reported 30 ± 10% value for the sample containing MV is an underestimation of the real Faradaic efficiency. This experiment showed that the presence of MV contributes to the sustained operation of this system in two ways: by reducing (KKKC)_2_ and replenishing the thiols in the anode, but also by providing a suitable counter-reaction to thiol oxidation and increasing the Faradaic efficiency of the cell.

To evaluate the effect of oxygen in this system, we repeated the experiment of Fig. 5 using a degassed solution and sealing the experimental setup to prevent exchange with air (Fig. S23, Supplementary Video 5). This degassed sample behaved in a similar way to those shown in Fig. 5, reaching a steady state after approximately 6 h. However, this steady state contained a significantly higher number of droplets, confirming that oxygen hampers the sustained formation of coacervates in this system. We hypothesize two different reasons for this: first, the reduction of O_2_ in the surface of the cathode can compete with the reduction of MV, preventing the regeneration of thiols. Second, oxygen can accelerate the passivation of the electrodes due to the formation of silver oxide. Nonetheless, the steady state still stopped after 18 h in the degassed experiment, a comparable time to the experiments in which oxygen was not removed. This confirms that other factors are responsible for the eventual collapse of the steady state, such as the degradation of the electrodes.

In order to characterize the changes happening in the system during the electrochemical cycle, we monitored the pH and conductivity of the reaction crude over a 21 h experiment (Fig. S24), as well as its composition using ^1^H-NMR. Our results show that the conductivity of the system does not significantly change over the course of the experiment—therefore, the build-up of ions is negligible. The pH increases by ~1.0, likely due to the reduction of O_2_ to OH^-^ as a competing reaction taking place in the cathode. It is important to note that this difference in pH is well within the range of values in which KKKC_2_ and pAA are charged, and the corresponding coacervates remain stable and redox-responsive in this window (Fig. S25). Finally, we could not observe significant changes in the ^1^H-NMR spectrum of the mixture (Fig. S26), besides the oxidation of KKKC to its disulphide and a shift of the KKKC peaks, likely due to the change in pH. Over the course of the experiment, the coacervates themselves remained as spherical droplets, with a slight increase in their size—from 5 to 10 µm in diameter over 10 h of experiment. (Fig. S27) We attribute this change in size to a drift in the solution composition in the proximity of the anode, as KKKC and pAA should not diffuse back from the cathode at the same speed.

These results show that, while the current electrochemical setup is not completely innocent, the secondary processes that take place in the electrodes (e.g. silver leaching, oxygen reduction) do not have a major impact in coacervate formation or stability. Furthermore, none of these processes are directly connected to the reaction cycle that controls coacervate formation and dissolution. Therefore, these secondary processes can be minimized by optimizing the reaction conditions (e.g. electrode composition, cell geometry), without changing the coacervate components or the overall design. This represents a fundamental difference between electrically-fuelled and chemically-fuelled systems: this system eventually stops working due to practical constraints, but not to anything intrinsic to the system.

As described above, the transient formation and movement of coacervate droplets are particularly appealing because of their ability to compartmentalize and transport solutes. In order to test if the electrochemically-formed coacervates described in this work can be used as compartments, we incubated different fluorescent dyes in presence of electrochemically-generated (KKKC)_2_/pAA and (KKKC)_2_/ATP coacervates, and studied their partition by fluorescence microscopy (Figs. S28–S31). Interestingly, the partition of the different dyes between solution and coacervate depends strongly on the chemical nature of the polyanion and the dyes, as well as the charge ratio and the salt concentration: depending on these parameters, some dyes were concentrated inside of the coacervates while others were excluded almost completely. It is worth remarking that these partition coefficients were determined after the overnight incubation of the dyes in presence of the coacervates, and therefore they represent equilibrated concentrations. In systems driven out-of-equilibrium, the gradients in concentration that are generated when the electric potential is applied might lead to further changes in the partition. The dye-containing coacervates still merged and coalesced, (Figs. S32 and S33) and the addition of TCEP resulted in their dissolution (Figs. S34 and S35)—confirming that they still were liquid and redox-responsive. Additionally, we could observe the electrochemically-driven cycle of coacervate formation, movement, and destruction in presence of one of these dyes (Brooker’s merocyanine), including a decrease in the rate of coacervate formation when MV was absent and a recovery of activity when it was added to the solution (Fig. S20). Taken together, these results open the door for using these coacervates as selective transporters, with a preference for compartmentalizing and moving some cargo molecules over others.

To summarize, here we have designed a waste-free, electricity-driven system for the transient formation of complex coacervates. By applying a potential between two electrodes in an undivided cell, we were able to drive simultaneous coacervate formation and coacervate dissolution (mediated by an electrocatalyst) in different regions of the cell. Furthermore, the electrochemically-formed coacervates move by electrophoresis in the direction of the electric field, effectively going from the point where they are formed to the point where they are destroyed. Each coacervate moves over a distance of 7.4 mm, over three orders of magnitude larger than their size; and while the lifetime of individual droplets is ~10 min, the regime of coacervate formation, movement and destruction can be maintained for more than 16 h. Sustaining this regime for such a long period of time would have been practically impossible in a chemically-driven system, as the accumulation of ionic side-products in the solution would destabilize the coacervate phase.

The system described here can be used as a starting point to build artificial systems that exhibit coacervate-based active transport. We have shown different molecules to be more soluble inside of the coacervate phase than in the surrounding supernatant, spontaneously being sequestered inside of the droplets. By combining this spontaneous compartmentalization with an electric potential, one can envision coacervate droplets that form in the anode, sequester cargo from their surroundings, and transport it to the cathode, releasing it upon reduction of the coacervate. As long as the coacervate-mediated transport is faster than the diffusion of free solute back to the starting point, this would lead to electrically-driven transport using coacervates as transient carriers, and to the formation of a chemical gradient. Our preliminary data show that this transport can be selective, and this selectivity could be further enhanced by including specific binding motifs in the sequence of the coacervate-forming peptides.

The system shown here was kept as simple as possible, only including two flat electrodes with a constant electric field between them. However, by combining multiple electrodes in different geometries, more complex systems can be designed, with multiple sources and sinks of coacervate droplets, and complex flows between them.

## Methods

### Materials

Poly(acrylic acid sodium salt) powder (average Mw ~5100 by GPC), Methyl viologen dichloride hydrate (98%, xH_2_O form, three molecules of H_2_O per molecule of MV were assumed for the calculations) and Tris hydrochloride ( > 99%, pH 7.0–9.0), Rhodamine B, Fluoresceine, Nile Red, Ethylenediaminetetraacetic acid ( > 99%) and Ellman’s reagent (5,5-dithio-bis-(2-nitrobenzoic acid), > 99%) were purchased from Sigma Aldrich. ATP (Adenosine-5’-triphoshpate disodium salt hydrate, 98%), Sodium perborate tetrahydrate (97%), Silver nitrate ( > 99%) and Sodium tetraborate decahydrate (Na_2_B_4_O_7_ ∙ 10H_2_O, >99%) were purchased from Alfa Aesar. Sodium metaborate (NaBO_2_, 98%) and Boric anhydride (B_2_O_3_, 98%) were purchased from ThermoFischer Scientific. Tris(2-carboxyethyl)phosphine hydrochloride (TCEP, >98%) was purchased from TCI. Sodium chloride ( ≥ 99.5 %) and hydrogen peroxide ( > 30%, aqueous solution) were purchased from Fisher Chemical. KKKC (trifluoroacetic acid salt, ≥ 95%) was purchased from GenScript Biotech (Rijswijk, The Netherlands). Brooker’s merocyanine (1-methyl-4-[(oxocyclohexadienylidene)ethylidene]-1,4-dihydropyridine, 95%) was purchased from abcr GmbH (Karlsruhe, Germany).

All samples were prepared using water deionized by reverse osmosis (~14 µS/cm).

Optical microscopy was performed on a Nikon Eclipse Ts2 microscope Type 120c in the bright field with 10x, 20x and 40x objectives. All microscope images and videos were analyzed using ImageJ 1.54 f.

### Preparation of KKKC/pAA and KKKC/ATP solutions

Stock solutions of NaCl (500 mM), KKKC (10 mM), pAA (20 mM in monomer concentration), ATP (10 mM) and MV (400 mM) were prepared in deionized water. To avoid oxidation of KKKC by oxygen in the air, the stock solution of KKKC was bubbled with nitrogen after every use, kept at −20 °C and frequently replaced.

The stock solutions were mixed to the desired concentration of each polyelectrolyte and used immediately after preparation. All samples contained Tris buffer (pH = 7.4, 50 mM). The composition of samples was as follows:

- 1:1 KKKC:pAA - 1.34 mM KKKC, 4 mM PAA (monomer concentration), 100 mM NaCl
- 2.5:1 KKKC:pAA - 3.34 mM KKKC, 4 mM PAA (monomer concentration), 100 mM NaCl
- 1:1 KKKC:ATP - 1.34 mM KKKC, 1.34 mM ATP (concentration of charged units), 10 mM NaCl
- 2.5:1 KKKC:ATP - 3.34 mM KKKC, 1.34 mM ATP (concentration of charged units), 10 mM NaCl

In the samples to which MV was added, its concentration was 6.68 mM.

The pH of the samples decreased upon mixing to ~6.5–7, due to the TFA in the peptide. The ratios between positive and negative charges were calculated assuming that the ATP bears three negative charges at this pH.

### Chemical oxidation of KKKC

The oxidation of KKKC in 1:1 KKKC:pAA and 1:1 KKKC:ATP was performed by the addition of 2.5 eq. of NaBO_3_ (20 mM solution) to the freshly prepared polyelectrolyte solutions, followed by incubation at room temperature for 45 min.

### Electrochemical set-up

For each experiment, a singular well on a 96 well plate with 7.4 × 7.4 mm square wells (Ibidi) was equipped with flat silver electrodes (2.5 mm width, 0.3 mm thickness, 99.9 % Ag). The electric potential was applied by a direct current Laboratory Power Supply EA-PS 3016-10 B (EA Elektro-automatik GmbH & Co. KG). Unless otherwise specified, the applied voltage was 0.7 V. No separator was used.

For the experiments with long measuring times, the well with the electrodes was carefully covered by a piece of a transparent film to prevent water evaporation.

### Electrophoretic movement

The displacement of individual droplets was measured by tracking the change of the x coordinate of the center of mass of a circular area corresponding to a coacervate droplet every second for 10 s. Typically, tracking an individual droplet for a longer period of time was not feasible, due to the droplet moving out of focus.

### Coacervate destruction using an electrocatalytic cycle

Two solutions containing 2.5:1 KKKC:pAA were prepared with and without MV (6.68 mM). In both cases, an electric potential of 0.7 V was applied to the mixture for 15 min, until enough coacervate droplets were accumulated in the bottom of the cell next to the cathode. Immediately afterwards, the potential was increased to 1.5 V and the microscope images were recorded within the first 30 s and after 1 min.

The size distribution of the droplets before and after application of the 1.5 V electric potential were obtained from measuring the area of the droplets next to the cathode. For each image, 20 droplets were measured and the dataset was used to generate the grouped violin graph shown in Fig. 4.

### Steady state experiments

Two different methods were used to estimate the number of coacervate droplets over the course of long experiments. For the “droplet density” estimation (See Fig. S19), the images were converted to grayscale, and the mean gray values in an area of 0.01 mm^2^ next to the corresponding electrode were measured. The data were normalized by the initial value before the coacervates had started forming. The relative droplet density was estimated by inversion of the relative mean gray value, setting the initial value as 0. For the direct counting, a square area of 100 × 100 μm was selected close to the electrode, and the droplets inside it were manually counted for frames separated by 25.2 min.

In both cases, the count was repeated three times in different areas at the same distance from to the electrode in the upper, middle and lower part of the image.

## Supplementary information

Supplementary Information

Description of Additional Supplementary Files

Supplementary Video 1

Supplementary Video 2

Supplementary Video 3

Supplementary Video 4

Supplementary Video 5

Transparent Peer Review file

## Data Availability

The authors declare that the data supporting the findings of this study are available within the paper and its Supplementary Information files. Should any raw data files be needed in another format, they are available from the corresponding author upon request.

## References

[1] Ruiz-Mirazo, K., Briones, C. & De La Escosura, A. Chemical roots of biological evolution: The origins of life as a process of development of autonomous functional systems. *Open Biol.* **7**, 170050 (2017). 10.1098/rsob.170050 PMC5413913 28446711

[2] Leon, L., Santiago, G. M. & Spruijt, E. Coacervation in systems chemistry. *Commun. Chem.* **7**, 275 (2024). 10.1038/s42004-024-01358-1 PMC11584801 39578544

[3] Penocchio,E., Bachir,A., Credi,A.,Astumian, R. D., Ragazzo,G. Analysis of kinetic asymmetry in a multi-cycle reaction network establishes the principles for autonomous compartmentalized molecular ratchets. *Chem*. S2451929424003814 (2024).

[4] Matsumura, S. et al. Transient compartmentalization of RNA replicators prevents extinction due to parasites. *Science* **354**, 1293–1296 (2016). 10.1126/science.aag1582 27940874

[5] Gundelfinger, E. D., Kessels, M. M. & Qualmann, B. Temporal and spatial coordination of exocytosis and endocytosis. *Nat. Rev. Mol. Cell Biol.* **4**, 127–139 (2003). 10.1038/nrm1016 12563290

[6] Wu, L.-G., Hamid, E., Shin, W. & Chiang, H.-C. Exocytosis and endocytosis: modes, functions, and coupling mechanisms. *Annu. Rev. Physiol.* **76**, 301–331 (2014). 10.1146/annurev-physiol-021113-170305 PMC4880020 24274740

[7] Banani, S. F., Lee, H. O., Hyman, A. A. & Rosen, M. K. Biomolecular condensates: organizers of cellular biochemistry. *Nat. Rev. Mol. Cell Biol.* **18**, 285–298 (2017). 10.1038/nrm.2017.7 PMC7434221 28225081

[8] Gomes, E. & Shorter, J. The molecular language of membraneless organelles. *J. Biol. Chem.* **294**, 7115–7127 (2019). 10.1074/jbc.TM118.001192 PMC6509512 30045872

[9] Antifeeva, I. A. et al. Liquid–liquid phase separation as an organizing principle of intracellular space: overview of the evolution of the cell compartmentalization concept. *Cell. Mol. Life Sci.* **79**, 251 (2022). 10.1007/s00018-022-04276-4 PMC11073196 35445278

[10] Portz, B. & Shorter, J. Biochemical timekeeping via reentrant phase transitions. *J. Mol. Biol.* **433**, 166794 (2021). 10.1016/j.jmb.2020.166794 PMC8154630 33387533

[11] Hondele, M., Heinrich, S., De Los Rios, P. & Weis, K. Membraneless organelles: phasing out of equilibrium. *Emerg. Top. Life Sci.* **4**, 343–354 (2020). 10.1042/ETLS20190190 32744309

[12] Aumiller, W. M., Pir Cakmak, F., Davis, B. W. & Keating, C. D. RNA-based coacervates as a model for membraneless organelles: formation, properties, and interfacial liposome assembly. *Langmuir* **32**, 10042–10053 (2016). 10.1021/acs.langmuir.6b02499 27599198

[13] Yewdall, N. A., André, A. A. M., Lu, T. & Spruijt, E. Coacervates as models of membraneless organelles. *Curr. Opin. Colloid Interface Sci.* **52**, 101416 (2021).

[14] Cao, S. et al. Dipeptide coacervates as artificial membraneless organelles for bioorthogonal catalysis. *Nat. Commun.* **15**, 39 (2024). 10.1038/s41467-023-44278-9 PMC10761997 38169470

[15] Rumyantsev, A. M., Jackson, N. E. & de Pablo, J. J. Polyelectrolyte complex coacervates: recent developments and new frontiers. *Annu. Rev. Condens. Matter Phys.* **12**, 155–176 (2021).

[16] Sing, C. E. & Perry, S. L. Recent progress in the science of complex coacervation. *Soft Matter* **16**, 2885–2914 (2020). 10.1039/d0sm00001a 32134099

[17] Wang, B., Akanbi, T. O., Agyei, D., Holland, B. J. & Barrow, C J. Chapter 7 - Coacervation technique as an encapsulation and delivery tool for hydrophobic biofunctional compounds. In *Role of Materials Science in Food Bioengineering* (eds Grumezescu, A. M. & Holban, A. M.) 235–261 (Academic Press, 2018).

[18] Forenzo, C. & Larsen, J. Complex coacervates as a promising vehicle for mRNA delivery: a comprehensive review of recent advances and challenges. *Mol. Pharmaceutics* **20**, 4387–4403 (2023). 10.1021/acs.molpharmaceut.3c00439 37561647

[19] Smokers,I. B. A., Visser, B. S.,Slootbeek, A. D., Huck, W. T. S.,Spruijt, E. How droplets can accelerate reactions─coacervate protocells as catalytic microcompartments. *Acc. Chem. Res*. acs.accounts.4c00114 (2024). 10.1021/acs.accounts.4c00114 PMC11256357 38968602

[20] Sanchez-Fernandez, A., Insua, I. & Montenegro, J. Supramolecular fibrillation in coacervates and other confined systems towards biomimetic function. *Commun. Chem.* **7**, 223 (2024). 10.1038/s42004-024-01308-x PMC11442845 39349583

[21] Aumiller, W. M. & Keating, C. D. Phosphorylation-mediated RNA/peptide complex coacervation as a model for intracellular liquid organelles. *Nat. Chem.* **8**, 129–137 (2016). 10.1038/nchem.2414 26791895

[22] Donau, C. et al. Active coacervate droplets as a model for membraneless organelles and protocells. *Nat. Commun.* **11**, 5167 (2020). 10.1038/s41467-020-18815-9 PMC7560875 33056997

[23] Abbas, M., Lipiński, W. P., Wang, J. & Spruijt, E. Peptide-based coacervates as biomimetic protocells. *Chem. Soc. Rev.* **50**, 3690–3705 (2021). 10.1039/d0cs00307g 33616129

[24] Nakashima, K. K., van Haren, M. H. I., André, A. A. M., Robu, I. & Spruijt, E. Active coacervate droplets are protocells that grow and resist Ostwald ripening. *Nat. Commun.* **12**, 3819 (2021). 10.1038/s41467-021-24111-x PMC8217494 34155210

[25] Fraccia, T. P. & Martin, N. Non-enzymatic oligonucleotide ligation in coacervate protocells sustains compartment-content coupling. *Nat. Commun.* **14**, 2606 (2023). 10.1038/s41467-023-38163-8 PMC10169843 37160869

[26] Seo, H. & Lee, H. Programmable enzymatic reaction network in artificial cell-like polymersomes. *Adv. Sci.* **11**, 2305760 (2024). 10.1002/advs.202305760 PMC11200095 38627986

[27] Nakashima, K. K., Baaij, J. F. & Spruijt, E. Reversible generation of coacervate droplets in an enzymatic network. *Soft Matter* **14**, 361–367 (2018). 10.1039/c7sm01897e 29199758

[28] S. Sharma, A. Belluati, M. Kumar, S. Dhiman. Enzymatic reaction network-driven polymerization-induced transient coacervation. *Angew. Chem. Int. Ed*. e202421620 (2024). 10.1002/anie.202421620 PMC11891636 39655501

[29] Liu, W., Deng, J., Song, S., Sethi, S. & Walther, A. A facile DNA coacervate platform for engineering wetting, engulfment, fusion and transient behavior. *Commun. Chem.* **7**, 1–10 (2024). 10.1038/s42004-024-01185-4 PMC11063173 38693272

[30] Donau, C. & Boekhoven, J. The chemistry of chemically fueled droplets. *Trends Chem.* **5**, 45–60 (2023).

[31] Bergmann, A. M. et al. Liquid spherical shells are a non-equilibrium steady state of active droplets. *Nat. Commun.* **14**, 6552 (2023). 10.1038/s41467-023-42344-w PMC10582082 37848445

[32] Boekhoven, J. et al. Size control and oscillations of active droplets in synthetic cells. *Nat. Commun.* **16**, 2003 (2025). 10.1038/s41467-025-57240-8 PMC11865504 40011463

[33] Yan, L. et al. Timed formation and aging of complex coacervates using a volatile salt. *Macromolecules* **58**, 3635–3642 (2025).

[34] Chen, X., Soria-Carrera, H., Zozulia, O. & Boekhoven, J. Suppressing catalyst poisoning in the carbodiimide-fueled reaction cycle. *Chem. Sci.* **14**, 12653–12660 (2023). 10.1039/d3sc04281b PMC10646924 38020366

[35] Sorrenti, A., Leira-Iglesias, J., Markvoort, A. J., De Greef, T. F. A. & Hermans, T. M. Non-equilibrium supramolecular polymerization. *Chem. Soc. Rev.* **46**, 5476–5490 (2017). 10.1039/c7cs00121e PMC5708531 28349143

[36] Ragazzon, G. et al. Non-equilibrium self-assembly and molecular movements powered by electrical energy. *Angew. Chem. Int. Ed.* **62**, e202214265 (2023). 10.1002/anie.202214265 PMC10107654 36422473

[37] Barpuzary, D., Hurst, P. J., Patterson, J. P. & Guan, Z. Waste-free fully electrically fueled dissipative self-assembly system. *J. Am. Chem. Soc.* **145**, 3727–3735 (2023). 10.1021/jacs.2c13140 36746118

[38] Wang, J., Abbas, M., Wang, J. & Spruijt, E. Selective amide bond formation in redox-active coacervate protocells. *Nat. Commun.* **14**, 8492 (2023). 10.1038/s41467-023-44284-x PMC10739716 38129391

[39] Wang, J., Abbas, M., Huang, Y., Wang, J. & Li, Y. Redox-responsive peptide-based complex coacervates as delivery vehicles with controlled release of proteinous drugs. *Commun. Chem.* **6**, 1–8 (2023). 10.1038/s42004-023-01044-8 PMC10630460 37935871

[40] Yin, Y. et al. Non-equilibrium behaviour in coacervate-based protocells under electric-field-induced excitation. *Nat. Commun.* **7**, 10658 (2016). 10.1038/ncomms10658 PMC4756681 26876162

[41] Liu, Z., Chen, J., Bai, Q., Lin, Y. & Liang, D. Coacervate formed by an ATP-binding aptamer and its dynamic behavior under nonequilibrium conditions. *Langmuir* **38**, 6425–6434 (2022). 10.1021/acs.langmuir.2c00580 35543367

[42] van Haren, M. H. I., Visser, B. S. & Spruijt, E. Probing the surface charge of condensates using microelectrophoresis. *Nat. Commun.* **15**, 3564 (2024). 10.1038/s41467-024-47885-2 PMC11053090 38670952

[43] Agrawal, A., Douglas, J. F., Tirrell, M. & Karim, A. Manipulation of coacervate droplets with an electric field. *Proc. Natl. Acad. Sci. USA* **119**, e2203483119 (2022). 10.1073/pnas.2203483119 PMC9372540 35925890

[44] Kim, Y. & Zheng, Y. Thermophilic behavior of heat-dissociative coacervate droplets. *Nano Lett.* **24**, 15964–15972 (2024). 10.1021/acs.nanolett.4c03058 PMC12327588 39573916

[45] Innocenti, M. et al. Electroactivation of microparticles of silver on glassy carbon for oxygen reduction and oxidation reactions. J Electrochem Soc **161**, D3018–D3024 (2014).

[46] Lawrence, N. S. & Davis, J. R. G. Compton. Electrochemical detection of thiols in biological media. *Talanta* **53**, 1089–1094 (2001). 10.1016/s0039-9140(00)00579-8 18968201

[47] Liu, X. et al. Differential pulse voltammetric determination of L-cysteine after cyclic voltammetry in presence of catechol with glassy carbon electrode. *Anal. Lett.* **45**, 2246–2256 (2012).

[48] Wen, Y., Pei, L. & Wei, T. Growth of Li doped bismuth oxide nanorods and its electrochemical performance for the determination of L-cysteine. *Mat. Res.* **20**, 592–600 (2017).

[49] Selmani, S. et al. Electrically fueled active supramolecular materials. *J. Am. Chem. Soc.* **144**, 7844–7851 (2022). 10.1021/jacs.2c01884 35446034

[50] Love, J. C., Estroff, L. A., Kriebel, J. K., Nuzzo, R. G. & Whitesides, G. M. Self-assembled monolayers of thiolates on metals as a form of nanotechnology. *Chem. Rev.* **105**, 1103–1170 (2005). 10.1021/cr0300789 15826011

[51] Shang, L. & Dong, S. Sensitive detection of cysteine based on fluorescent silver clusters. *Biosens. Bioelectron.* **24**, 1569–1573 (2009). 10.1016/j.bios.2008.08.006 18823770

[52] Leesutthiphonchai, W., Dungchai, W., Siangproh, W., Ngamrojnavanich, N. & Chailapakul, O. Selective determination of homocysteine levels in human plasma using a silver nanoparticle-based colorimetric assay. *Talanta* **85**, 870–876 (2011). 10.1016/j.talanta.2011.04.041 21726712

[53] Murke, S., Chen, W., Pezzotti, S. & Havenith, M. Tuning acid–base chemistry at an electrified gold/water interface. *J. Am. Chem. Soc.* **146**, 12423–12430 (2024). 10.1021/jacs.3c13633 PMC11082902 38599583

[54] Carrillo, D. Organodisulphide complexes. *Coord. Chem. Rev.* **119**, 137–169 (1992).

