# Supplementary material for: Sustained formation movement and destruction of coacervates in a waste-free system driven by electricity
Source: Nat Commun. 2026 Aug 27;17:10245. doi: 10.1038/s41467-026-77229-1 (PMC13616953; doi:10.1038/s41467-026-77229-1)
Supplement: Supplementary file 1 [file 41467_2026_77229_MOESM1_ESM.pdf]

Supplementary Information for:

Sustained Formation Movement and Destruction of Coacervates in a Waste-Free System Driven by Electricity

Svetlana Samokhvalova[1], Kalliopi Fourli[1], Sayed Suliman Shah[1], Malak Rafieq Jaber[1], Luis Calahorra Río[2], Guillermo Monreal Santiago[1]*

1- UMR7140 – Chimie de la Matière Complexe, Université de Strasbourg, CNRS, 4 Rue Blaise Pascal, 67081 Strasbourg, France

2- IMDEA-Nanociencia, Faraday 9, 28049, Madrid, Spain

*Corresponding author. Email: monrealsantiago@unistra.fr

Supplementary materials

Vitreous carbon substrates were purchased from Neyco (Vanves, France) as 25x25 mm^2 x500 µm plates and cut with a diamond disk, to obtain 500 µm-thick electrodes with a width of approximately 4 mm.

^{1}H NMR was performed using a 300 MHz Avance III HD (Bruker) spectrometer, with D_2O as a solvent. Chemical shifts were adjusted using the peak of the solvent as a reference (4.79 ppm), and different spectra were aligned based on the peak of the Tris buffer (3.75 ppm).

Supplementary methods

Chemical oxidation and reduction of KKKC

The dimer $(KKKC)_2$ was prepared by oxidation of KKKC with H_2O_2. A stock solution of KKKC (10 mM) was mixed with a 30% (w/w) solution of H_2O_2 to a final concentration of 220 mM and incubated overnight at room temperature to ensure the full oxidation of the thiol groups. This stock solution was then added to a solution containing either pAA or ATP in the desired ratio.

Alternatively, H_2O_2 (30% w/w) was added directly to a mixture of KKKC and either pAA or ATP to observe the formation of coacervate droplets (the final concentration of H_2O_2 was 29 mM). Addition of tris(2-carboxyethyl)phosphine hydrochloride (TCEP) (490 mM stock solution) directly to these coacervates led to their immediate dissolution due to the reduction of the disulfides. The final concentration of TCEP was 20 mM in the experiments with electrochemically formed coacervates and 32 mM in the experiments where coacervates were prepared by H_2O_2 addition.

Sample preparation for cyclic voltammetry (CV)

All samples were prepared using a background solution containing 50 mM of Tris buffer (pH = 7.4) and 100 mM of NaCl in deionized water. The concentration of polyelectrolytes for the CV experiments was reduced by half due to the larger sample volumes. The samples had the following concentrations: KKKC – 1.67 mM; pAA – 2 mM (in monomer units), MV – 3.34 mM. The formation of coacervates at this lower polyelectrolyte concentration was confirmed by optical microscopy. Coacervates were prepared from the mixture of 1.67 mM KKKC and 2 mM pAA in the background solution (15 ml) by oxidation (35 min, 0.9V, flat silver electrodes) in a glass vial upon stirring. CV measurements were performed directly after the oxidation to minimize the coalescence and sedimentation of the coacervates. After the analysis, MV was added to the mixture and the CV was repeated. To obtain the pAA+MV control curve, the same oxidative conditions were applied to a solution of pAA, after which MV was added to the solution directly before the CV measurement.

Cyclic voltammetry (CV)

CV was performed using a PARSTAT 2273 potentiostat with a silver electrode (OD x ID = 6.0 x 3.0 mm, A 002419, ALS) or a Glassy Carbon Electrode – GCE (OD x ID = 6.0 x 3.0 mm, A002012, ALS) as a working electrode, Pt wire as a counter electrode and a Ag/AgCl (Re-1S Ag/AgCl in 3 M NaCl – length: 50 mm – OD: 4.5 mm, A-012168, ALS) reference electrode. No separators were used. The scans were recorded from -0.9 to 0.2 V for the silver electrode and from -1 to 1 V for the GCE with a 100 mV/s scan rate. The measurements were performed in room temperature under nitrogen. Typically, 10 CV cycles were performed to increase the intensity of the anodic and cathodic peaks of KKKC.

Quantitative determination of thiols

The concentration of thiols was determined by the Ellman's assay. Aliquots of the sample to be analyzed (5.99 µL) were taken with a micropipette and diluted with a solution containing Tris buffer (50 mM, pH = 7.4) and NaCl (100 mM). 5,5-Dithio-bis-(2-nitrobenzoic acid) (DTNB) (15 mM, stabilized with 2 mM EDTA and centrifuged) was added to each sample to a final concentration of DTNB of 0.5 mM. The total volume of all samples was 100 µL. The samples were immediately transferred to a 96-well plate (Greiner Bio-One) and analyzed using VANTAstar plate-reader (BMG LABTECH, Ortenberg, Germany). Absorbance spectra (220-650 nm, 2 nm interval, 25 flashes per well, volume pathway correction) were recorded for all samples, and an aqueous solution containing Tris buffer (50 mM, pH=7.4) and NaCl (100 mM) was used as a blank.

A calibration was performed measuring the absorbance at 412 nm of solutions containing KKKC (0, 0.1, 0.2, 0.3, 0.4 mM), and DTNB (0.5 mM). Each of the solutions was prepared in triplicate. The calibration curve was fitted by linear regression (R^2=0.99) used to determine the concentration of thiol groups in the rest of the samples. Unless indicated otherwise, all the quantitative measurements of thiol concentration were performed in triplicate.

Chronoamperometry

Chronoamperometry (CA) experiments were performed using a PARSTAT 2273 potentiostat with flat silver wires as both working and counter electrodes and an Ag/AgCl (Re-1S Ag/AgCl in 3 M NaCl – length: 50 mm – OD: 4.5 mm, A-012168, ALS) reference electrode. The scans were recorded at 0.7 V for 300 s. The measurements were performed in room temperature under nitrogen with no stirring or bubbling. The samples of 1.5 mL were prepared in water with [KKKC] = 3.34 mM, [pAA] = 4 mM (in monomer units), in absence and in presence of MV ([MV] = 6.68 mM). All samples contained Tris buffer (50 mM, pH = 7.4) and NaCl (100 mM).

Determination of Faradaic efficiency

The Faradaic efficiency (FE) for the oxidation of KKKC with and without MV was determined using Equation (1):

$$\boldsymbol{FE}\ (\%) = \frac{\boldsymbol{n * F * N}}{\boldsymbol{Q}} * \mathbf{100} \qquad (1)$$

Where n is 1 (the number of electrons required for the half-reaction $KKKC \rightarrow \frac{1}{2}(KKKC)_2 + e^-$), F is the Faraday constant (96485 C/mol), N is the number of moles KKKC that have been oxidized at a given time, and Q is the total charge passed through the working electrode until that same time (in Coulombs, C).

The concentration of thiols was analyzed in aliquots (5.99 µL), taken with a micropipette immediately upon preparation and during the CA measurements at 60, 120 and 300 s. The concentration of thiols in each of the aliquots was analyzed in a plate reader by the Ellman's assay (see above). These values were used to determine the number of moles of KKKC converted into $(KKKC)_2$ *(N)*.

The total charge (Q) was calculated based on the CA measurements by integrating the current over time. The integrated values at 60, 120 and 300 s were used to calculate the FE.

It is worth noting that, for the sample containing MV, the calculated Faradaic efficiency is lower than the real one, since thiols are regenerated by the reaction between $MV^{\cdot+}$ and $(KKKC)_2$, lowering the apparent value of N. Additionally, the amount of $MV^{\cdot+}$ generated in this system will be higher than in the microscope experiments, since there is a significantly larger surface of the electrode in contact with the solution.

Electrochemical reduction of thiols in presence and absence of MV

Solutions containing [KKKC] = 3.34 mM, [pAA] = 4 mM (in monomer units) were prepared in presence and in absence of MV (6.68 mM). All final solutions (200 µL) contained Tris buffer (50 mM, (pH = 7.4)) and NaCl (100 mM), following the general protocol. H_2O_2 (3.34 µL of a 500 mM stock solution, 2.5 equiv. with respect to KKKC) was added, and the samples were incubated in an ultrasonic bath for 25 minutes at 35 °C before transfer to a microwell plate equipped with flat silver electrodes. A potential of 0.7 V was then applied to this system, followed after 5 minutes by the addition of either MV (16.5 µL of a 400 mM stock solution, 10 equiv. with respect to KKKC) or water (16.5 µL).

The concentration of thiols was monitored in both samples and controls by quantitative Ellman's assay (see above)

Steady state experiments in absence of oxygen

In order to study the steady state during a formation/destruction regime in oxygen-free conditions, a 2.5:1 KKKC:pAA solution (200 µL) was prepared following the general protocol (MV ([KKKC] = 3.34 mM, [pAA] = 4 mM (in monomer units), [MV] = 6.68 mM. [Tris buffer (pH = 7.4)] = 50 mM, [NaCl] = 100 mM)), and degassed by gently bubbling nitrogen for 2 minutes. Under N_2 flow, this solution was transferred to a 96 well plate (7.4x7.4 mm square wells, Ibidi) and sealed with an adhesive sealing tape (Thermo Scientific). Then, two incisions were made in the film, through which flat silver electrodes were pushed and placed next to the walls. Finally, the incisions were covered with a fast-drying glue to seal the setup, which was immediately connected to the power supply to start the experiment.

Ellman's test for the qualitative detection of thiols in chemically and electrochemically oxidized samples

UV-VIS analysis was performed on a PerkinElmer Lambda 650 S UV/Vis spectrometer. Spectra were recorded from 600 to 300 nm with a 1 nm interval and a 266.75 nm/min scan speed against air as a reference.

All samples contained 50 mM Tris buffer (pH = 7.4) and 100 mM NaCl.

To obtain electrochemically-oxidized coacervates, 1 ml of a typical 2:5:1 KKKC/pAA mixture was oxidized (0.9 V) while stirring for 30 min in a 2 ml microtube, equipped with two flat silver electrodes.

Chemical oxidation of a KKKC/pAA mixture was performed by adding 2 eq. of $NaBO_3$ (20 mM, freshly prepared solution) to an already diluted sample of 2:5:1 KKKC/pAA and letting it equilibrate for 1 hour.

Solutions of KKKC, KKKC/pAA, chemically oxidized $(KKKC)_2$/pAA coacervates, and coacervates freshly-prepared by electrochemical oxidation were freshly prepared as indicated. An aliquot of each sample was diluted to a final concentration of 0.2 mM of KKKC. Ellman's reagent (10 mM suspension of 5,5-dithio-bis-(2-nitrobenzoic acid), or DTNB, stabilized with 2 mM EDTA) was added to each sample to a final concentration of DTNB of 0.5 mM. A UV-Vis spectrum was recorded after a 5 min incubation. The blank solution contained 50 mM Tris buffer (pH = 7.4) and 100 mM NaCl.

Nuclear Magnetic resonance (NMR)

All stock solutions (100 mM pAA, 200 mM Tris buffer (pH = 7.4)) and samples were prepared in D_2O. All samples contained 50 mM Tris buffer and 100 mM NaCl (before oxidation). KKKC, NaCl and $NaBO_3$ were added to the samples as solids. The initial concentration of KKKC in all samples was 3.34 mM, the concentration of pAA was 4 mM (in monomer units), matching the typical 2.5:1 KKKC:pAA system. First, a KKKC control and a KKKC/pAA mixture (1 ml each) were prepared and analyzed by ^{1}H NMR. The

KKKC/pAA mixture was then transferred into a 2 ml microtube with a stirring bar, equipped with flat silver electrodes. The mixture was oxidized (0.9 V) while stirring for 90 min. The obtained coacervates were dissolved by adding solid NaCl to a final concentration of 2 M, after which the solution was analyzed by ^{1}H NMR. To prepare the oxidation controls, fresh KKKC solutions were either electrochemically oxidized in the same conditions or chemically oxidized with $NaBO_3$ (3.7 equiv., approximately 1 hour before recording the spectrum). Both controls contained 2 M of NaCl.

For the ^{1}H NMR analysis of the system before and after a 21-hour electrochemical experiment (Figure S26), a 2.5:1 KKKC:pAA sample was prepared using H_2O as a solvent, and subjected to a 0.7 V electrochemical potential following the standard protocol. Aliquots of this sample were taken before starting the experiment and after 21 h, and they were lyophilized, and redissolved in D_2O to final concentrations of [KKKC] = 3.34 mM, [pAA] = 4 mM (in monomer units), and [MV] = 6.68 mM. Solid NaCl was added to both NMR samples to a final concentration of 2 M, to ensure that the coacervates were completely dissolved for the NMR analysis.

Conductivity and pH measurements

Conductivity and pH were measured using a Mettler Toledo SevenExcellence™ instrument with a conductivity probe electrode InLab® 751-4MM Mettler Toledo (1 point calibration) and pH meter InLab® Micro Pro-ISM (3-points calibration). Samples containing 2.5:1 KKKC:pAA and MV were prepared according to the standard protocol but in a larger volume (1.5 mL). The concentration of KKKC in the samples was 3.34 mM, the concentration of pAA was 4 mM (in monomer units), and the concentration of MV was 6.68 mM. All samples contained Tris buffer (50 mM) and NaCl (100 mM). The samples were prepared in 3 mL glass vials outfitted with silver electrodes and a magnetic bar. An electric potential (0.7 V) was applied to the system, and the conductivity and pH were measured continuously in independent samples. For the conductivity measurements, the sample was kept at 25 °C using a sand bath to prevent drifting due to the temperature fluctuations.

Determination of salt resistance

The salt resistance (SR) of the coacervate samples was determined by turbidimetry (optical density at λ = 600 nm) and optical microscopy. Turbidity measurements were performed either on a PerkinElmer Lambda 650 S UV/Vis spectrometer, using air as a reference, or in a a VANTAstar plate-reader (BMG LABTECH, Ortenberg, Germany), using water as a blank.

Samples were prepared by either electrochemical oxidation with a 0.7 V potential (30 minutes for PAA-containing samples and 100 minutes for ATP-containing samples) or with $(KKKC)_2$ prepared via chemical oxidation overnight. For the experiments in presence of Ag^+ ions, a 5 mM stock solution of $AgNO_3$ was added to mixtures of $(KKKC)_2$ and ATP/pAA to a final concentration of 0.134 mM. All samples were prepared using Tris buffer (50 mM) and initially contained no added salt. The samples were then titrated with a NaCl stock solution (4 M) and monitored by turbidity and optical microscopy. When a ratio between two concentrations is reported, it is always in reference to a concentration of 4 mM in charge units. (e.g. 1:5 KKKC:pAA corresponds to 4 mM of Lysine residues and 20 mM of acrylate groups).

All titrations were repeated in reducing conditions for samples containing KKKC and either ATP or pAA, both in presence and in absence of Ag^+. TCEP (2.5 equiv. compared to KKKC) was added to these samples to ensure the complete reduction of the peptide. No coacervates could be found in these conditions, even without any added salt.

Regenerating the droplets in a steady-state system

Samples containing 2.5:1 KKKC:pAA and MV were prepared according to the standard protocol ([KKKC] = 3.34 mM, [pAA] = 4 mM in monomer units, final volume = 200 µl). An electric potential (0.7 V) was then applied until no droplets were observed in the close proximity of the anode (< 5 droplets / 0.01 mm^2). Then, 10 eq. of MV (16.7 µl of a 400 mM stock solution) were carefully added to the cell while keeping the electric potential, and a timelapse was recorded. The density of the droplets was calculated in ImageJ as the average number of droplets in three different areas of 100 x 100 µm in the proximity of the anode. A control experiment was performed following the same protocol, but adding 16.7 µl of water instead of the MV stock. Both experiments were performed in the presence of Brooker's merocyanine as a model cargo molecule (0.95 µM). Both samples contained Tris buffer (50 mM) and NaCl (100 mM).

Morphological study

The size and circularity of coacervate droplets were determined with ImageJ. The analyzed videos were Supplementary Videos 1 (coacervate movement) and 2 (anode with MV, Figure 5). The values reported correspond to 25 droplets for each coacervate composition.

Fluorescence microscopy

Fluorescent microscopy was performed on a Nikon Eclipse Ts2 microscope Type 120c in fluorescent mode.

Stock solutions of all dyes were prepared in water to a 20 µM concentration. To increase the solubility of fluorescein, NaOH (20 µM) was added to the stock solution. Polyelectrolyte mixtures 1:1 KKKC:pAA, 2.5:1 KKKC:pAA, 1:1 KKKC:ATP and 2.5:1 KKKC:ATP were prepared as indicated in the main text. Additionally, 2.5:1 KKKC:pAA mixtures were prepared with [NaCl] = 50 and 10 mM.

Each sample (200 µL) was oxidized with a potential of 0.7 V in the standard undivided cell. The time of oxidation was 20 min in the case of pAA-containing samples and 100 min for the samples with ATP. After coacervate formation, each sample was split in four parts and transferred to new wells. An aliquot of each dye (2 µL) was added to every well and mixed via repeated pipetting. The well plate was covered and the mixtures were left to equilibrate overnight. Fluorescence microscopy images were recorded using a 455 nm excitation wavelength for fluoresceine and Brooker's merocyanine, and a 525 nm excitation wavelength for Rhodamine B and Nile red.

Confocal microscopy

The samples for the confocal microscopy were prepared using PVA-coated glass microscope slides to prevent surface wetting. To coat the slides, a 5 % (w/w) partially hydrolysed polyvinyl alcohol (PVA, 146 -186 kDa) solution was spread on top of clean glass slides and was left to dry overnight. A 14 µL aliquot of each sample was added into a spacer (Grace Bio-Labs SecureSeal, 0.12 mm depth) on the microscope slide, and a coverslip was carefully placed on top to minimize evaporation. Confocal imaging was performed using a confocal laser scanning microscope (TCS SP8, Leica) equipped with a 63x/NA 1.40 oil immersion objective (HC PL APO CS2, Leica). Images were captured at the 12-bit depth. Excitation was performed using laser lines of 405, 488, and 552 nm, selected according to the fluorophore used. Emission was collected within the following spectral windows: 550–630 nm for Brooker's merocyanine, 500–550 nm for fluorescein, 550–608 nm for rhodamine B, and 600–650 nm for Nile red. Simultaneous transmission PMT was used to record transmission images. Laser power, scan speed, and signal averaging were optimized to maximize the signal-to-noise ratio. The XYZ spatial resolution was adjusted to satisfy the Nyquist sampling criterion.

Calculation of the partition coefficient (Kp) for model dyes

The partition coefficient of the fluorescent molecules shown in Figure S30 was calculated as the ratio between the fluorescence intensity inside each droplet and in the surrounding dilute phase ($K_p = I_{in}/ I_{out}$) .

For the systems containing Nile Red, the relative values of fluorescence intensity outside, inside and on the border of the droplet were reported in addition to the Kp.

Merging of coacervates with encapsulated dyes

Coacervates based on pAA were prepared by mixing ($KKKC_2$) and a solution containing a mixture of either pAA or ATP, and either Brooker's merocyanine or Nile Red. These samples were imaged directly after preparation.

Coacervates based on ATP were obtained via electrochemical oxidation (0.7 V, 100 minutes) and incubated with Rhodamine B, Nile Red, Fluorescein and Brooker's merocyanine overnight in a microcentrifuge tube. The coacervates were then redispersed by vortexing and deposited in a well-plate, and the same positions were imaged immediately after preparation and after overnight incubation.

The final concentrations were [KKKC] = 1.34 mM or 3.34 mM, [ATP] = 1.34 mM or [pAA] = 4 mM in monomer units, and [dye] = 0.95 µM. All samples contained Tris buffer (50 mM) and NaCl (100 mM for pAA-based coacervates and 10 mM for ATP-based coacervates).

Reduction-mediated destruction of coacervates in presence of dyes

Coacervate samples were prepared electrochemically (0.7 V, 20 min for the KKKC:pAA system, 100 min for the KKKC:ATP system) and incubated with Rhodamine B, Nile Red, Fluorescein and Brooker's merocyanine overnight in microcentrifuge tubes. The samples were then redispersed by vortexing and deposited in a well-plate. The same positions in each sample were imaged before and after the addition of TCEP (19.6 mM). The samples contained [KKKC] = 1.34 mM or 3.34 mM, [ATP] = 1.34 mM or [pAA] = 4 mM in monomer units, and [dye] = 0.95 µM. All samples contained Tris buffer (50 mM) and NaCl (100 mM for pAA-based coacervates and 10 mM for ATP-based coacervates).

Supplementary Figures

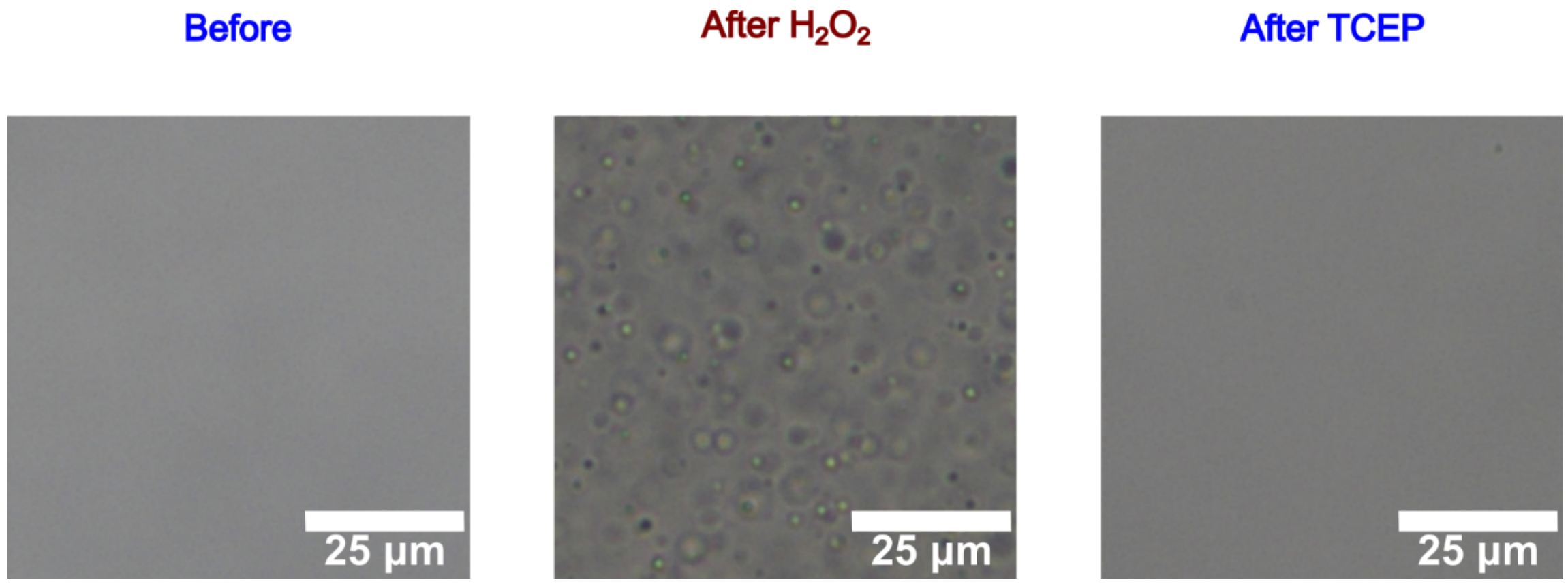

Figure S1. Micrographs of a sample containing KKKC and pAA in a 2.5:1 ratio – after mixing, immediately after the addition of H_2O_2 (29.4 mM, 8.8 eq.), and immediately after addition of TCEP (32.1 mM, 9.6 eq.). The concentrations of polyelectrolytes were [KKKC] = 3.34 mM, [pAA] = 4 mM (in monomer units). The samples contained Tris buffer (50 mM, pH = 7.4) and NaCl (100 mM). The scale bars are 25 µm.

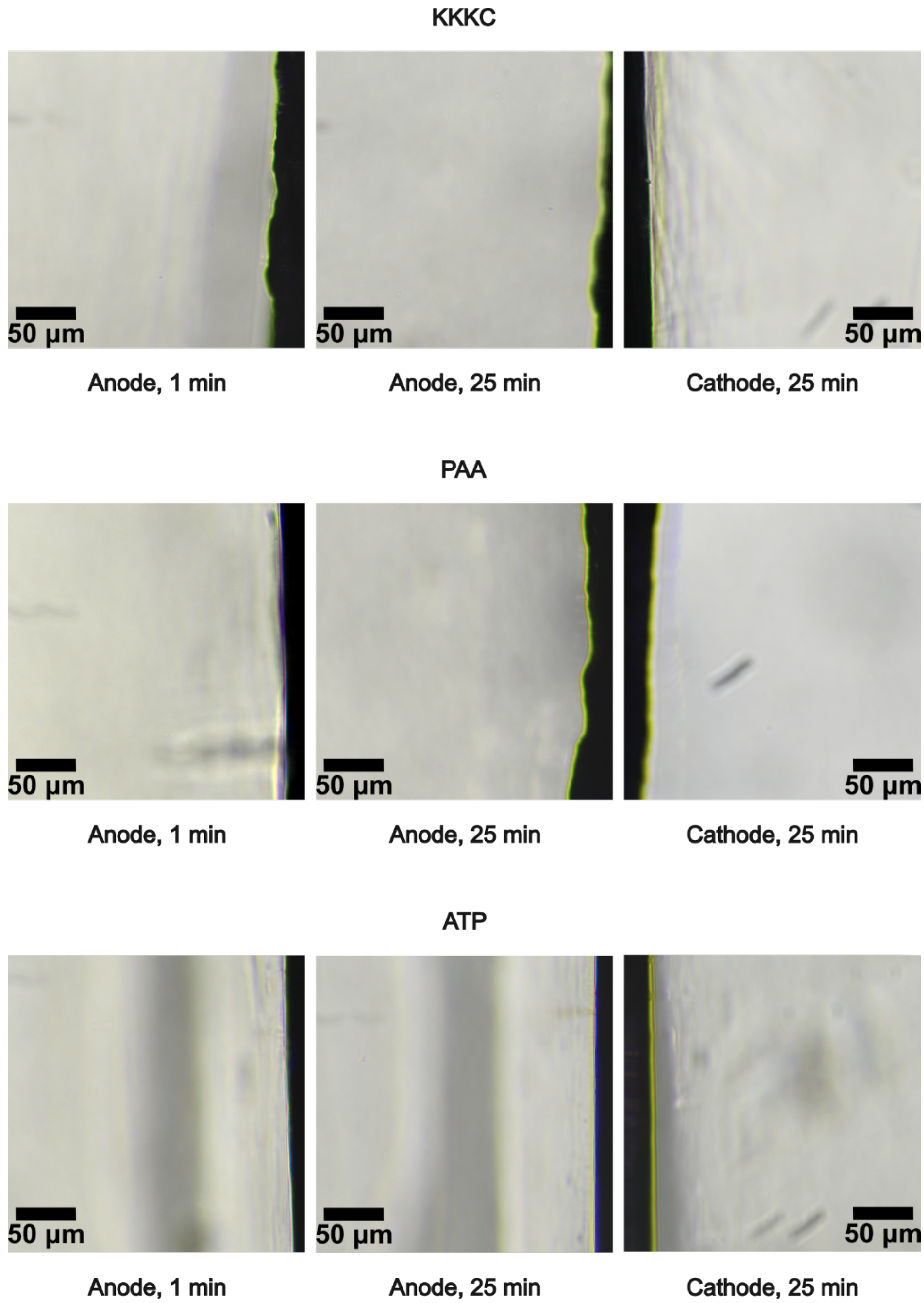

Figure S2. Microscope images of the area next to the electrodes showing the absence of the coacervate formation upon application of an electric potential (0.7 V) in the following control solutions: KKKC (1.34 mM in 10 mM NaCl), pAA (4 mM in 100 mM NaCl), ATP (1.34 mM in 10 mM NaCl). All solutions contained 50 mM Tris buffer (pH = 7.4). The scale bars are 50 µm.

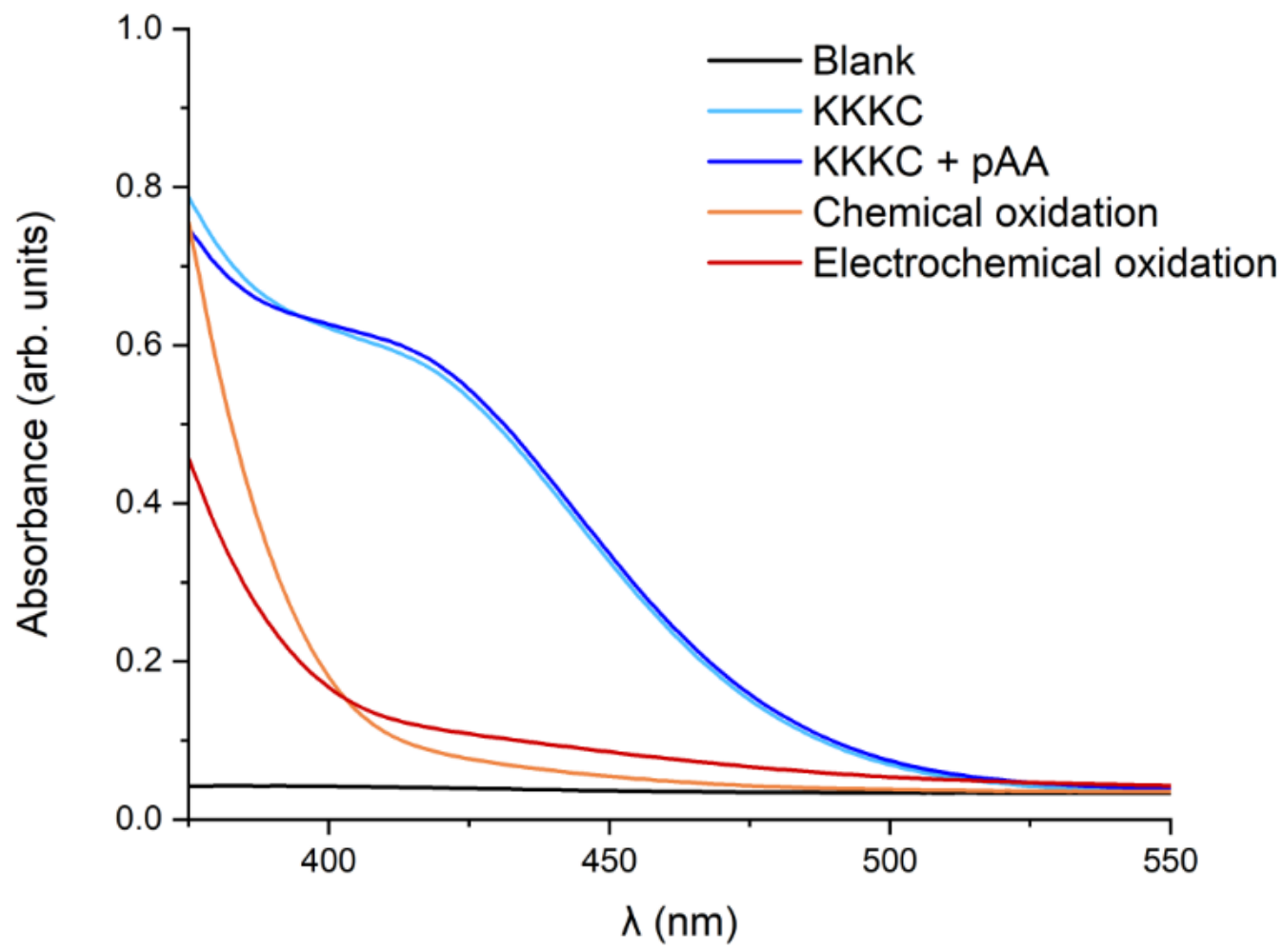

Figure S3. Ellman's test for the UV-Vis detection of thiols. Solutions of KKKC and KKKC + pAA show a band at 412 nm, characteristic to the reaction between free thiols and DTNB (5,5′-dithiobis-(2-nitrobenzoic acid)). No thiols are detected in the chemically or electrochemically oxidized KKKC + pAA samples, indicating that all thiol groups had been completely oxidized. [KKKC] = 0.2 mM, [pAA] = 0.08 mM (in charge units). All samples contained 50 mM Tris buffer (pH = 7.4), 100 mM NaCl, and 0.5 mM of DTNB. The chemically oxidized sample contained 2 equiv. of $NaBO_3$, and the electrochemically oxidized sample was preparing by applying a 0.9 V potential for 30 minutes to a KKKC/pAA mixture.

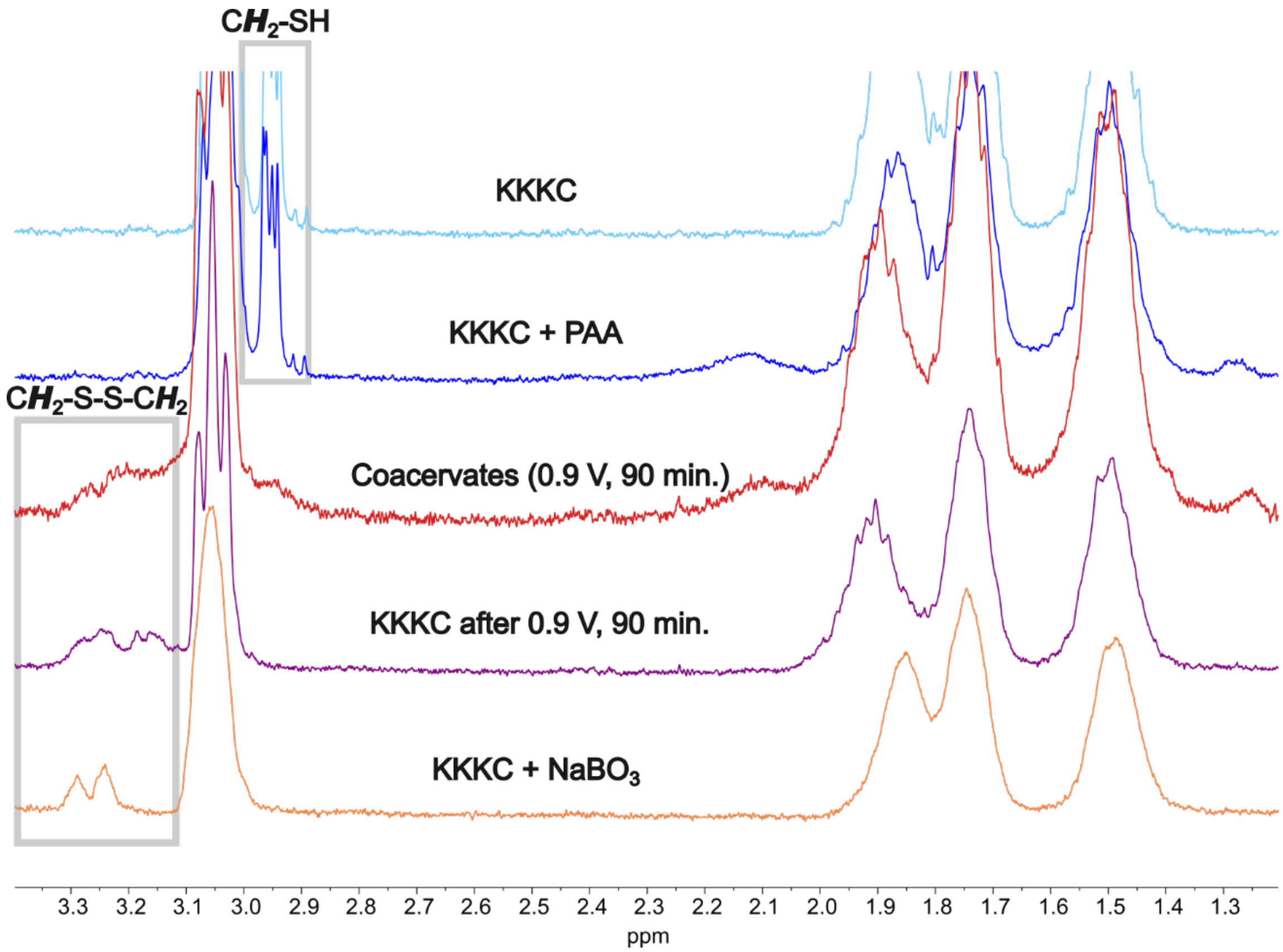

Figure S4. ^{1}H-NMR spectra (300 MHz, D_2O) of fresh KKKC solution, polyelectrolyte mixture, coacervates prepared by electrochemical oxidation and controls with electrochemically and chemically oxidized KKKC. The signals corresponding to the CH_2 protons next to the thiol/disulfide group are highlighted. The signals at 2.2-2.1 ppm and 1.3-1.2 ppm correspond to the protons of pAA. The signals between 2.0 and 1.4 ppm and at 3.1-3.0 ppm correspond to the protons of the lysine side-chains.

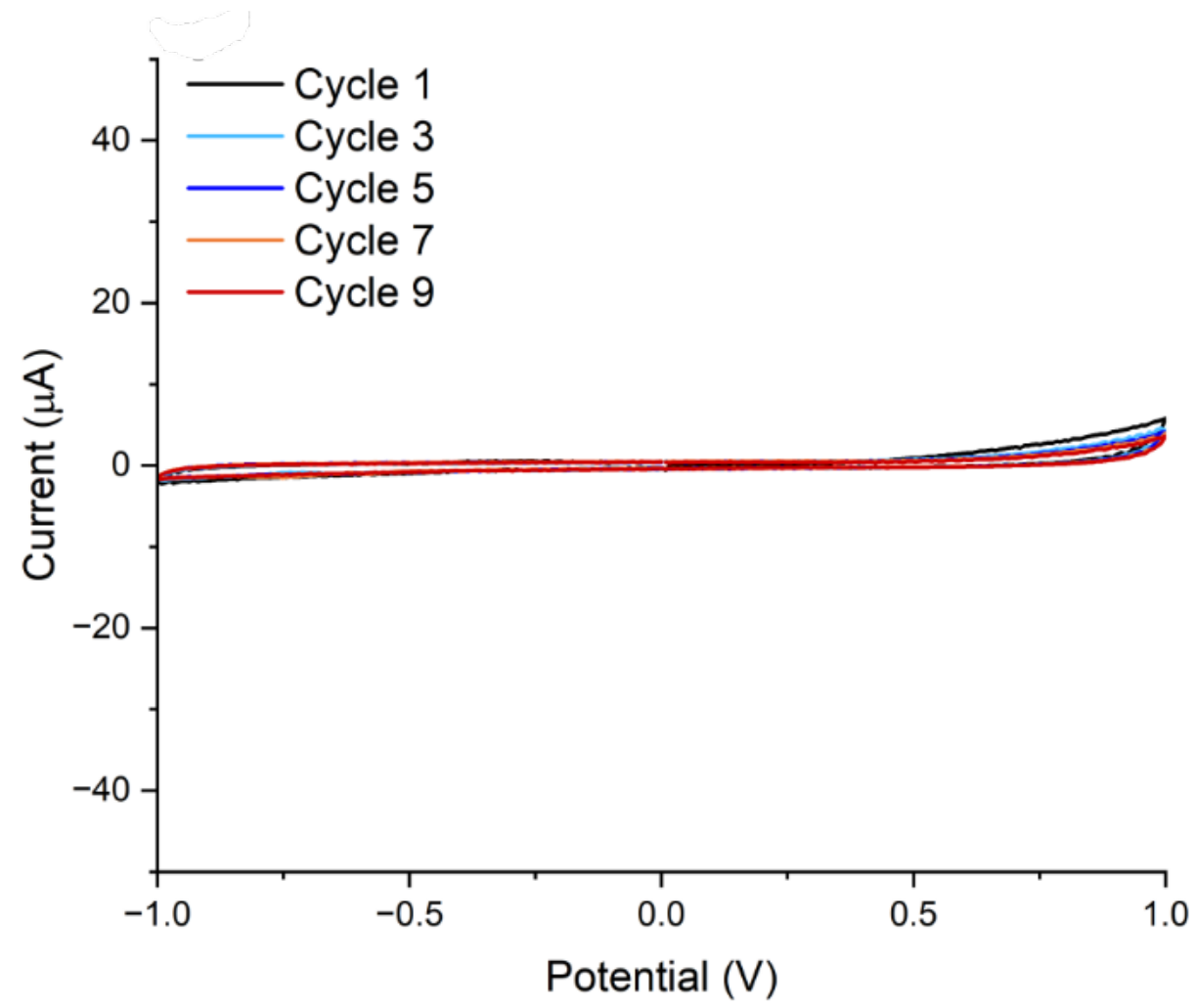

Figure S5. Cyclic voltammetry curve of KKKC, recorded with a GCE working electrode (vs Ag/AgCl). No electrochemical response could be detected within the working window of the electrode. [KKKC] = 1.67 mM. The solution contains Tris buffer (50 mM) and NaCl (100 mM).

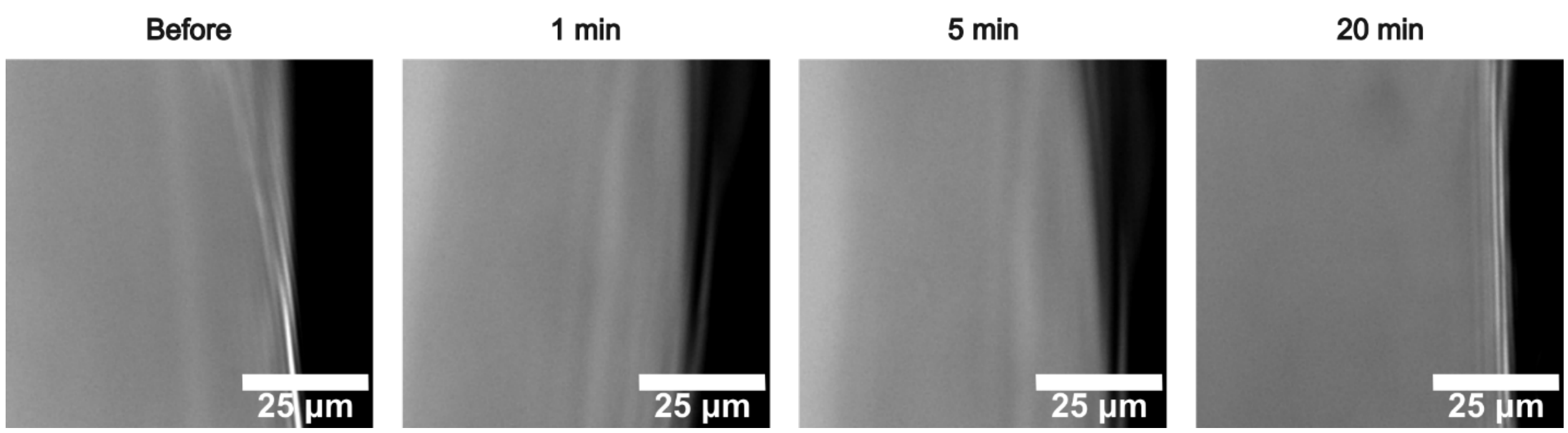

Figure S6. Micrographs of 2.5:1 KKKC:pAA mixtures in an electrochemical setup with glassy carbon flat electrodes. The micrographs were recorded next to the surface of the anode, before and during the application of an electric potential (0.7 V). [KKKC] = 3.34 mM, [pAA] = 4 mM (in monomer units). No coacervate droplets were detected throughout the experiment. The scale bars are 25 µm.

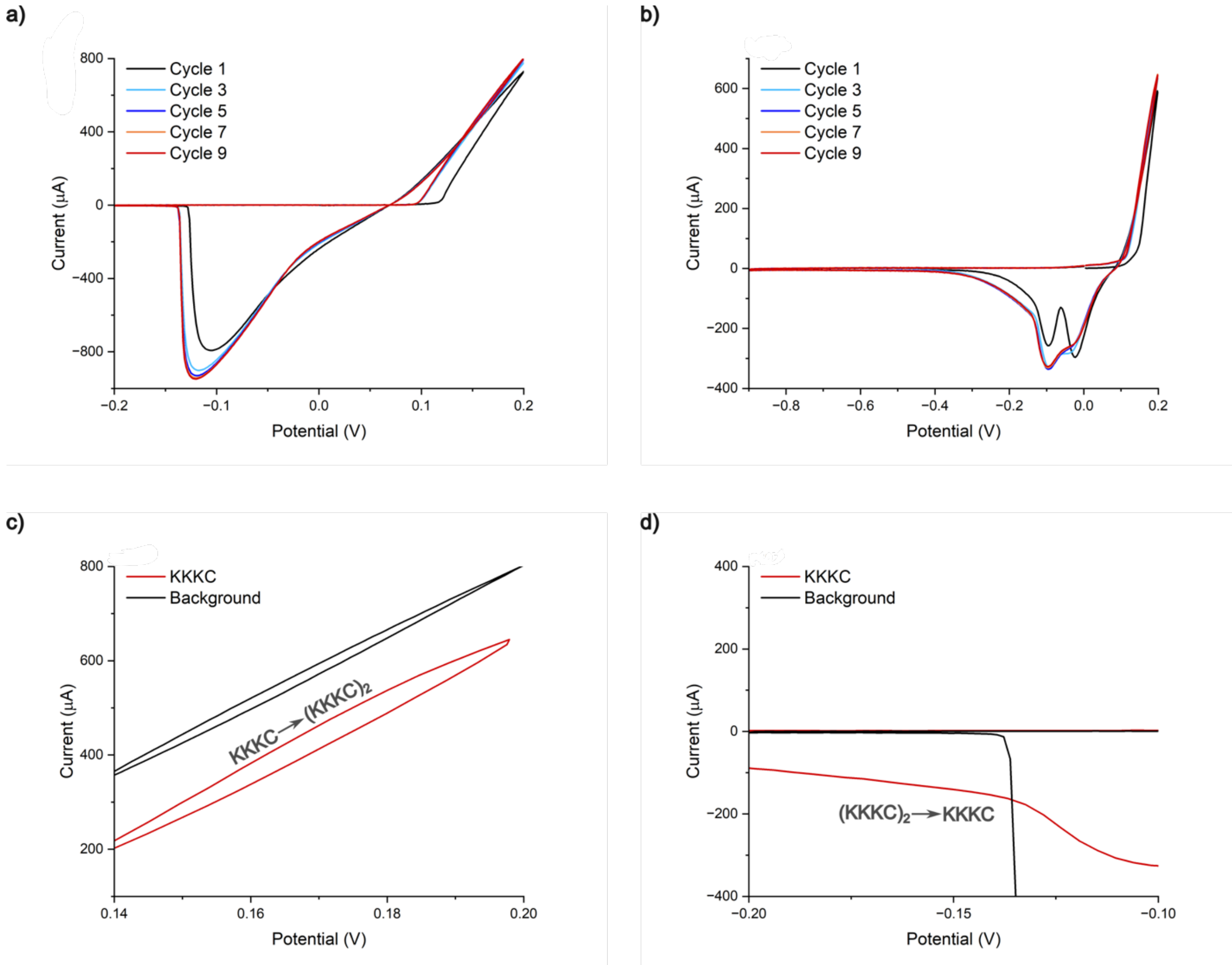

Figure S7. Cyclic voltammetry study of KKKC using a silver working electrode (vs Ag/AgCl). a) CV curves of the background electrolyte, showing the anodic oxidation of silver (> 0.1 V) and a cathodic peak corresponding to the reduction of trace amounts of dissolved oxygen. b) CV curves of KKKC in the same conditions. In addition to the signals that were already present in the background CV, two additional broad peaks can be observed: a new oxidation band between 0.14 and 0.20 V, and a reduction band between -0.1 and -0.3 V. c) Comparison between the CV curves of background electrolyte and KKKC (Cycle 9), zoomed in to the region corresponding to anodic oxidation. d) Comparison between the CV curves of background electrolyte and KKKC (Cycle 9), zoomed in the region corresponding to cathodic reduction. [KKKC] = 1.67 mM. Both solutions contain Tris buffer (50 mM) and NaCl (100 mM).

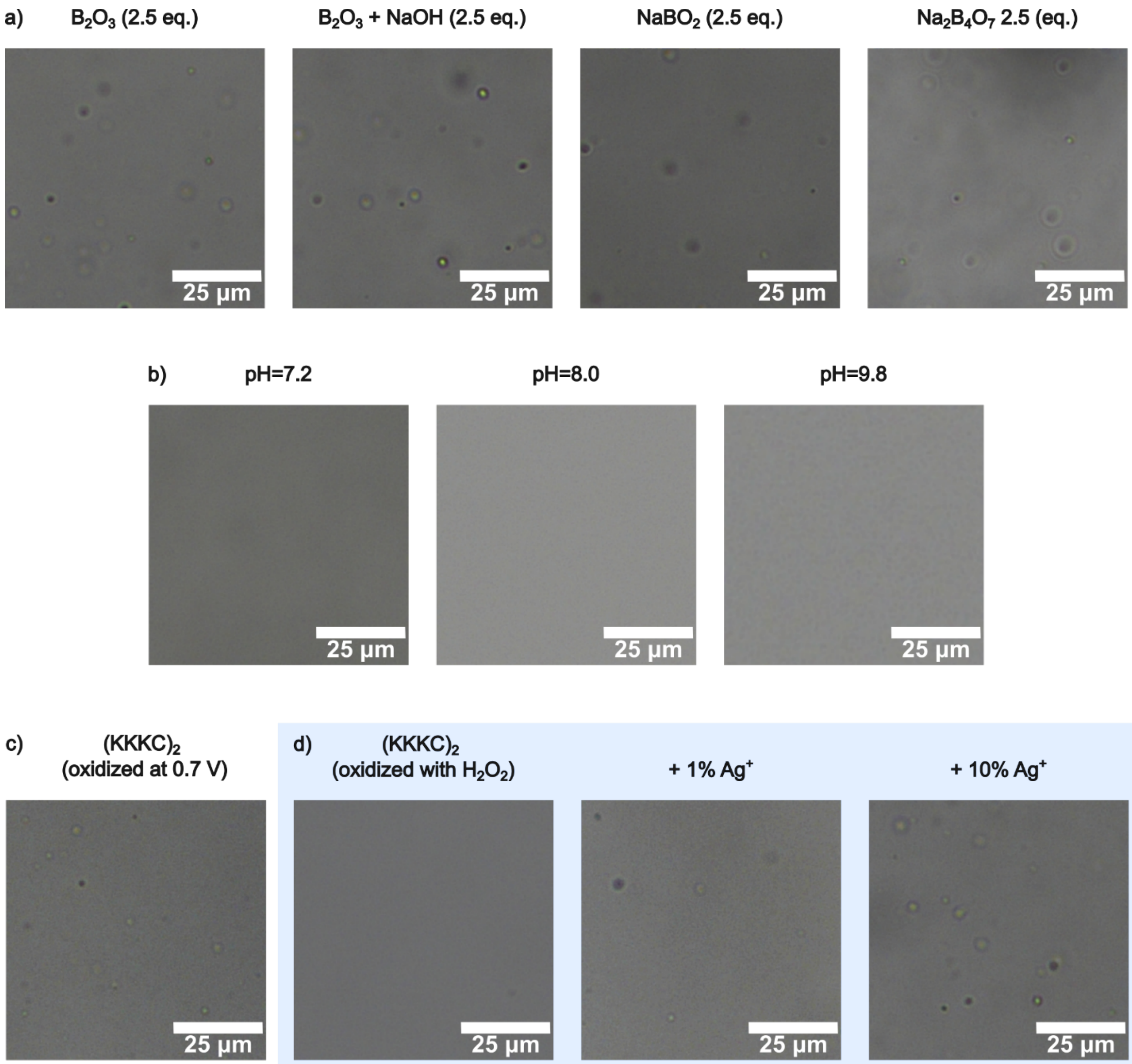

Figure S8. Micrographs of 1:1 KKKC:ATP mixtures in various conditions. a) $(KKKC)_2$:ATP coacervates prepared via electrochemical oxidation (0.7 V, 1 hour), followed by the addition of boric anhydride (B_2O_3), a mixture of B_2O_3 and NaOH, sodium metaborate ($NaBO_2$), or sodium tetraborate ($Na_2B_4O_7$) (3.35 mM of each salt). b) KKKC:ATP mixtures prepared at different pH values and incubated with sodium perborate (3.35 mM, 2.5 eq.) for 2 hours. c) $(KKKC)_2$:ATP coacervates prepared by mixing electrochemically-prepared $(KKKC)_2$ (0.7 V, 40 min) and ATP. d) $(KKKC)_2$:ATP mixtures prepared by chemical oxidation of KKKC with H_2O_2, in presence of different concentrations of Ag^+. Silver ions were added from a 5 mM stock solution of $AgNO_3$ (1 or 10 molar % of Ag^+ with respect to KKKC). [KKKC] = 1.34 mM, [ATP] = 1.34 mM (in monomer units). The scale bars are 25 µm.

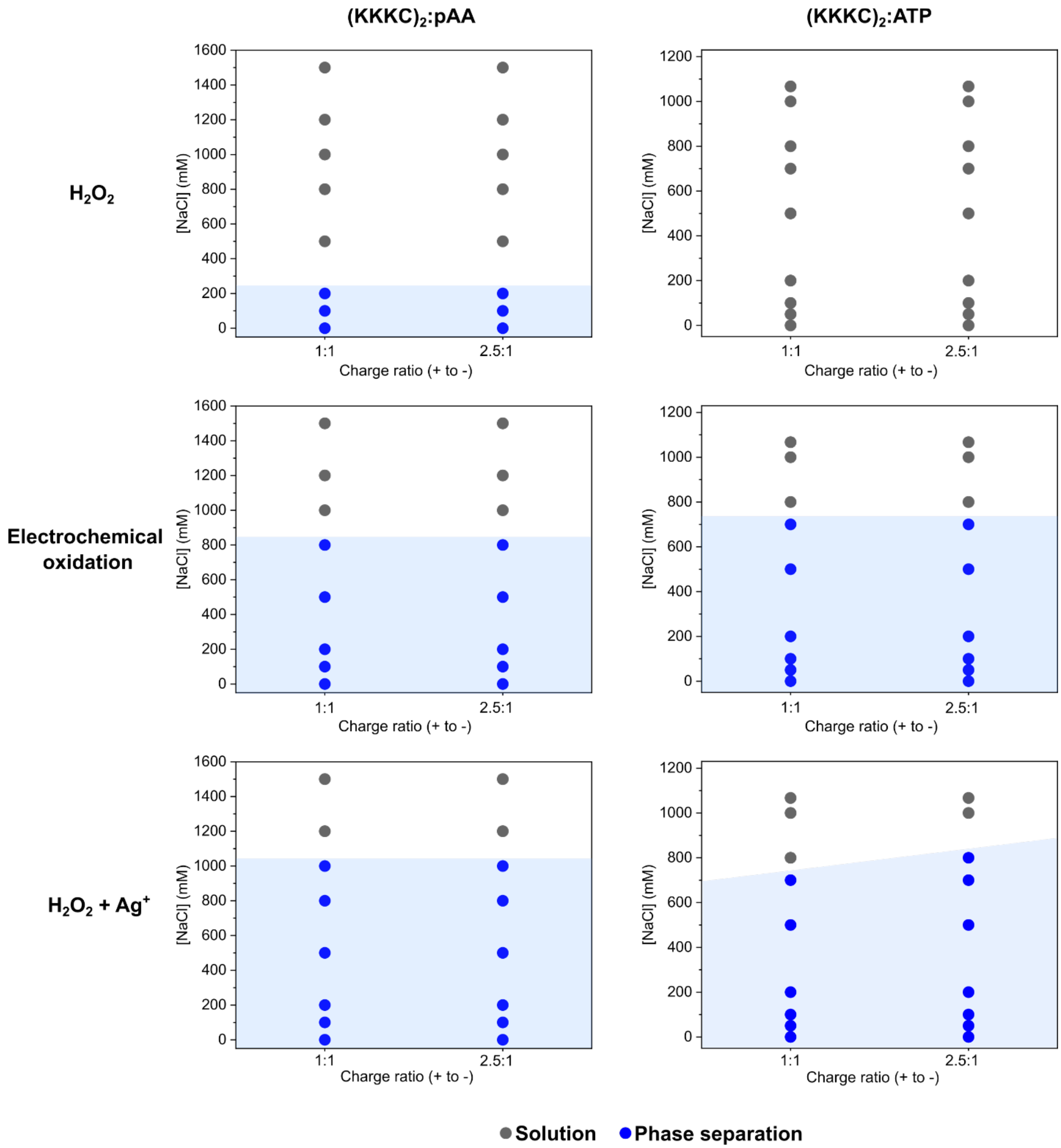

Figure S9. Phase diagrams of (KKKC)$_2$/pAA and (KKKC)$_2$/ATP coacervates prepared in three different conditions: 1) Chemical oxidation of KKKC with H_2O_2 (22 eq.) followed by mixing with ATP or pAA. 2) Electrochemical oxidation of KKKC followed by mixing with ATP or pAA. 3) Chemical oxidation of KKKC followed by addition of $AgNO_3$ (0.134 mM). [KKKC] = 1.34 mM or 3.34 mM (for the 1:1 and 2.5:1 mixtures, respectively), [pAA] = 4 mM, [ATP] = 1.34 mM (in monomer units). The same titrations were repeated in reducing conditions (without H_2O_2 and with no electrochemical oxidation, in presence of 3.35 mM of TCEP), and no phase separation could be detected at any salt concentration or mixing ratio.

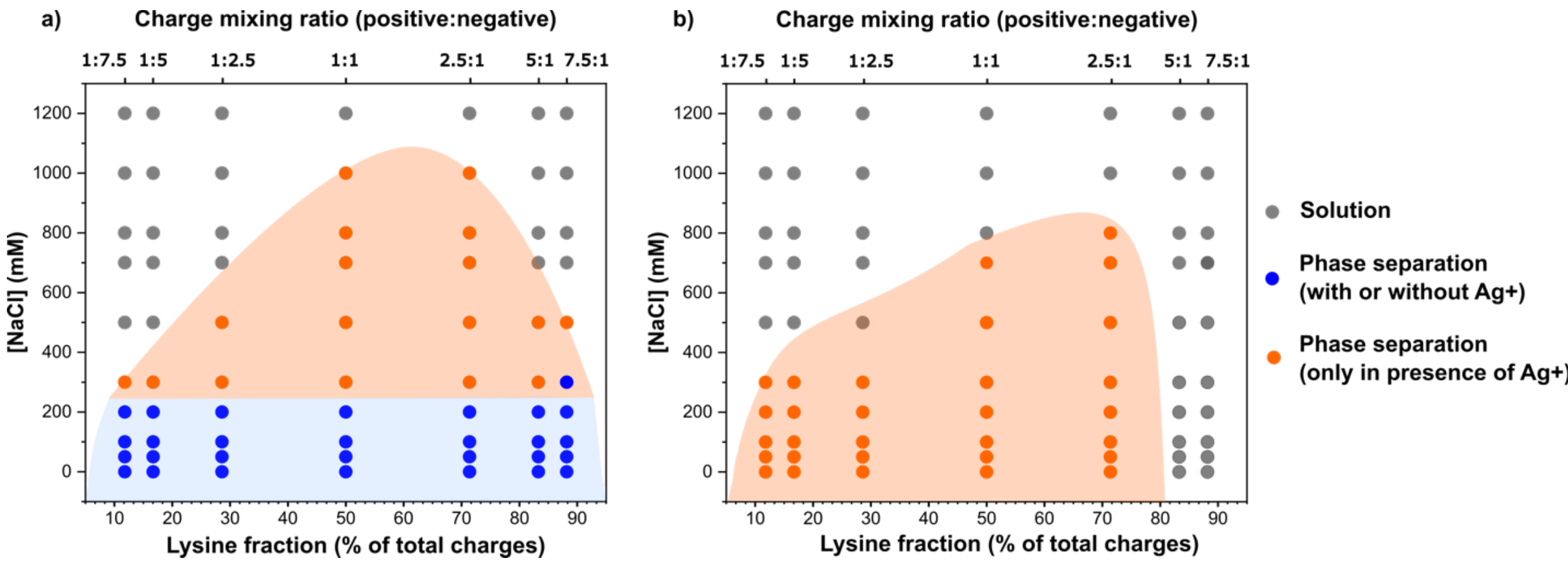

Figure S10. Phase diagrams of $(KKKC)_2$/pAA and $(KKKC)_2$/ATP coacervates prepared with various charge ratios for the systems a) $(KKKC)_2$/pAA, and b) $(KKKC)_2$/ATP. The samples in presence of Ag^+ contain 0.134 mM of $AgNO_3$.

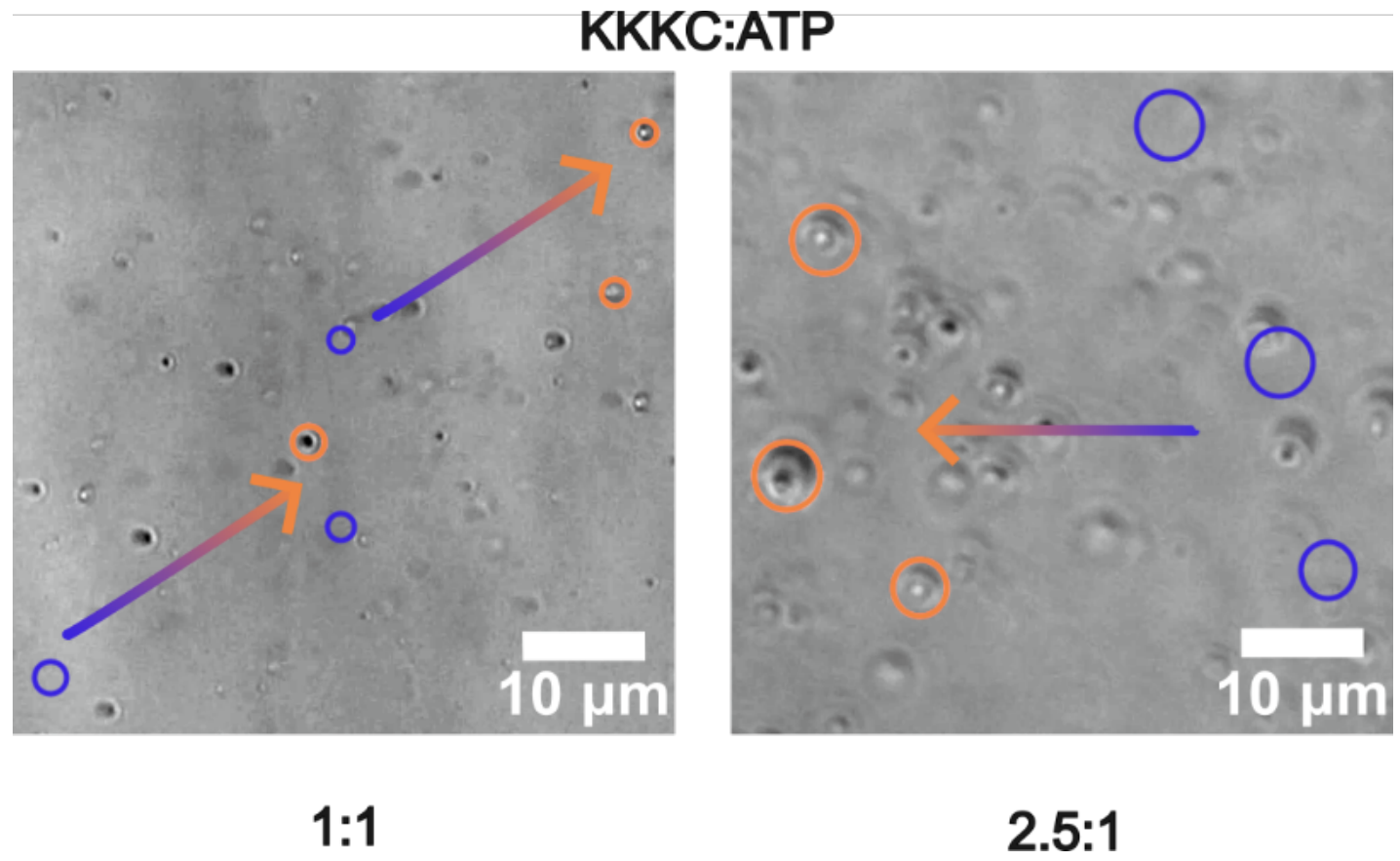

Figure S11. Electrophoretic movement of KKKC/ATP coacervates at different mixing ratios. The numbers indicated in the images represent the mixing ratio between KKKC and ATP in charge units. [KKKC] = 1.34 mM or 3.34 mM, [ATP] = 1.34 mM. All samples contained Tris buffer (50 mM) and NaCl (10 mM). The strength of the electric field was 0.95 V/cm. The scale bars are 10 µm.

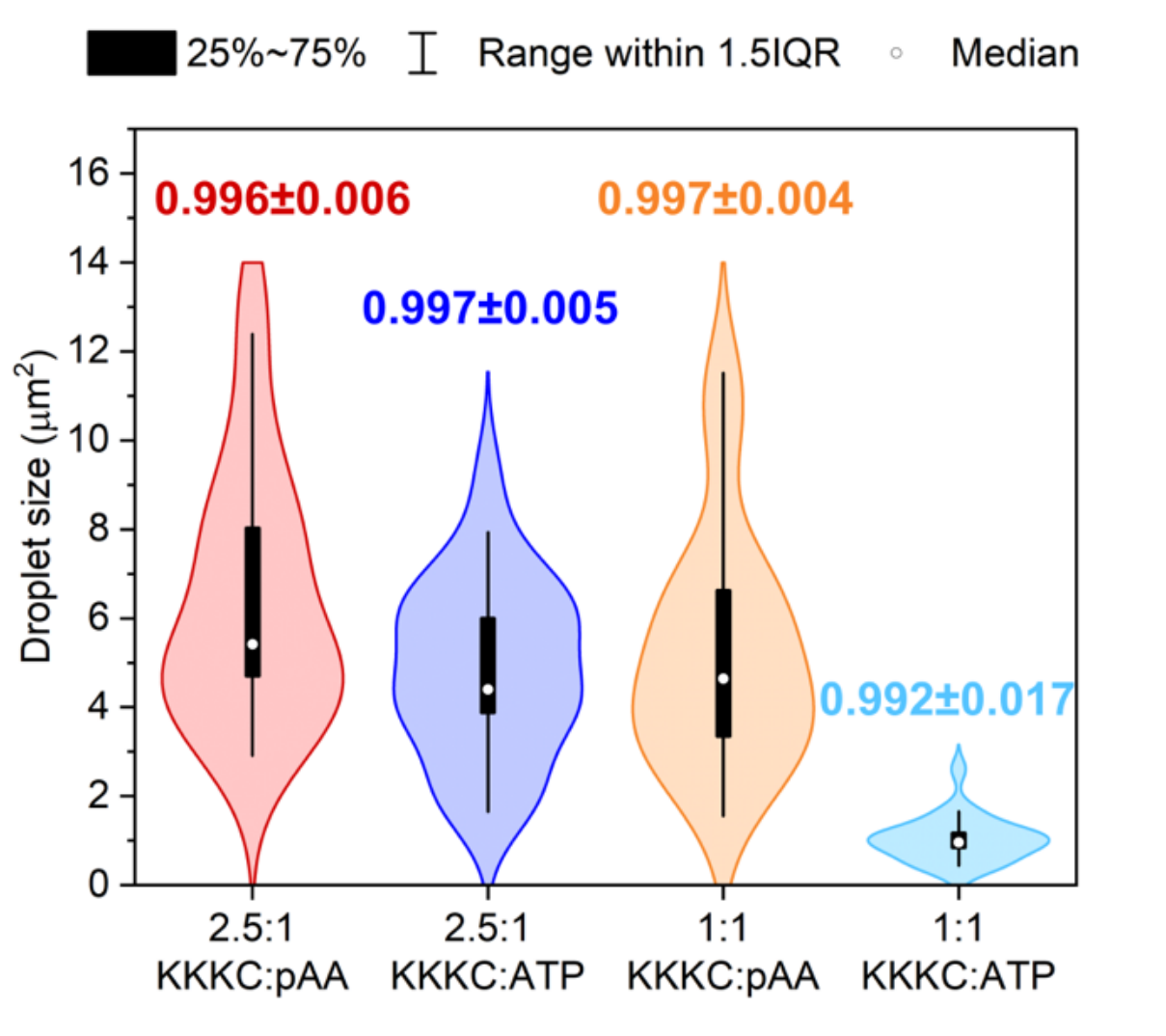

Figure S12. Size distributions of coacervates prepared by the electrochemical oxidation (0.7 V) of KKKC in presence of either pAA or ATP in different mixing ratios. 25 droplets were analyzed for each sample. The circularity of the droplets is reported above the corresponding violin plots (as average ± standard deviation). [KKKC] = 1.34 mM or 3.34 mM (for 1:1 and 2.5:1 mixtures respectively), [pAA] = 4 mM, [ATP] = 1.34 mM (in monomer units). The samples contained Tris buffer (50 mM, pH = 7.4) and NaCl (10 mM for ATP samples, 100 mM for pAA samples).

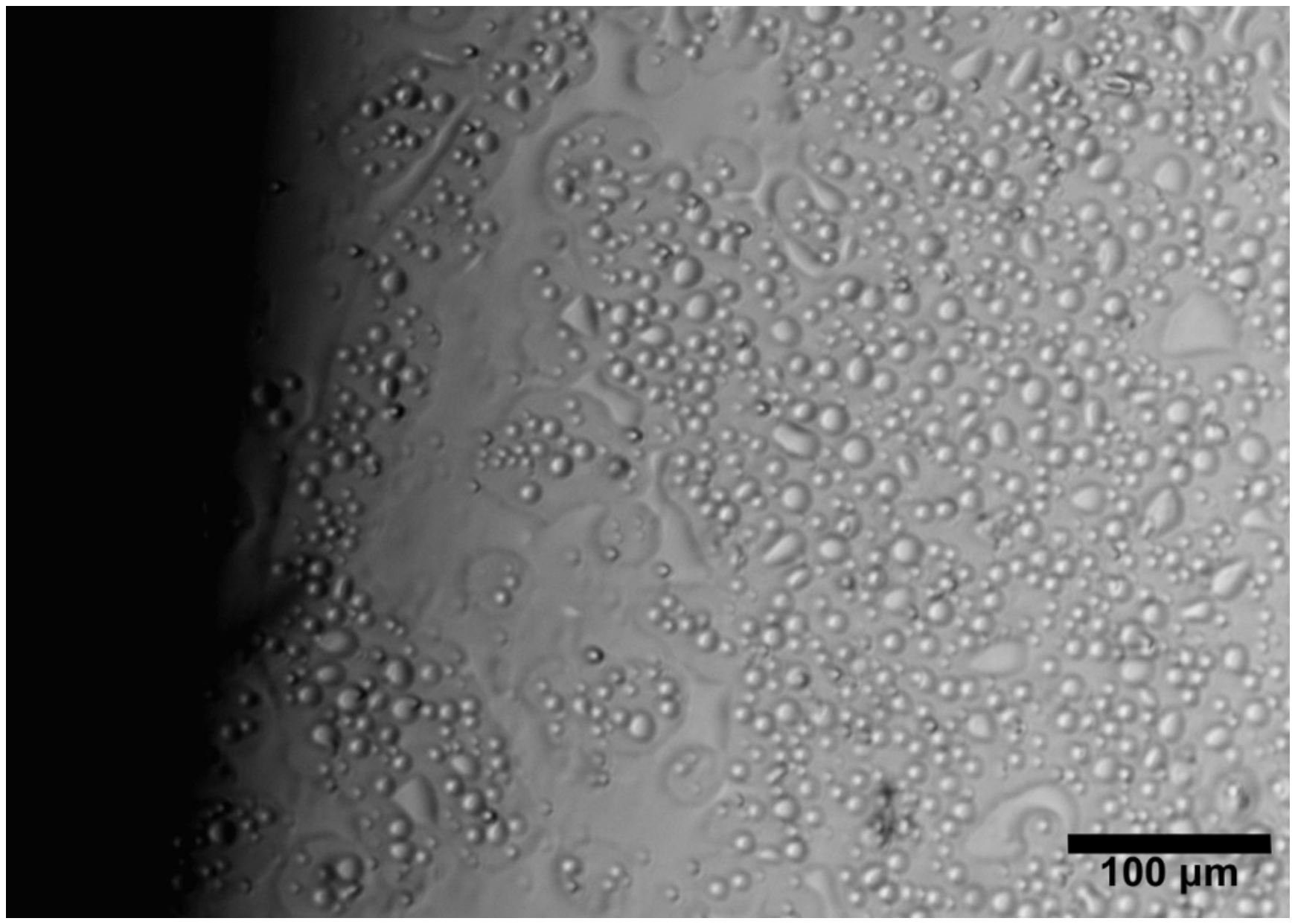

Figure S13. Micrograph of the bottom of the cell next to the cathode, ~20 minutes after the application of a 0.7 V potential. The accumulation of a continuous coacervate phase radiating from the electrode indicates that electrochemical reduction of $(KKKC)_2$ contained in the coacervate droplets is slower than their coalescence and sinking. [KKKC] = 3.34 mM, [pAA] = 4 mM (in monomer units). The ratio between positive and negative charges was 2.5:1. The scale bar is 100 µm.

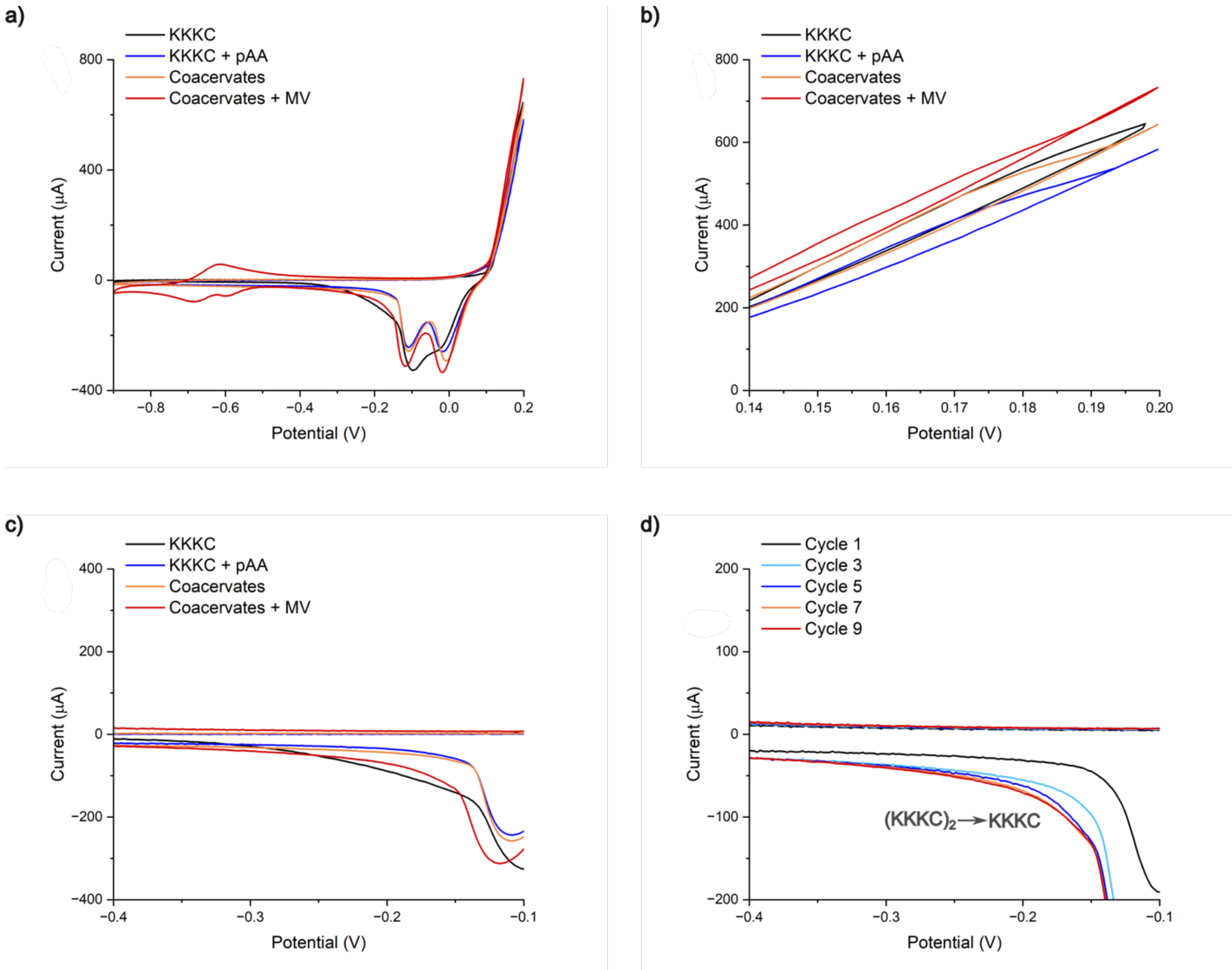

Figure S14. CV curves (Cycle 9) of KKKC in presence of pAA and MV. a) Full CV curves. b) Region corresponding to KKKC oxidation. The amplitude of the peak overlapping with silver oxidation is not significatively different between different samples. c) Region corresponding to the reduction of $(KKKC)_2$. The current of the $(KKKC)_2 \rightarrow$KKKC band is reduced in the samples containing pAA, and restored when MV is added. d) Consecutive CV cycles in a sample containing coacervates and MV. We hypothesize that several cycles of oxidation / reduction mediated by MV dissolve the coacervate in the surface of the electrode, releasing free $(KKKC)_2$. [KKKC] = 1.67 mM. [pAA] = 2 mM in monomer units. All solutions contain Tris buffer (50 mM) and NaCl (100 mM).

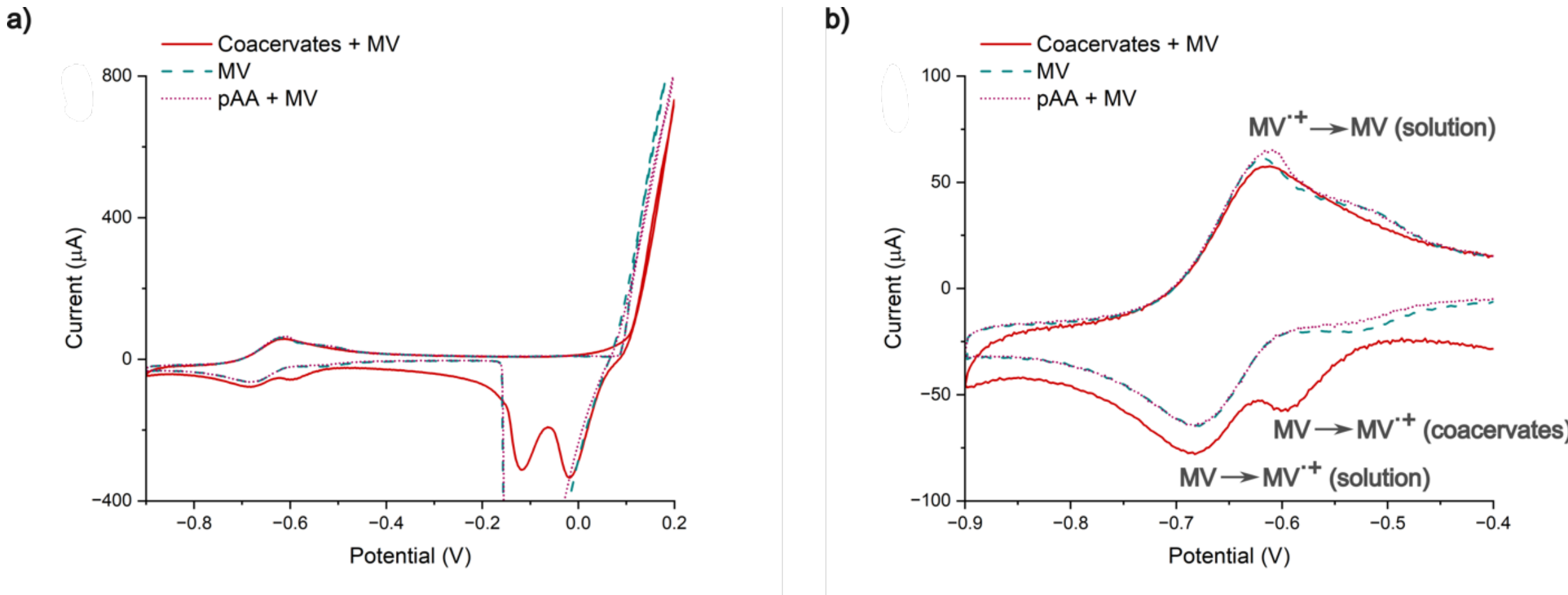

Figure S15. CV curves (Cycle 9) of MV in samples containing either coacervates or pAA. a) Full CV curves. b) Region corresponding to MV oxidation and reduction. The apparently irreversible cathodic band at -0.6 V appears only in the sample containing coacervates, supporting the hypothesis that it corresponds to the oxidation of MV dissolved in the coacervate phase. [KKKC] = 1.67 mM. [pAA] = 2 mM in monomer units, [MV] = 3.34 mM. All solutions contain Tris buffer (50 mM) and NaCl (100 mM).

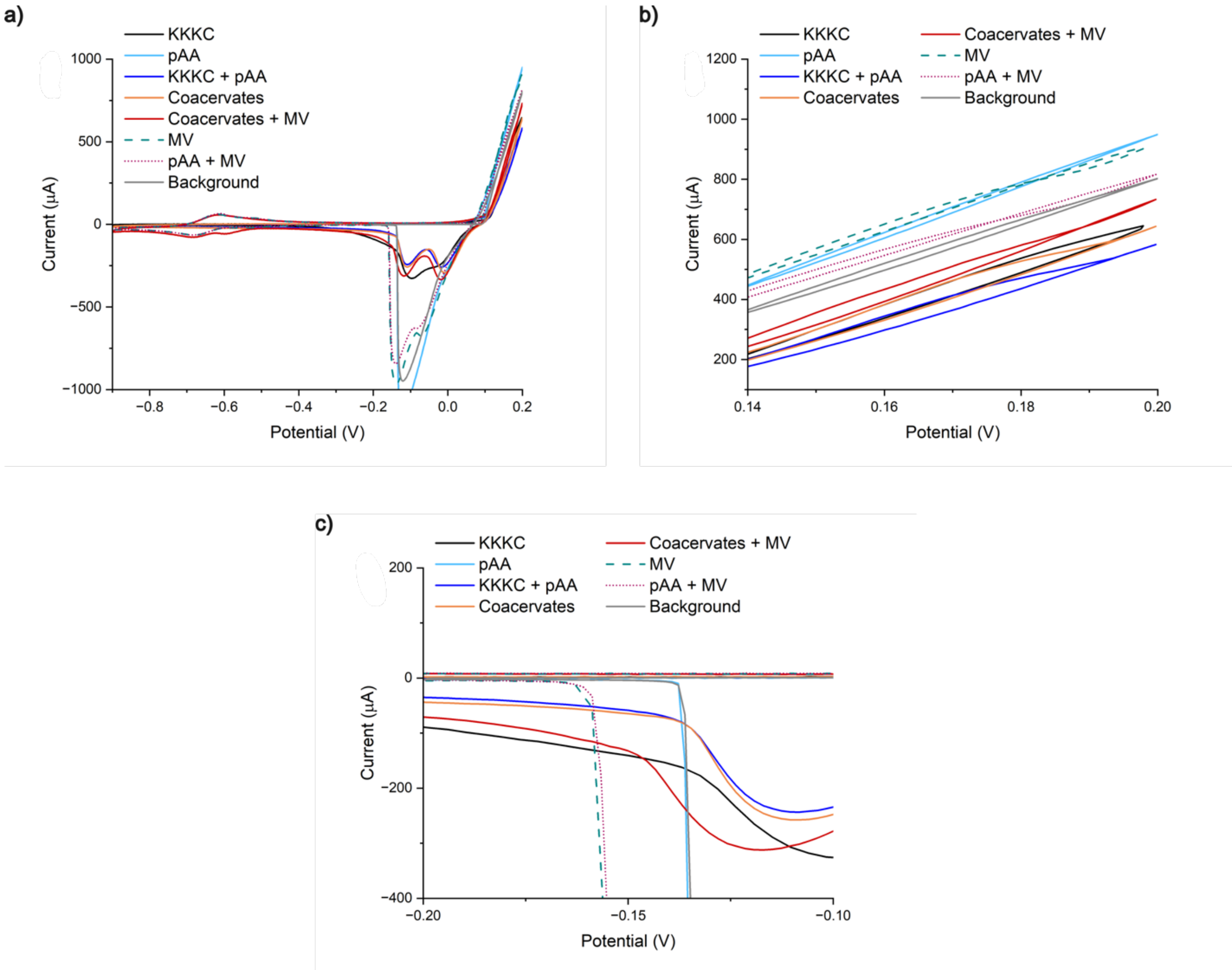

Figure S16. Comparison of the CV curves (Cycle 9) of all samples in this study. a) Full CV curves. b) Region corresponding to KKKC oxidation. c) Region corresponding to $(KKKC)_2$ reduction. The concentrations and conditions for each experiment are indicated in the Supplementary Methods.

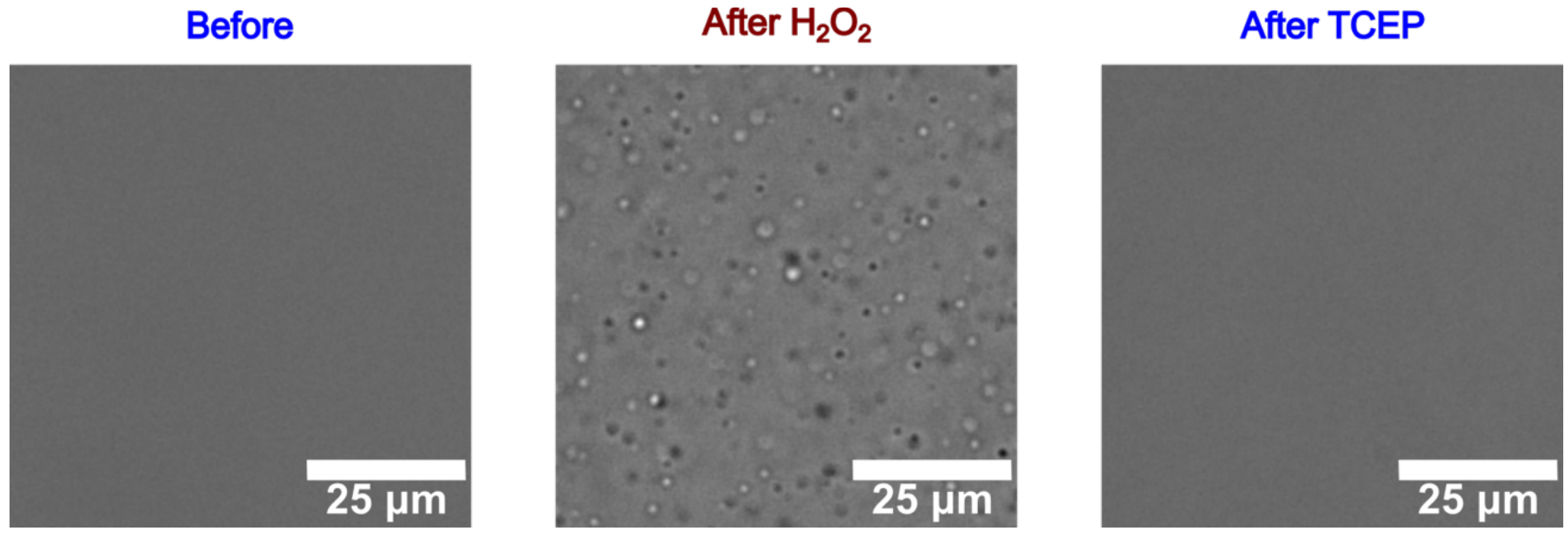

Figure S17. Micrographs of a sample containing KKKC and pAA in a 2.5:1 ratio and MV. The micrographs were recorded upon mixing; immediately after the addition of H_2O_2 (29.4 mM, 8.8 eq.); and immediately after the addition of TCEP (32.1 mM, 9.6 eq.). [KKKC] = 3.34 mM, [pAA] = 4 mM (in monomer units), [MV] = 6.68 mM. The samples contain Tris buffer (50 mM, pH = 7.4) and NaCl (100 mM). The scale bars are 25 µm. No significant differences can be observed between the coacervates formed in this case and those formed without MV (Figure S1).

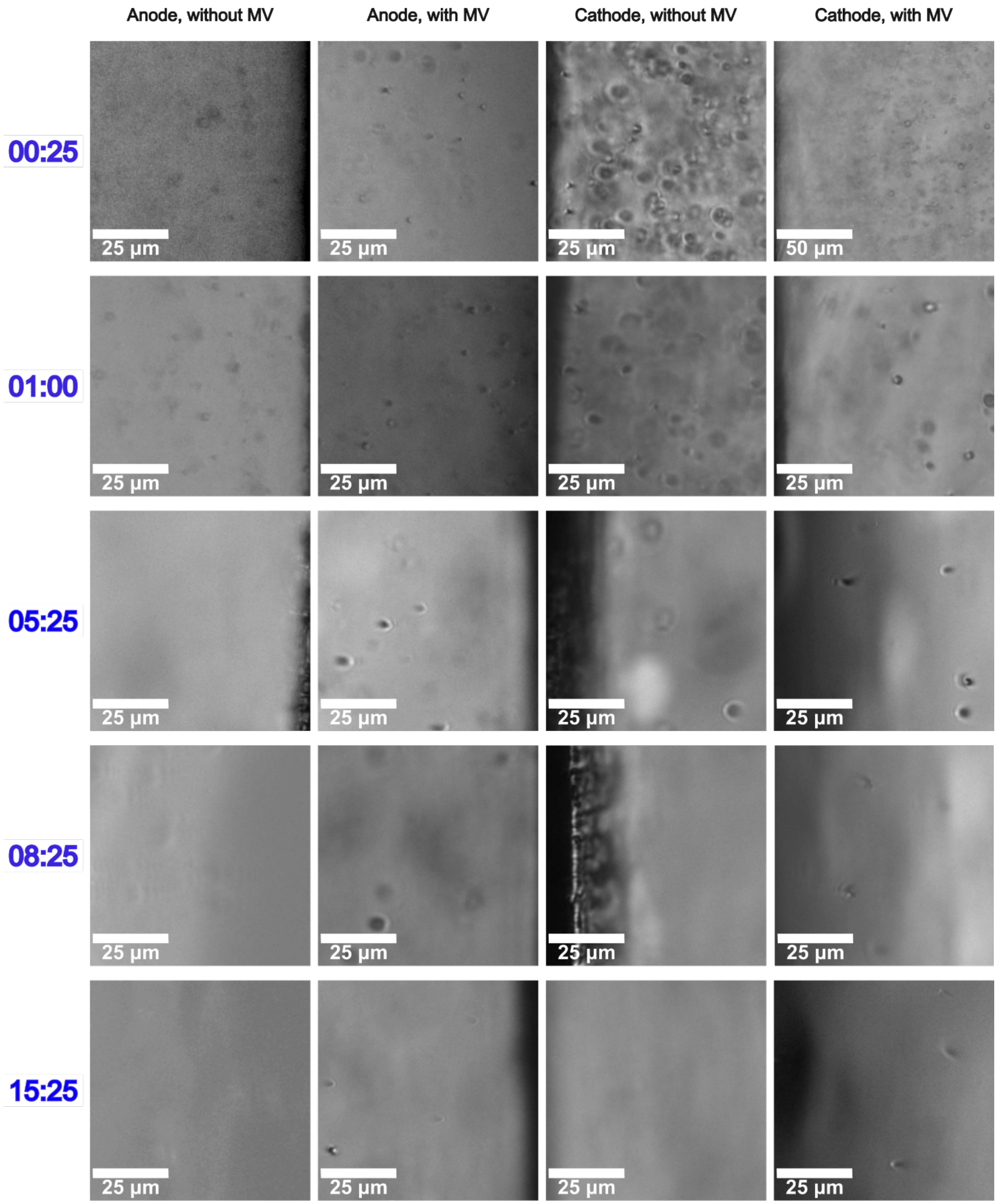

Figure S18. Micrographs of $(KKKC)_2$/pAA coacervates (2.5:1) next to the anode and cathode in presence and in absence of MV, at different times during the application of an electric potential (0.7 V). [KKKC] = 3.34 mM, [pAA] = 4 mM, [MV] = 6.68 mM. The size of the scale bars is indicated in the images. Times are reported as hh:mm.

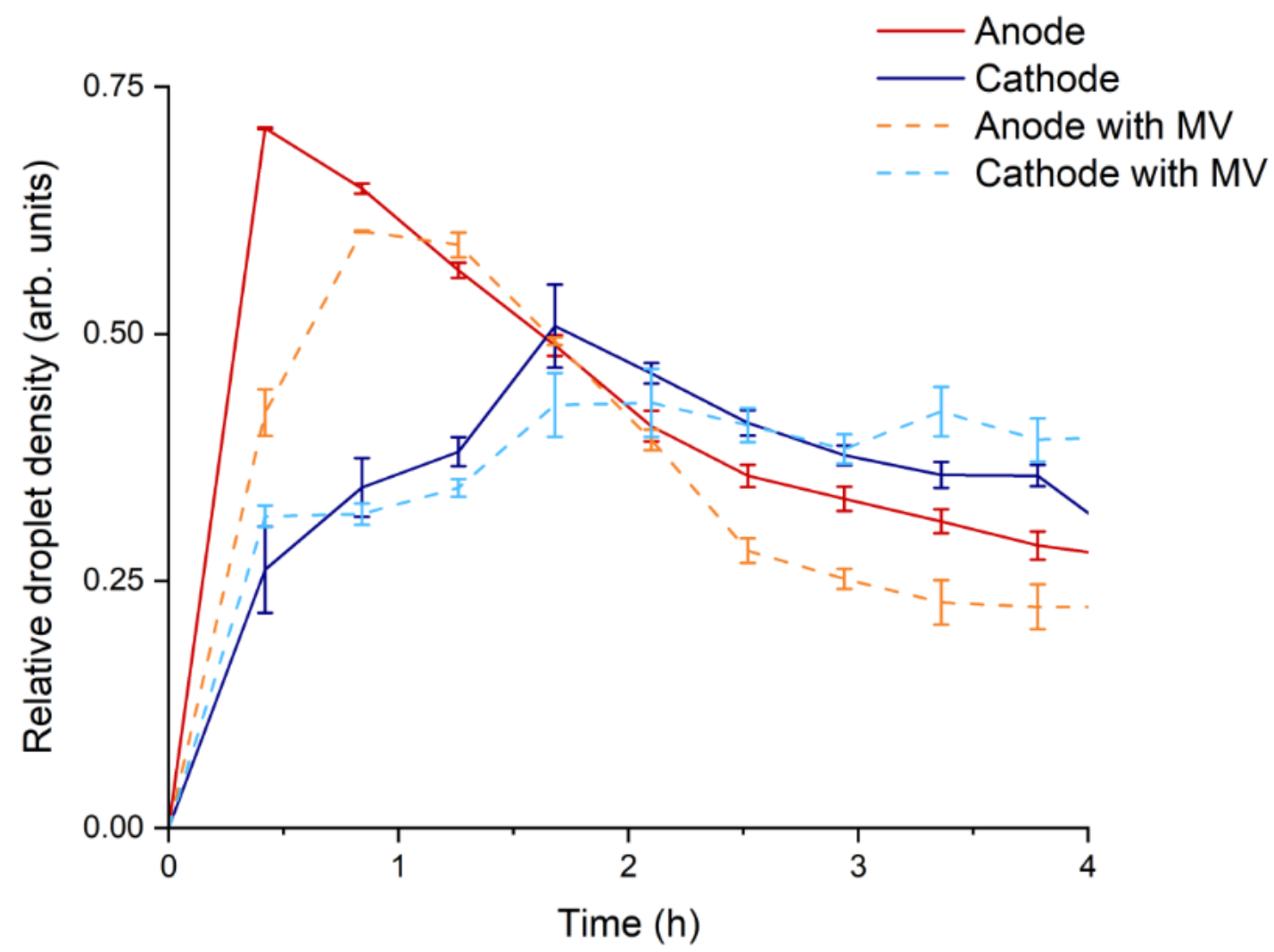

Figure S19. Relative droplet density in 2.5:1 KKKC:pAA samples in presence and absence of MV, upon continuous application of an electric potential (0.7 V). The proximity of the anode and the cathode were imaged separately, and the droplet density was calculated from the average grey value of different areas of the image (see Supplementary Methods). After the first 4 h, the values for all systems reach a plateau, where the gray value is predominantly affected by a layer of coalesced droplets on the background, and the impact of individual droplets being formed and moving is negligible. After this time, the number of droplets was counted manually (see Figure 5 in the Main Text). The reported data represent the average of three different areas at the same distance from the electrode, and the error bars represent the standard deviation (n=3).

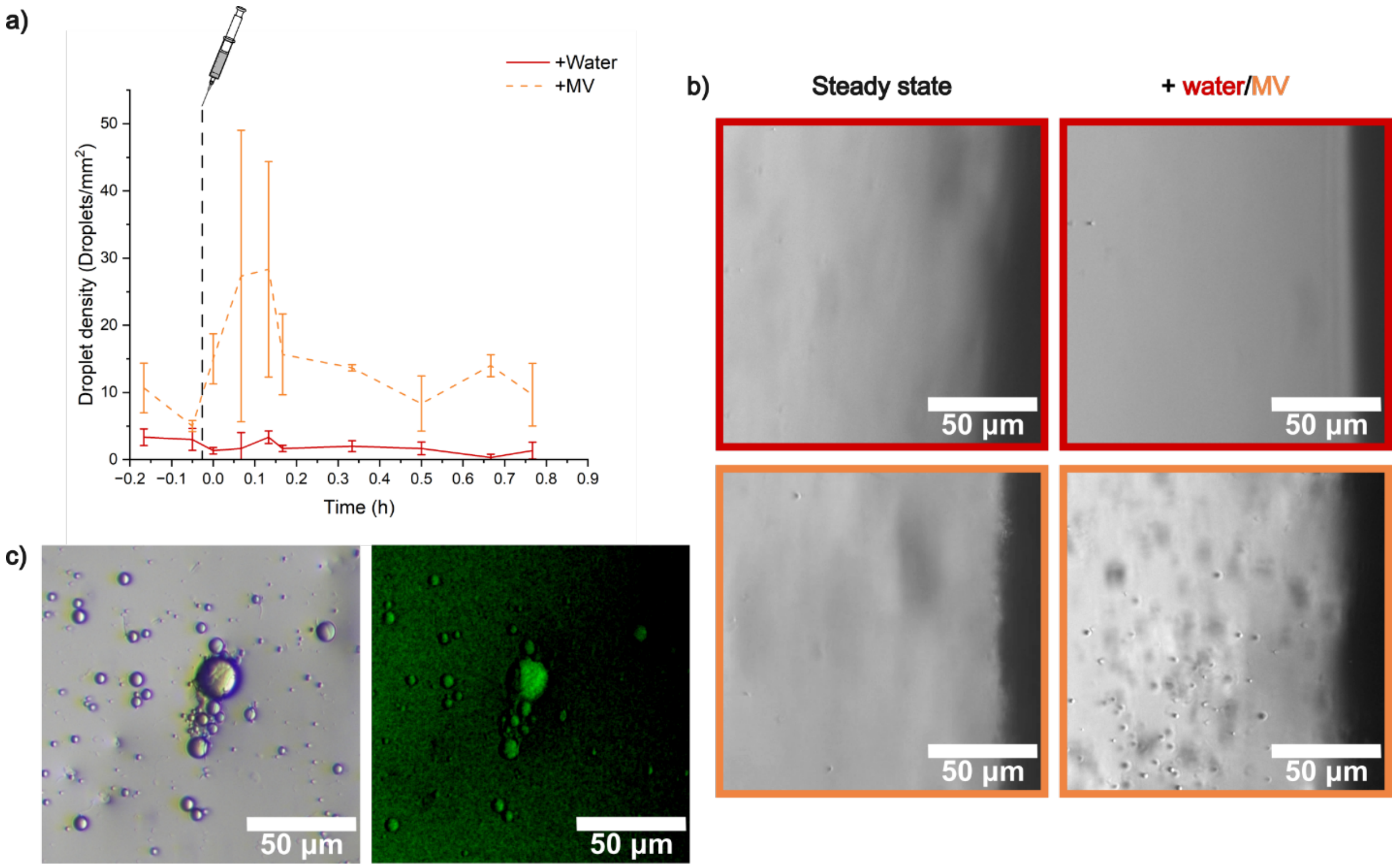

Figure S20. Regeneration of coacervates by addition of MV, after KKKC had been depleted from the anode. a) Average droplet density in the proximity of the anode before and after the addition of MV (orange dashed line). An identical sample, to which water was added instead of MV, is represented in red. The reported numbers correspond to the average of three 100 x 100 µm areas next to the surface of the electrode, and the error bars to their standard deviation (n=3). b) Micrographs of the area next to the anode in both samples, before and after addition of MV or water. c) Brightfield and fluorescence micrographs of coacervates in the bottom of the cell, showing the encapsulation of Brooker's merocyanine. [KKKC] = 3.34 mM, [pAA] = 4 mM, [MV] = 33.34 mM, [Brooker's merocyanine] = 0.95 µM. The scale bars are 50 µm.

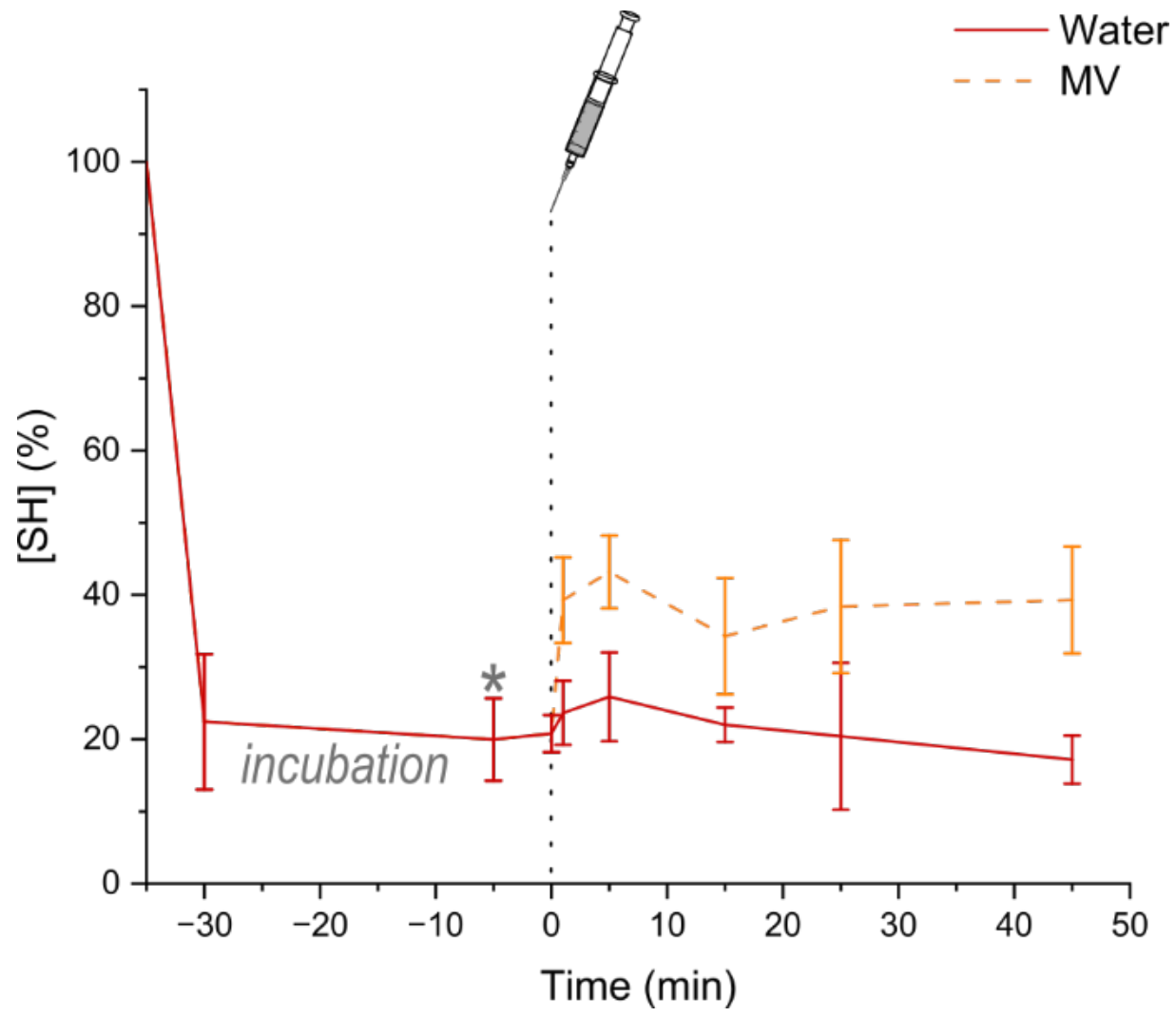

Figure S21. Regeneration of KKKC by the reaction between MV and $(KKKC)_2$ in an oxidized system. When MV was added to a coacervate sample and an electric potential was applied, the concentration of thiols (quantified by UV-Vis spectroscopy using the Ellman's assay) doubled. However, when an identical volume of water was added to the system, no increase in thiol concentration was observed. The asterisk marks the starting time of application of an electric potential (0.7 V), and the dashed line represents the time in which either water or MV were added. The reported data represent the average of triplicate experiments, and the error bars their standard deviation (n=3). [KKKC] = 3.34 mM, [pAA] = 4 mM, [MV] = 33.34 mM.

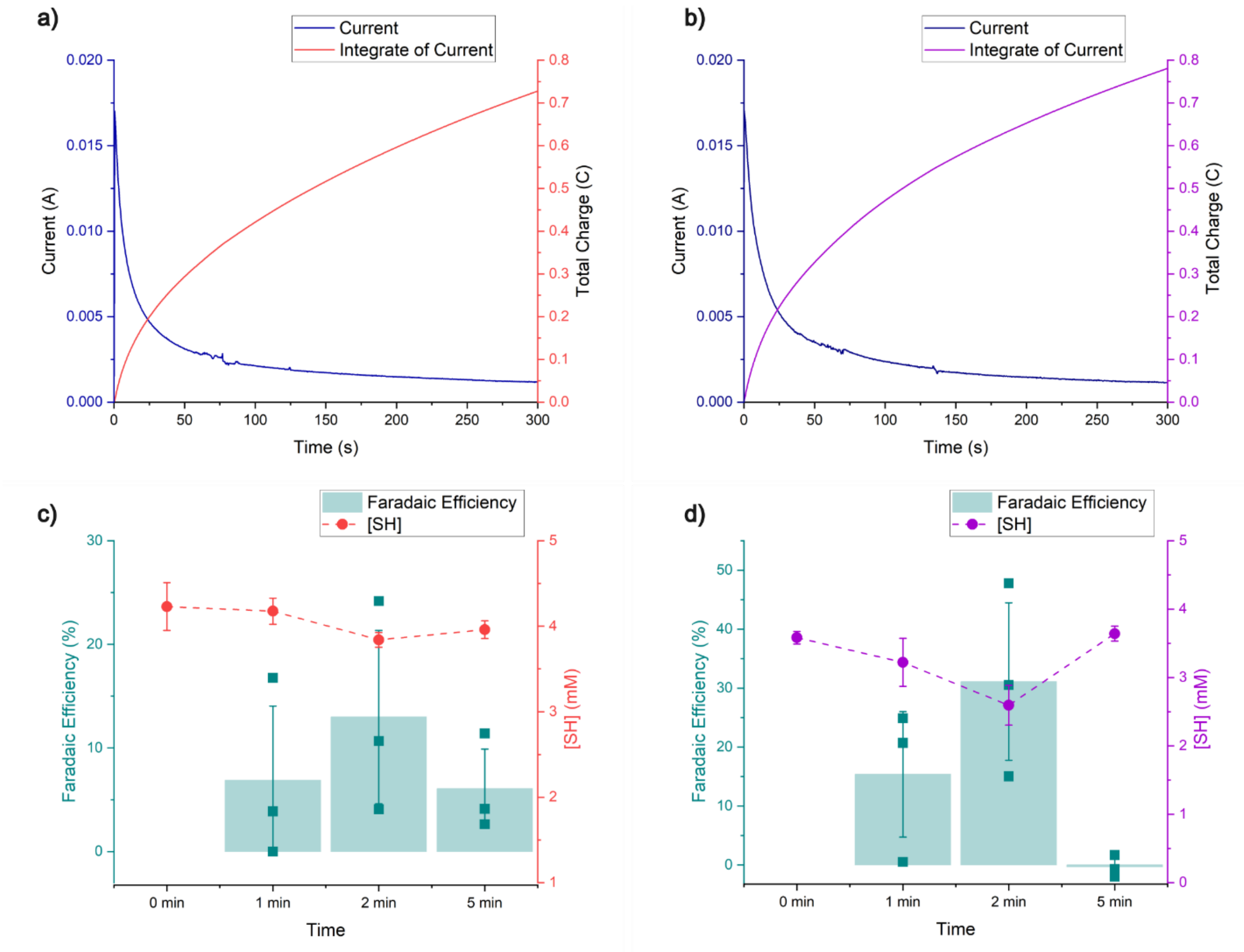

Figure S22. Chronoamperometric curve and total charge passed through the 2.5:1 KKKC:pAA system at 0.7 V a) in absence and b) in presence of MV. Faradaic efficiency of the 2.5:1 KKKC:pAA system and concentration of thiol groups of KKKC c) in absence and d) in presence of MV. The reported data correspond to the average of triplicated experiments, and the error bars to the standard deviation (n=3). [KKKC] = 3.34 mM, [pAA] = 4 mM, [MV] = 6.68 mM. Note that both the conversion and Faradaic efficiencies represented in panel d) correspond only to “observed” values, since KKKC is simultaneously being consumed by the electrochemical oxidation and regenerated by the reaction with $MV^{\cdot+}$. Therefore, the real conversion and Faradaic efficiency are higher than the values shown in this panel. This effect is particularly notable for 5-minute point, at which $MV^{\cdot+}$ has diffused through the entire cell.

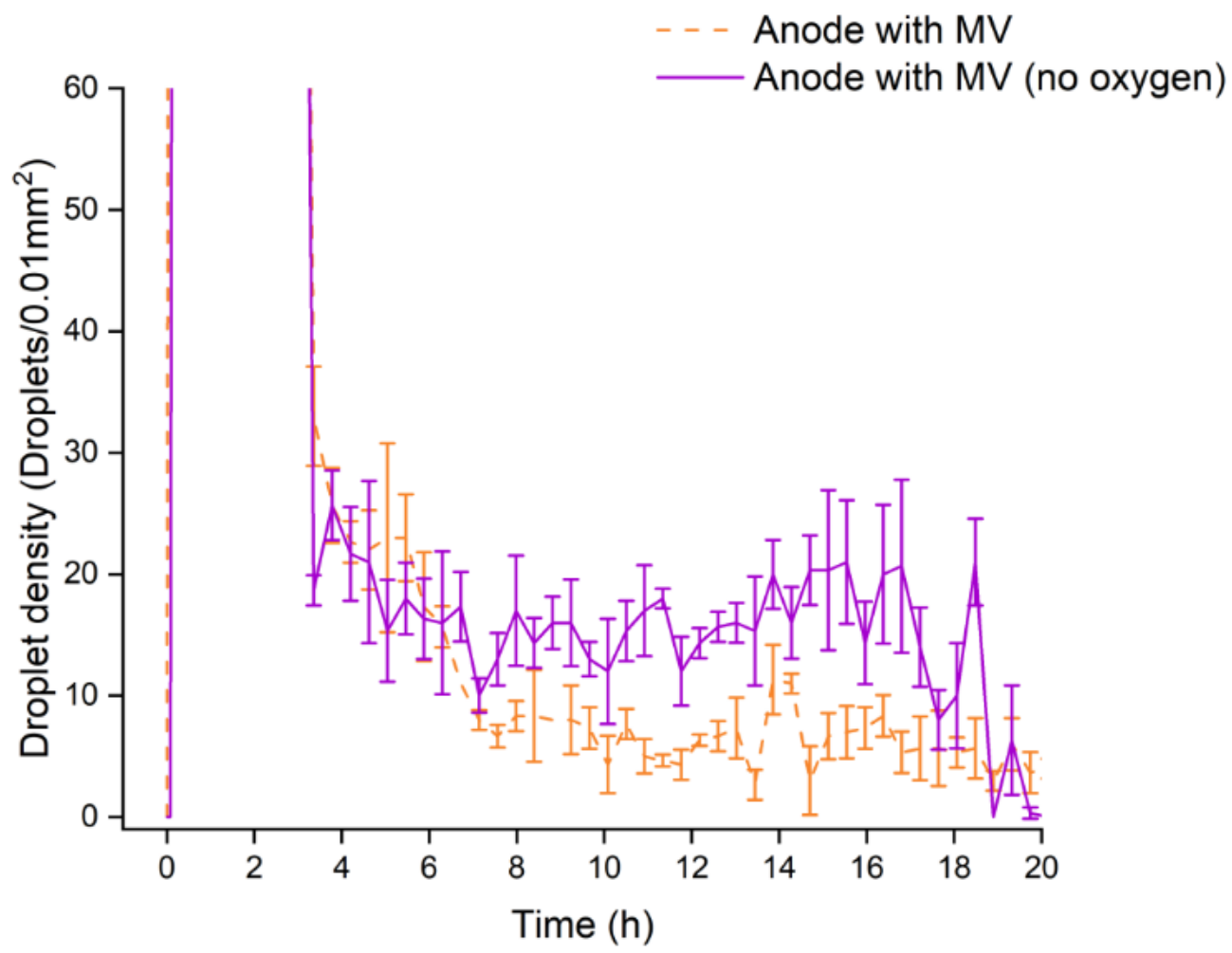

Figure S23. Effect of oxygen on the electrochemically-driven steady state. The line represents the average number of droplets per 0.01 mm^2, counted manually in separate areas in the proximity of the anode. The reported data correspond to the average of three 100 x 100 µm areas next to the surface of the electrode, and the error bars to their standard deviation (n=3). The result of two different experiments are reported here: one of them using degassed solutions and a sealed experimental setup (see Methods), and another one prepared without specific precautions to remove oxygen from the system. Both samples contained [KKKC] = 3.34 mM, [pAA] = 4 mM and [MV] = 6.68 mM. The potential applied was 0.7 V in both cases.

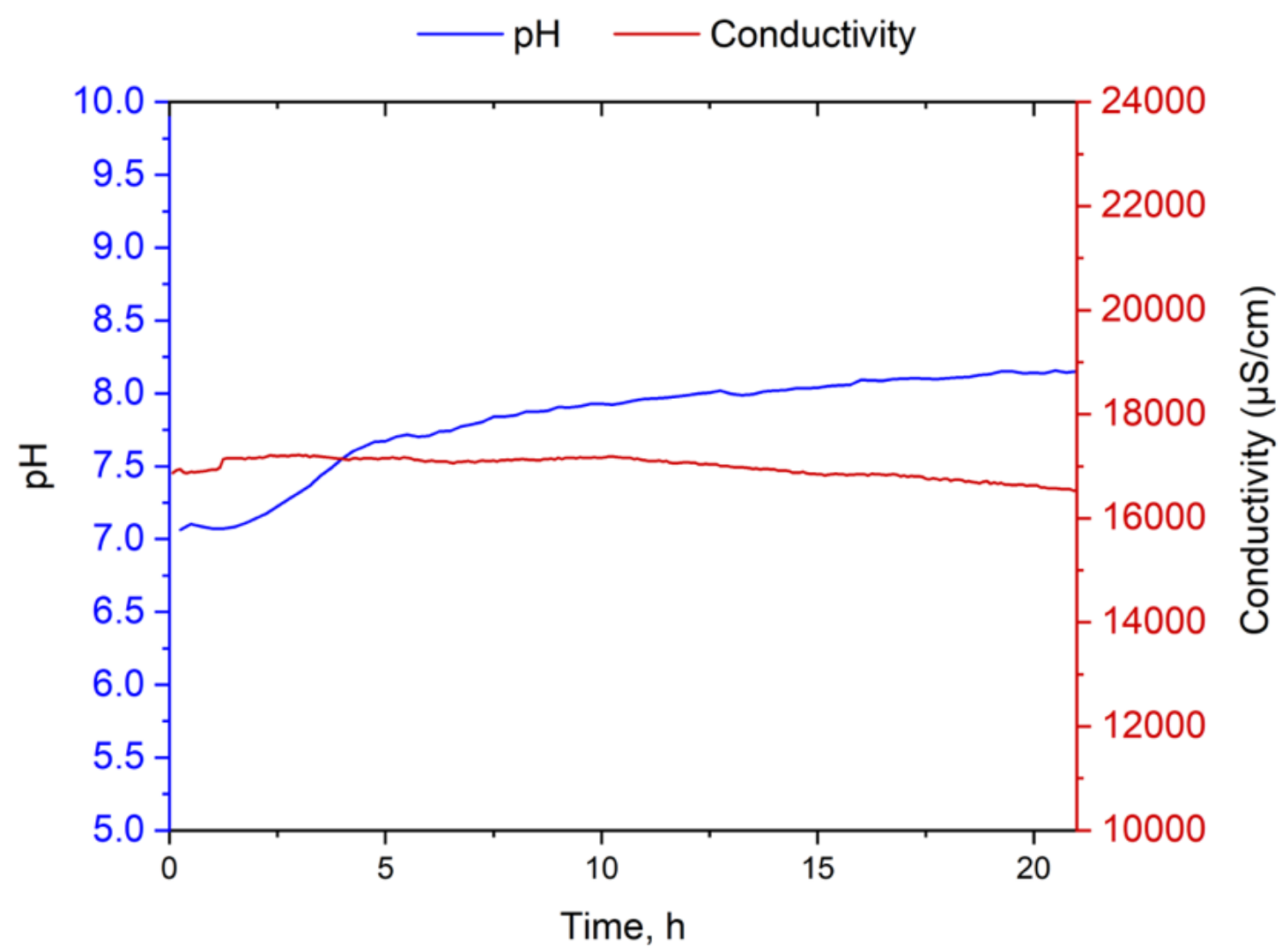

Figure S24. pH and conductivity monitored over a 21-hour electrochemical experiment (0.7 V) in a 2.5:1 KKKC/pAA system in presence of MV. [KKKC] = 3.34 mM, [pAA] = 4 mM, [MV] = 6.68 mM.

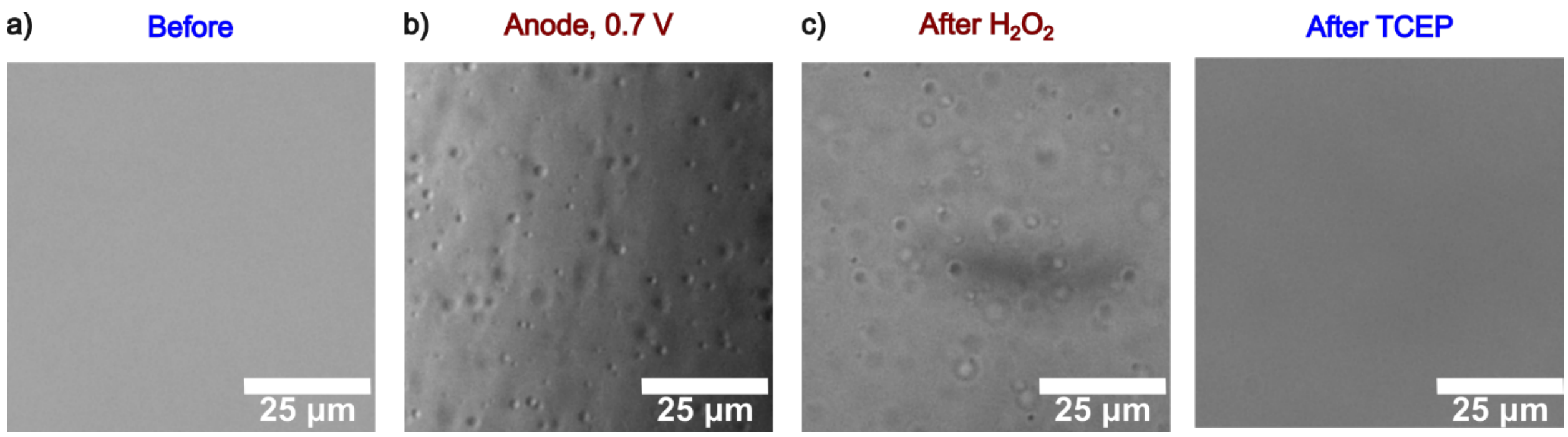

Figure S25. Electrochemical oxidation and reduction of KKKC-based coacervates prepared with pH = 8.1. Micrographs of a 2.5:1 KKKC:pAA sample with pH = 8.1 a) immediately after preparation ; b) in the proximity of the surface of the anode, 2 minutes after the application of a 0.7 V potential; c) 2 minutes after the addition of H_2O_2 (29.4 mM, 8.8 eq.) to a freshly prepared sample, and d) immediately after the addition of TCEP (32.1 mM, 9.6 eq.). [KKKC] = 3.34 mM, [pAA] = 4 mM (in monomer units). All samples contain Tris buffer (pH = 8.1, 50 mM) and NaCl (100 mM). All scale bars are 25 µm.

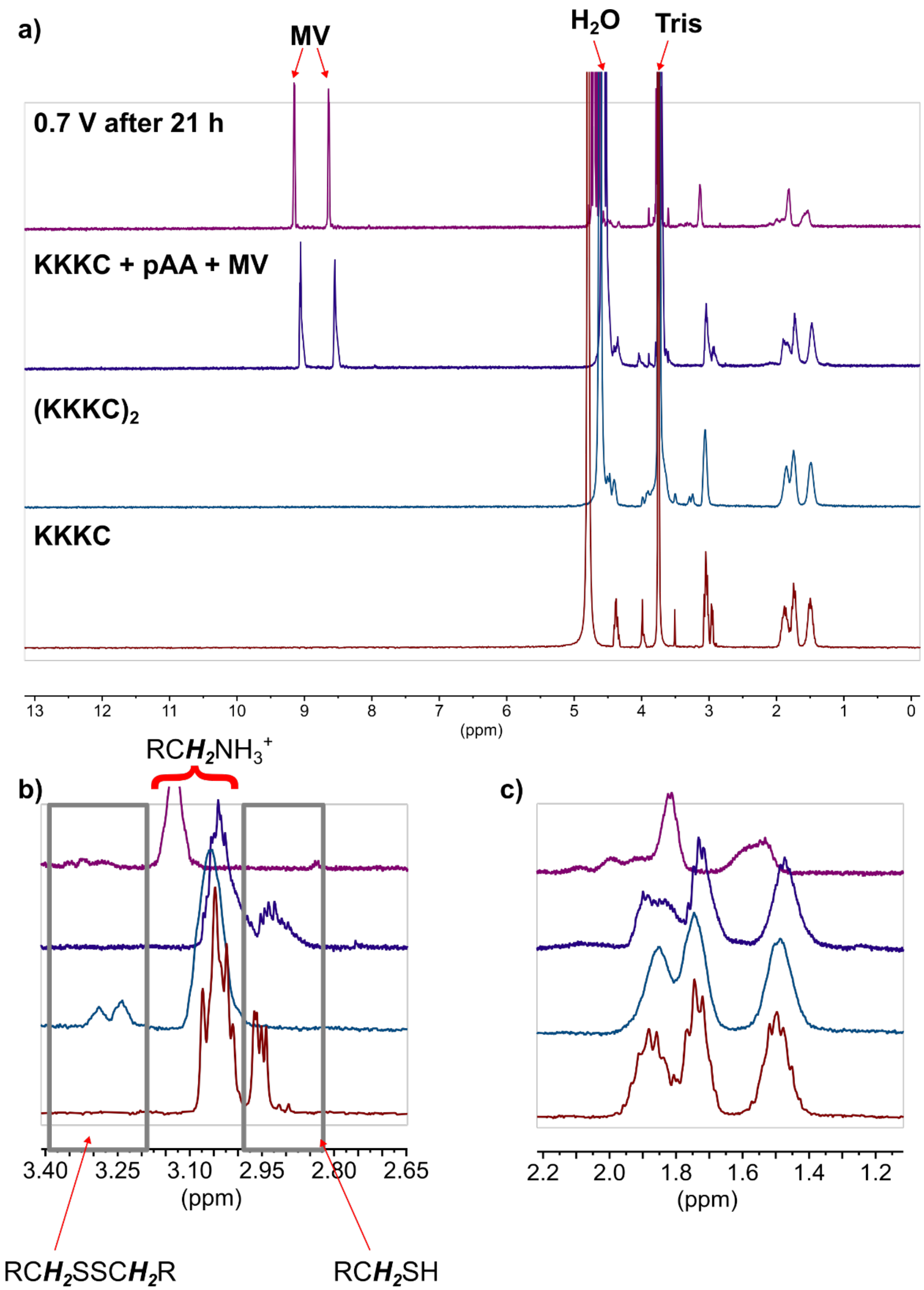

Figure S26. ^{1}H NMR spectra (300 MHz, D_2O) corresponding to a 2.5:1 KKKC/pAA mixture after continuous electrochemical cycling (0.7 V) for 21 hours in presence of MV; a freshly-prepared mixture before application of the potential, $(KKKC)_2$ obtained by electrochemical oxidation, and fresh KKKC solution. The full spectra are reported (a), as well as insets showing b) the signals corresponding to the CH_2 protons next to the thiol/disulfide groups and amino groups of the side chains of KKKC and c) the aliphatic region, where the rest of the side chain signals overlap with those coming from the backbone of pAA.

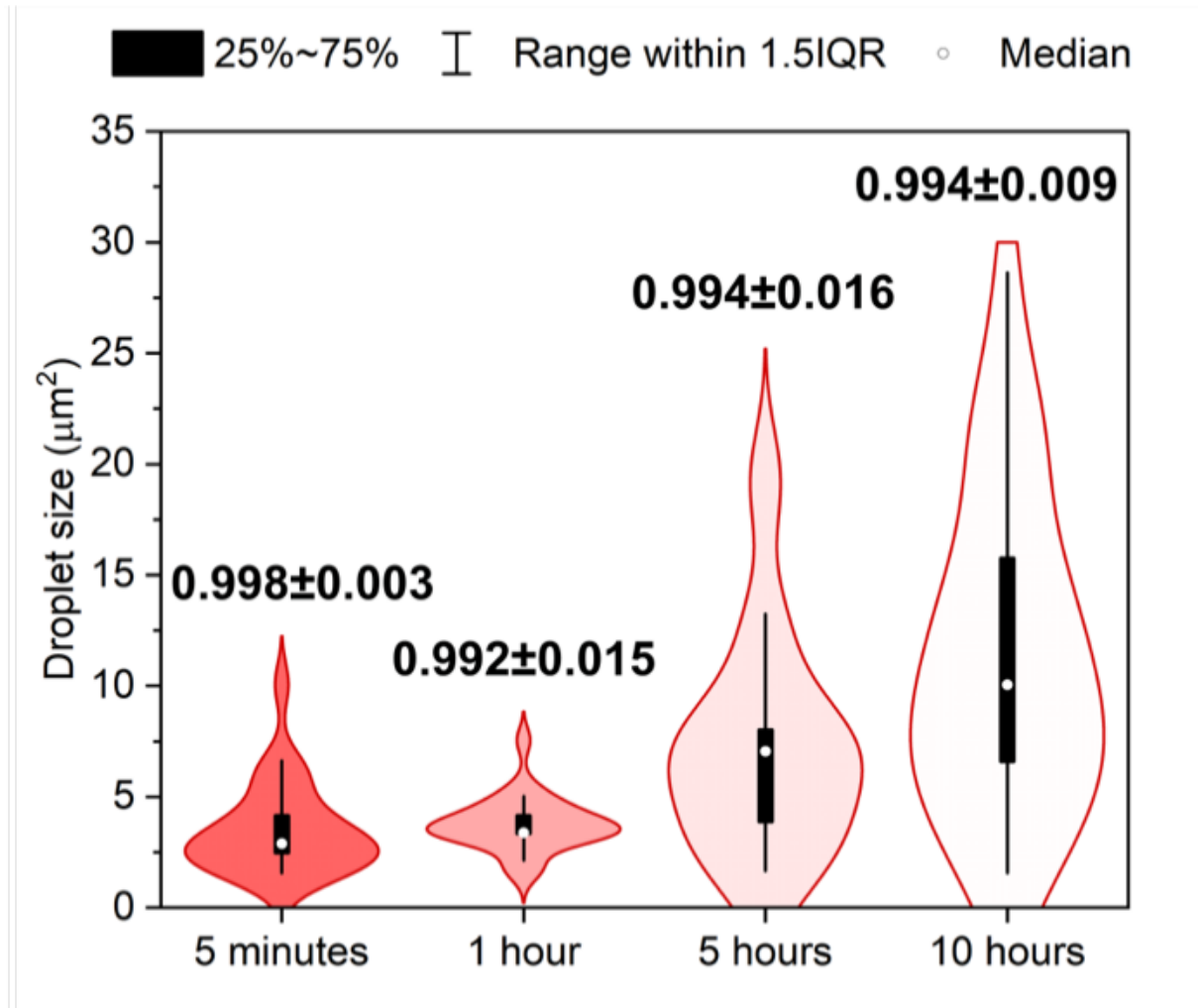

Figure S27. Size distributions of the coacervates obtained at different times during the electrochemical cycle (0.7V) in presence of MV. 25 droplets were analyzed at each time. The circularity of the droplets is reported above the corresponding violin plot (as average ± standard deviation). [KKKC] = 3.34 mM, [pAA] = 4 mM in monomer units, [MV] = 6.68 mM. The samples contained Tris buffer (50 mM, pH = 7.4) and NaCl (10 mM for ATP samples, 100 mM for pAA samples).

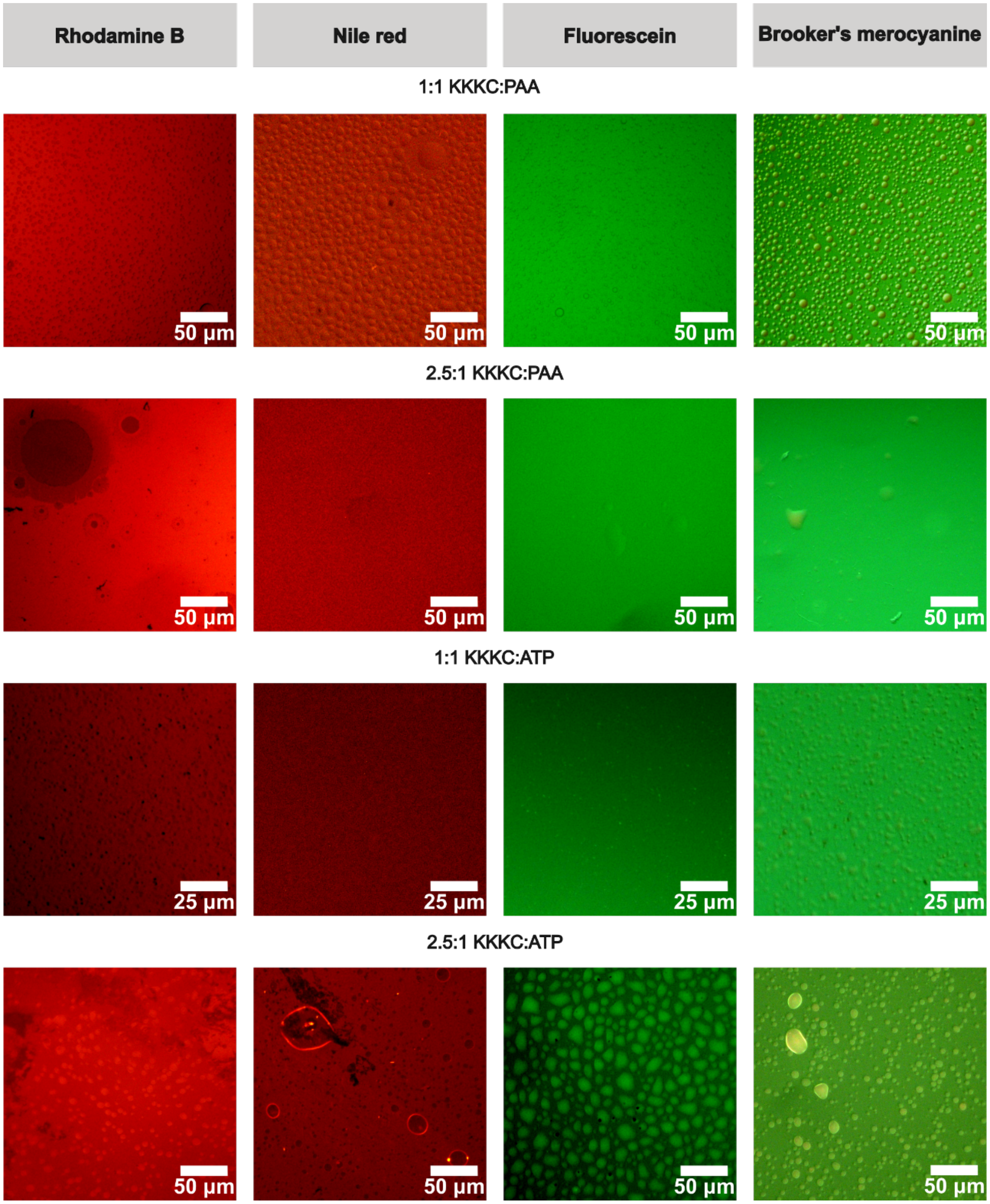

Figure S28. Fluorescence microscopy images for KKKC/pAA and KKKC/ATP coacervates in presence of neutral (Nile Red, Brooker's merocyanine), and charged (fluorescein and Rhodamine B) dyes. The partitioning of different dyes depends on the chemical composition and mixing ratio of the coacervates. All samples contained NaCl (100 mM for pAA- or 10 mM for ATP-based coacervates) and Tris buffer (50 mM). Coacervates were generated by electrochemical oxidation (0.7 V) of a sample with [KKKC] = 1.34 mM or 3.34 mM, [pAA] = 4 mM and [ATP] = 1.34 mM in monomer units. The concentration of all dyes was 0.95 µM. Dyes and coacervates were incubated overnight before imaging.

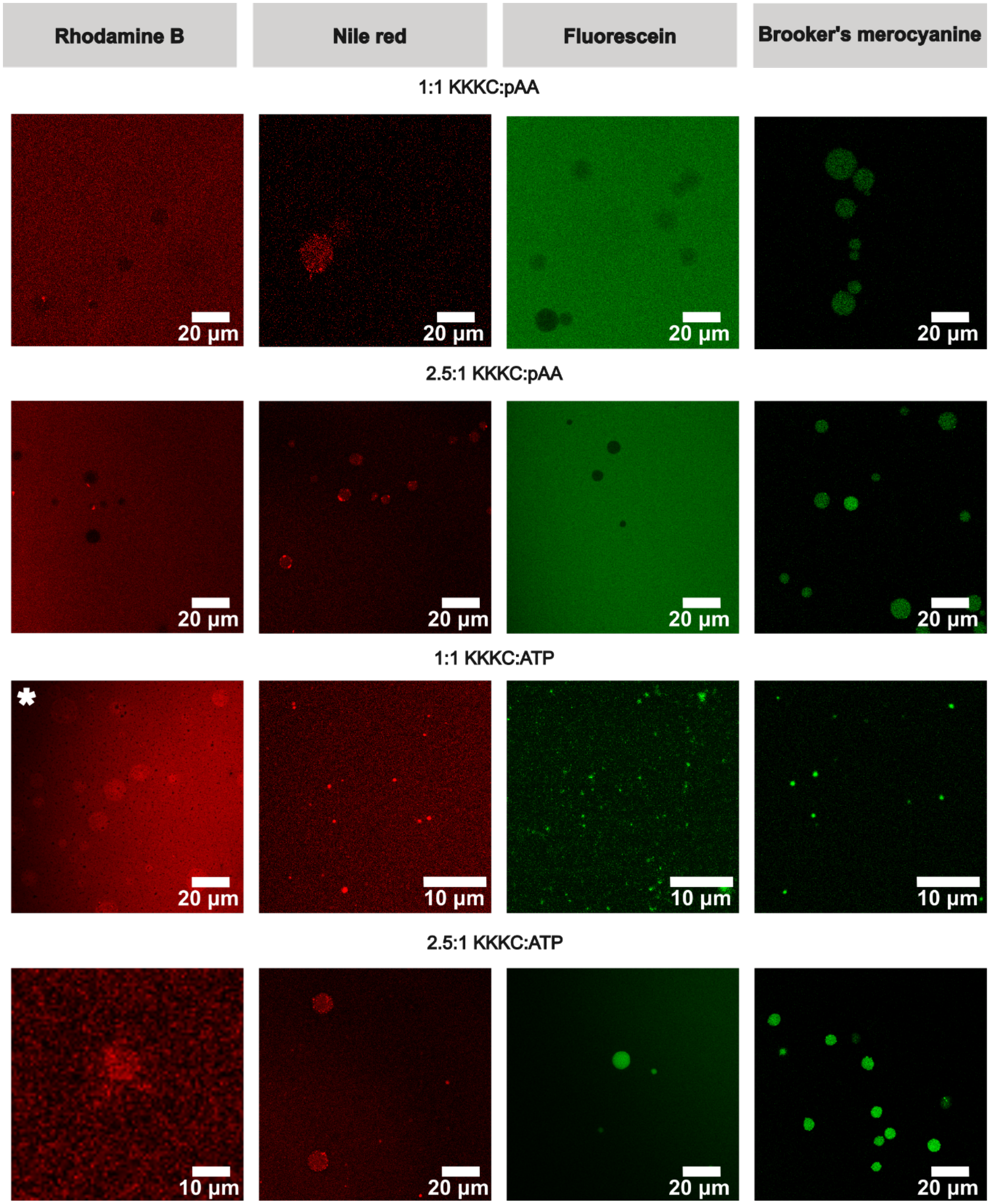

Figure S29. Confocal microscopy images for KKKC/pAA and KKKC/ATP coacervates in presence of neutral (Nile Red, Brooker's merocyanine), and charged (fluorescein and Rhodamine B) dyes. All samples except for 1:1 KKKC:ATP with Rhodamine B were prepared in the same conditions and with the same concentrations as those in Figure S28, followed by deposition on a PVA-coated slide and incubation for one (for pAA coacervates) or two (for ATP coacervates) days before imaging. For the 1:1 KKKC:ATP sample containing Rhodamine B (marked with an asterisk), the concentrations of KKKC, ATP, and dye were doubled to facilitate imaging.

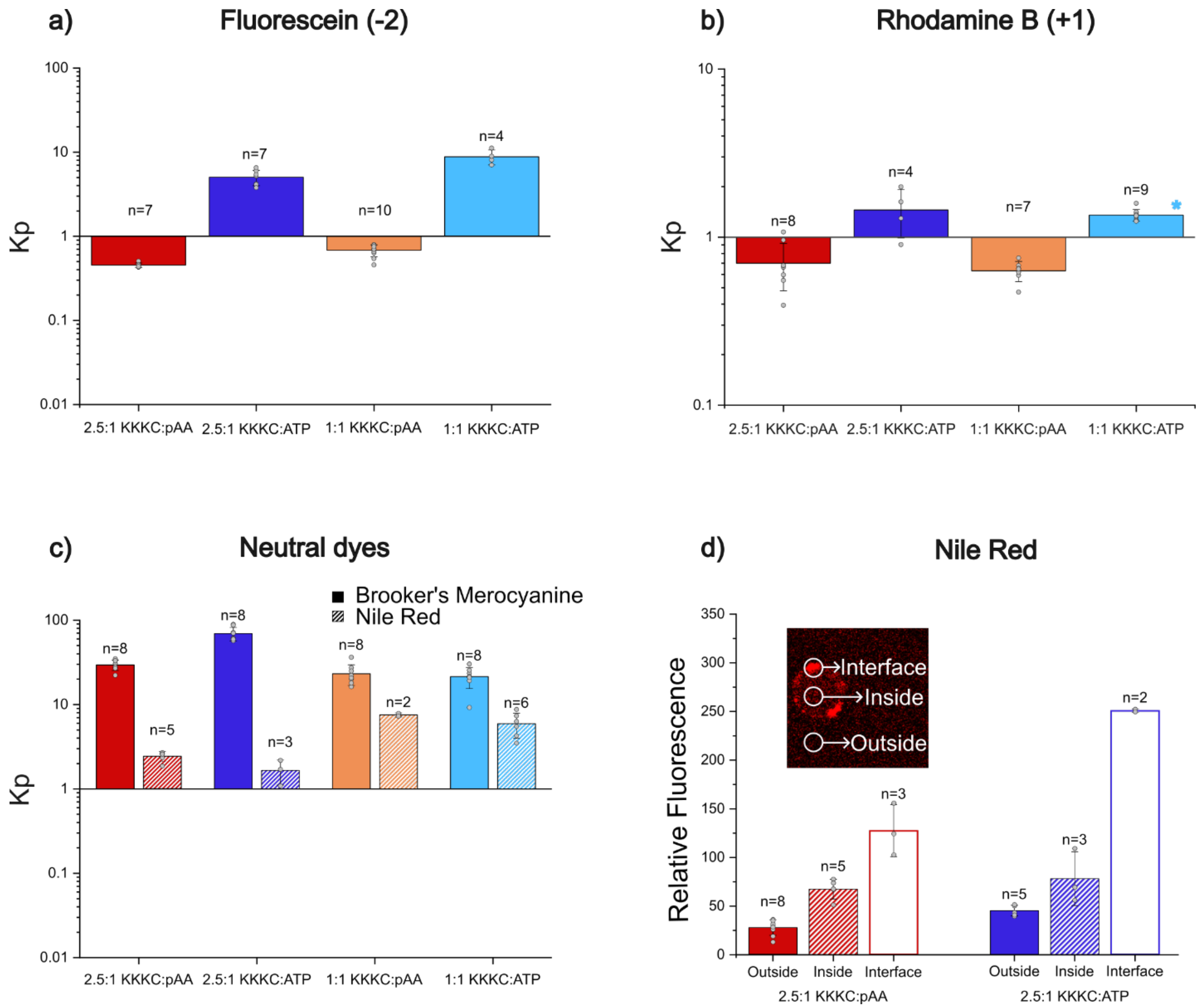

Figure S30. Partition coefficient (*Kp*) of a) a negatively-charged dye (Fluorescein), b) a positively charged dye (Rhodamine B) and c) two neutral dyes (Brooker's merocyanine and Nile Red), calculated from the confocal images on Figure S29. d) Relative fluorescence intensity of Nile Red outside, inside and on the border of the coacervate droplets, illustrating the higher accumulation of the dye molecules in the interface. The reported values correspond to the average of 2 to 9 samples (indicated above the bar) and the error bars represent the standard deviation. The asterisk in the 1:1 KKKC:ATP sample for Rhodamine B indicates that the concentration of polyelectrolytes and dye was doubled in this sample (see Figure S29)

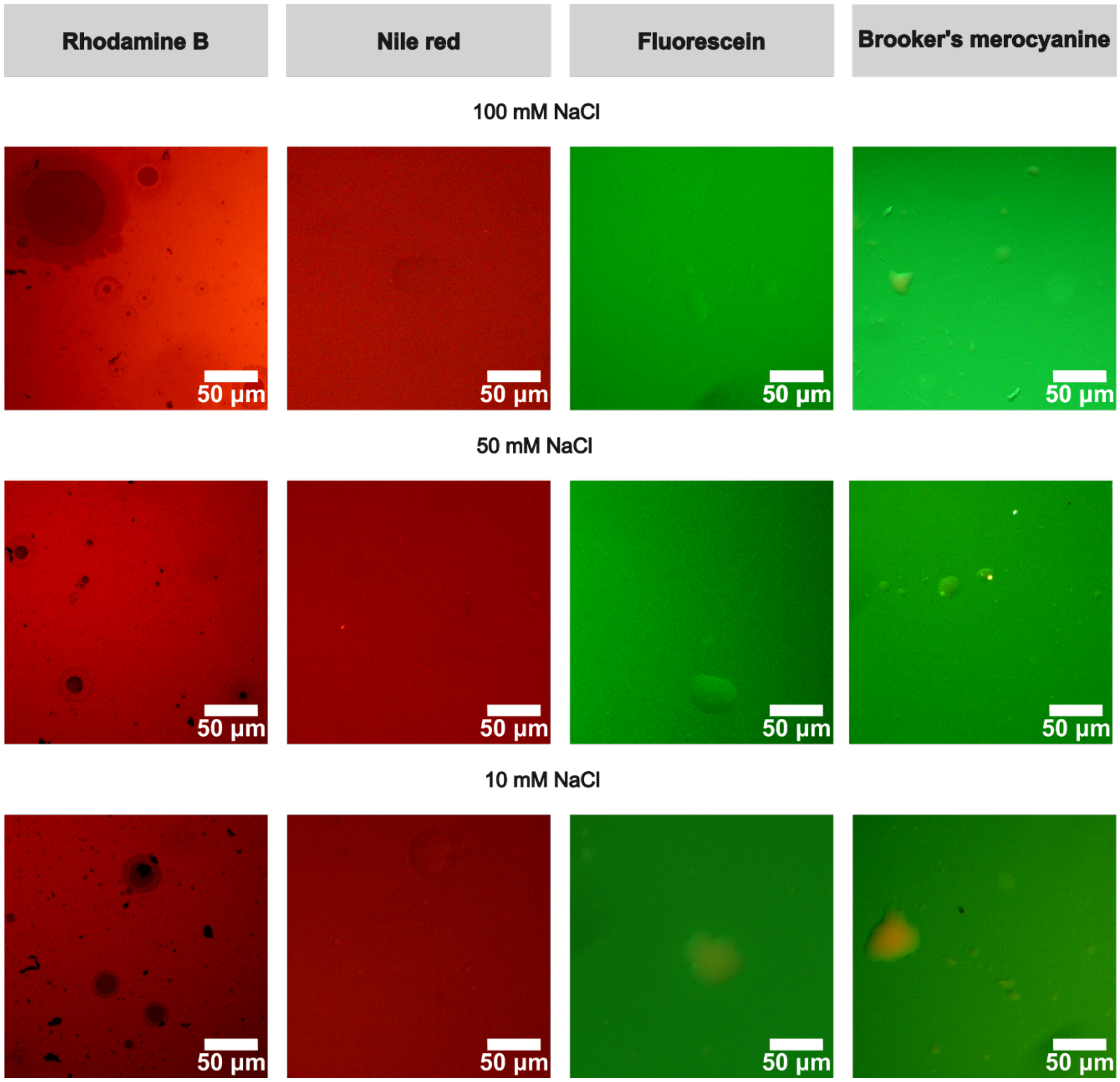

Figure S31. Fluorescence microscopy images of different dyes into 2.5:1 KKKC:pAA coacervates at different salt concentrations. While the encapsulation of Rhodamine B and Nile red is not affected by the concentration of NaCl, the partition of fluoresceine and Brooker's merocyanine into coacervate droplets increases as the ionic strength of the mixture decreases. All samples contained Tris buffer (50 mM). Coacervates were generated by electrochemical oxidation of a sample with [KKKC] = 3.34 mM and [pAA] = 4 mM in monomer units. The concentration of all dyes was 0.95 µM. Dyes and coacervates were incubated overnight before imaging.

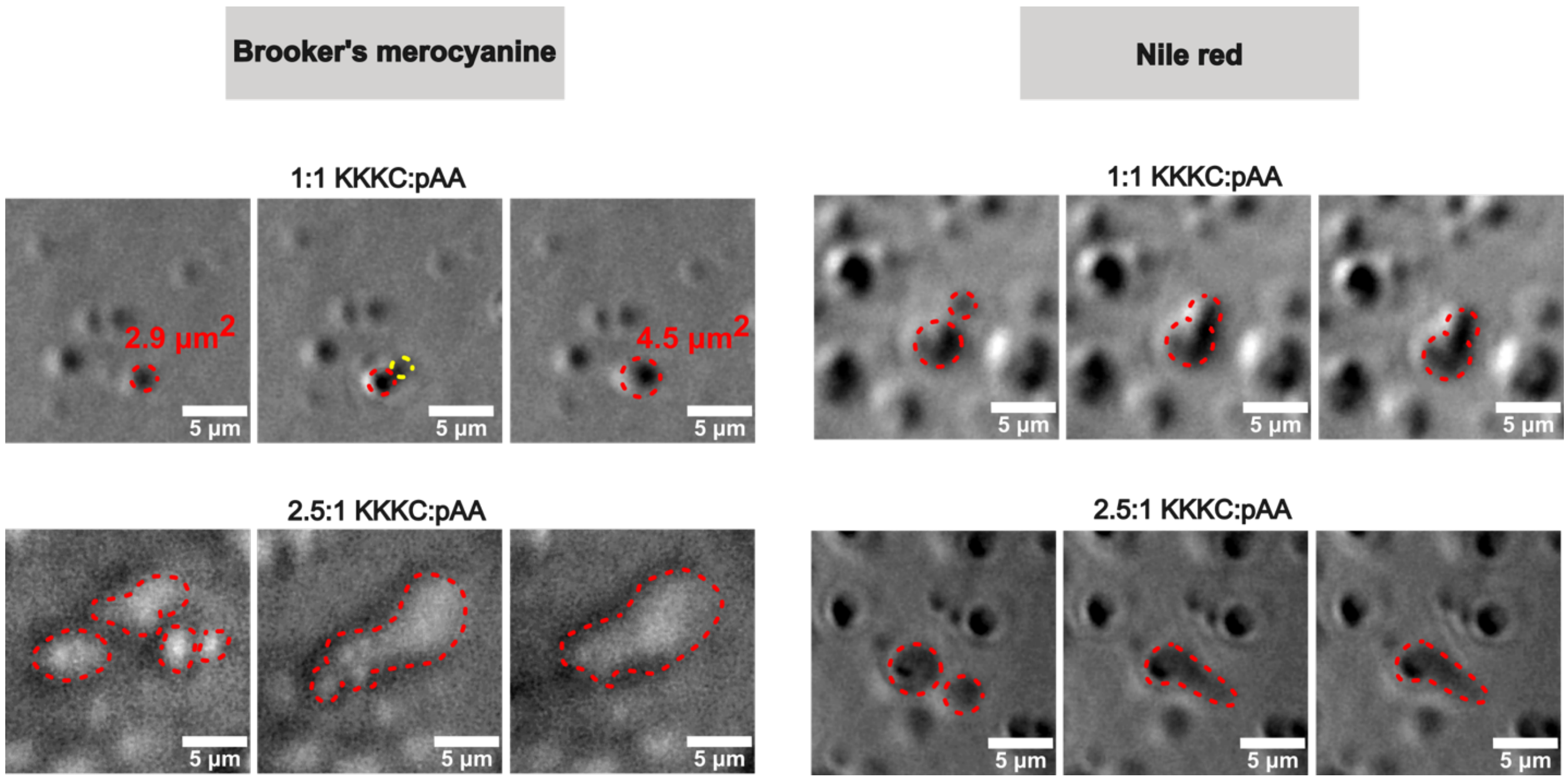

Figure S32. Merging of pAA-based coacervates containing Brooker's merocyanine and Nile Red dyes. In all cases, the three micrographs represent a series of frames separated by 2 minutes. The merging droplets have been outlined to aid visualization. The coacervates were prepared by mixing $(KKKC)_2$ with a solution containing pAA and the corresponding dye. [KKKC] = 1.34 mM or 3.34 mM, [pAA] = 4 mM in monomer units. The concentration of the dyes was 0.95 µM. The scale bars are 5 µm.

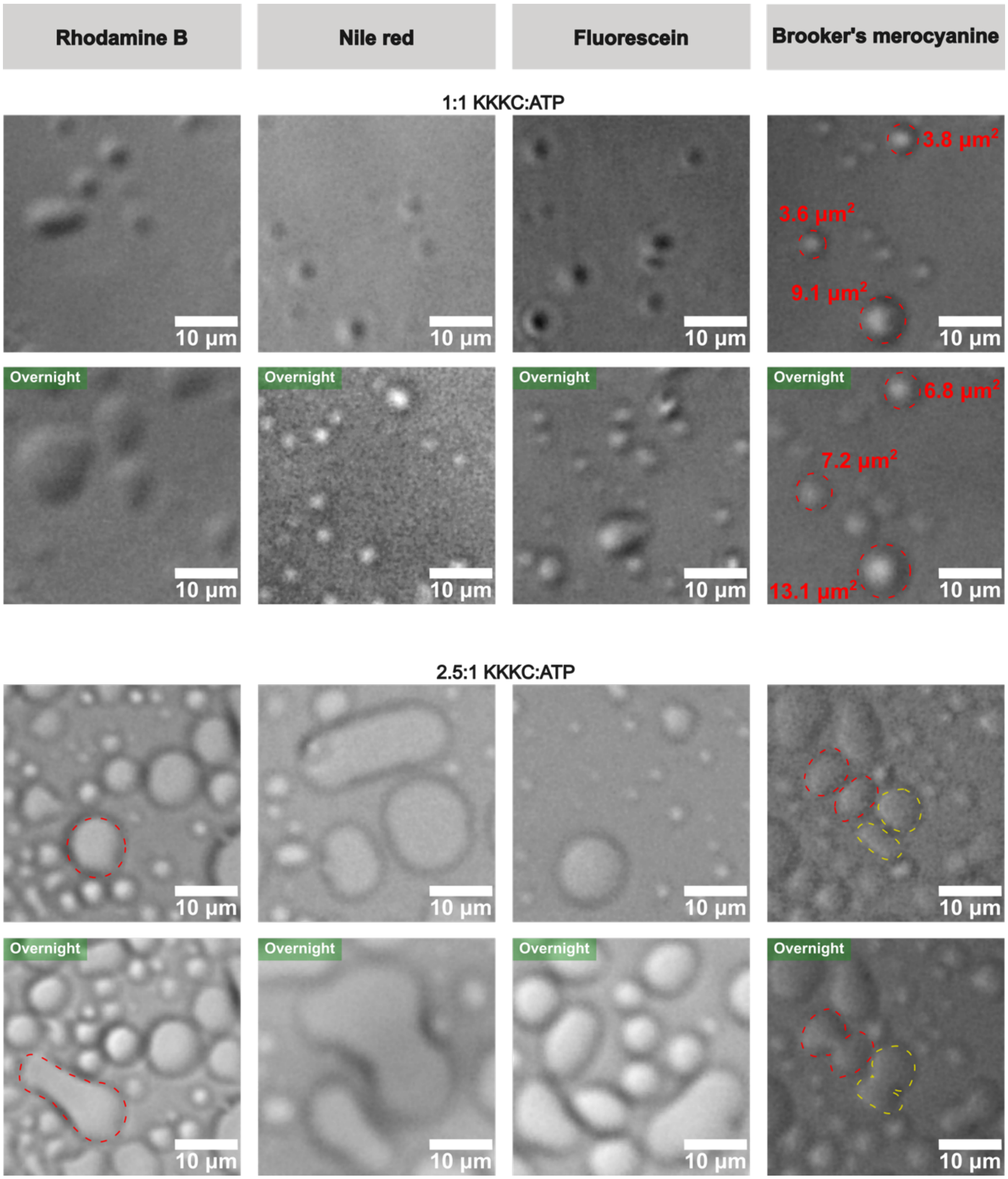

Figure S33. Merging of ATP-based coacervates containing Rhodamine B, Nile Red, Fluorescein and Brooker's merocyanine. The coacervates were obtained via electrochemical oxidation of 1:1 and 2.5:1 KKKC:ATP mixtures (0.7 V) and incubated with the dyes overnight. For all the samples the micrographs were recorded at exactly the same positions immediately after deposition and overnight. Some droplets have been outlined to aid with visualization. [KKKC] = 1.34 mM or 3.34 mM, [ATP] = 1.34 mM. The concentration of all dyes was 0.95 µM. The scale bars are 10 µm.

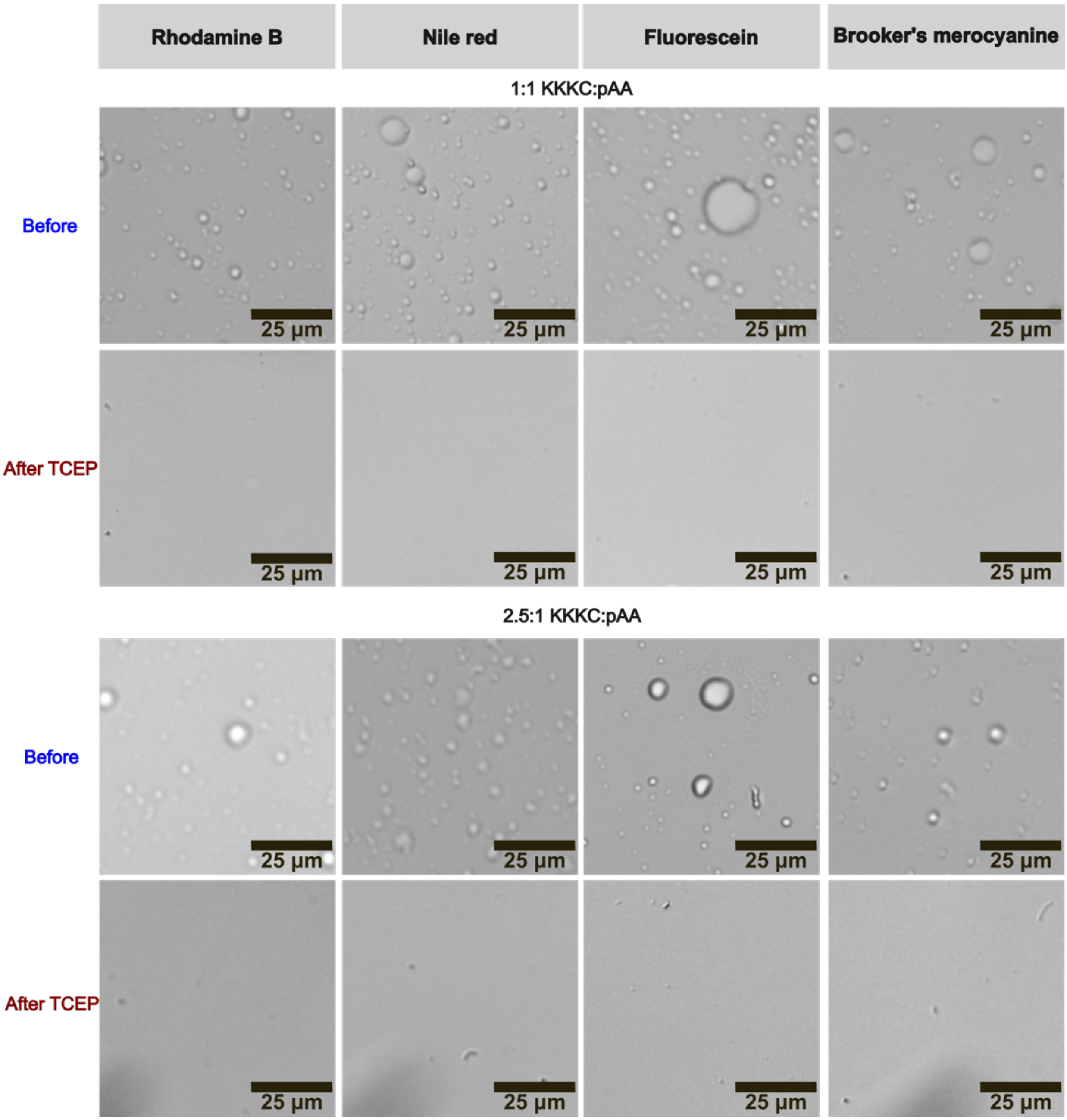

Figure S34. Dissolution of pAA-based coacervates in presence of different dyes upon addition of TCEP (19.6 mM). The coacervates were prepared via electrochemical oxidation of 1:1 and 2.5:1 KKKC:pAA mixtures (0.7 V) and incubated with Rhodamine B, Nile Red, Fluorescein or Brooker's merocyanine overnight. The micrographs before and after TCEP addition were recorded in the same positions of the well-plate. [KKKC] = 1.34 mM or 3.34 mM, [pAA] = 4 mM in monomer units. The concentration of all dyes was 0.95 µM. The scale bars are 25 µm.

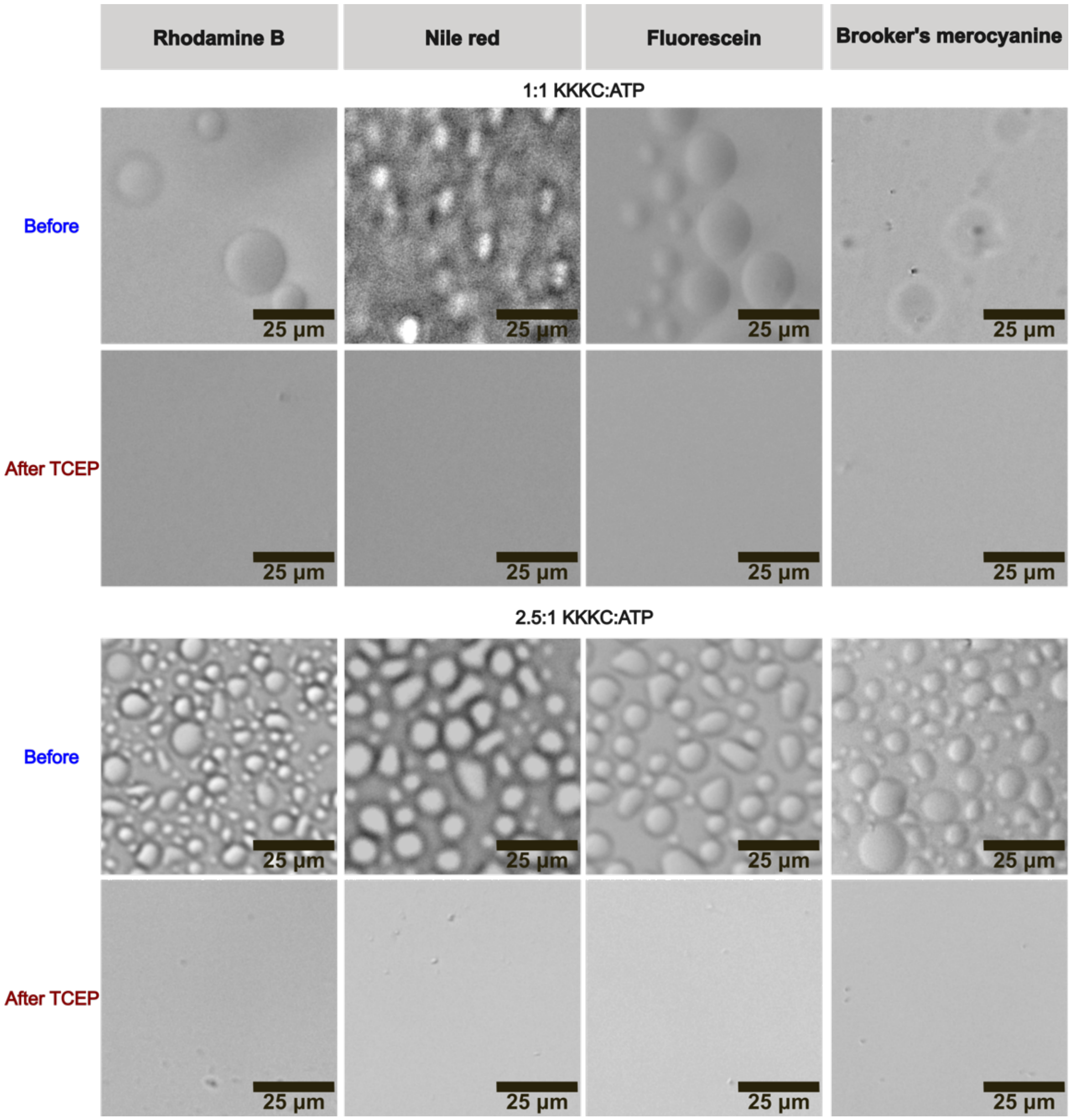

Figure S35. Dissolution of ATP-based coacervates in presence of different dyes upon addition of TCEP (19.6 mM). The coacervates were prepared via electrochemical oxidation of 1:1 and 2.5:1 KKKC:ATP mixtures (0.7 V) and incubated with Rhodamine B, Nile Red, Fluorescein and Brooker's merocyanine overnight. The micrographs before and after TCEP addition were recorded in the same positions of the well-plate. For 1:1 KKKC:ATP the concentrations of the polyelectrolytes were: [KKKC] = 3.34 mM, [ATP] = 3.34 mM and the concentration of all dyes was 1.8 µM. For 2.5:1 KKKC:ATP the concentrations of the polyelectrolytes were: [KKKC] = 3.34 mM, [ATP] = 1.34 mM and the concentration of all dyes was 0.95 µM. The scale bars are 25 µm.
