# Supplementary material for: Sustained formation movement and destruction of coacervates in a waste-free system driven by electricity
Source: Nat Commun. 2026 Aug 27;17:10245. doi: 10.1038/s41467-026-77229-1 (PMC13616953; doi:10.1038/s41467-026-77229-1)
Supplement: Supplementary file 2 [file 41467_2026_77229_MOESM2_ESM.pdf]

Description of Additional Supplementary Files

Supplementary Video 1, showing the real-time electrophoretic movement of coacervates with different compositions (MP4);

Supplementary Video 2, showing time-lapses recorded next to the anode and cathode for samples with and without MV (MP4).

Supplementary Video 3, showing a comparison of zoomed-in regions next to the anode and cathode of samples with and without MV, over the course of 16 h (MP4)

Supplementary Video 4, showing that the addition of MV allows the regeneration of coacervates in a sample where they had been depleted from the anode. (MP4)

Supplementary Video 5, showing a time-lapse recorded next to the anode in an oxygen-free sample containing MV (MP4)
