# Supplementary material for: Sustained formation movement and destruction of coacervates in a waste-free system driven by electricity
Source: Nat Commun. 2026 Aug 27;17:10245. doi: 10.1038/s41467-026-77229-1 (PMC13616953; doi:10.1038/s41467-026-77229-1)
Supplement: Supplementary file 3 [file 41467_2026_77229_MOESM8_ESM.pdf]

nature portfolio

Peer Review File

Sustained Formation, Movement and Destruction of Coacervates in a Waste-Free System Driven by Electricity

Corresponding Author: Dr Guillermo Monreal Santiago

This file contains all reviewer reports in order by version, followed by all author rebuttals in order by version.

Attachments originally included by the reviewers as part of their assessment can be found at the end of this file.

Reviewer 2 also reviewed the rebuttal to Reviewer 1.

Version 0:

Reviewer comments:

Reviewer #1

(Remarks to the Author)
In this study, Samokhvalova and coworkers studied a unique system of coacervate, formed by the oxidation of a cysteine-containing polycationic peptide and a polyanion. By establishing the electrochemical oxidation of this cysteine-containing peptide, the authors are able to form coacervates by applying an electric field, rather than the conventional method of adding oxidizing agents. The authors then studied the motion of these droplets due to the electric field. The authors then went ahead to study the dissolution of these droplets by reduction of the cysteine-containing polycationic peptide, mediated by a reducing agent that is processed electrocatalytically, and studied the cyclic evolution of this process at steady state.
This steady state evolution of destruction and regeneration of droplets is not clear to me. While it is clearly visible that, in samples with MV present, there is a reduction in the size of the droplets near the cathode (Fig. 4c), the subsequent regeneration of droplets near the anode is not at all clear. Furthermore, the control videos are trimmed off at 6 or 8 hours, so one is unable to see if there are/aren't droplets at later times.
a. It would be useful to have the four videos, anode and cathode, with and without MV, displayed together in a single panel with synchronized time for the reader to appreciate the contrast between the two conditions.
b. A simple yet direct proof of regeneration would be to take the control system (without MV), allow it to achieve a steady state so that no droplets are present at the anode, and then add MV to show regeneration of droplets. A control study, where water without MV is added, must supplement this proof experiment to account for any mixing/convection flow of existing droplets.

While the concept and results are unique, the study is quite incomplete, and the manuscript does not contribute significantly to the understanding of these new systems.

Some other comments
1. In the caption of Figure 1, MV is not defined. After going through the manuscript, it became clear that it refers to Methyl Viologen. Please define it at the first occurrence for clarity.
2. On page 3, the authors note that the KKKC/ATP system forms droplets under an electric field, while (KKKC)2/ATP did not form droplets in the absence of an electric field. Could this be because of the pKa shift of ATP in the presence of an applied electric field? Many studies, including this recent one (https://pubs.acs.org/doi/10.1021/jacs.3c13633), have shown that applied potential tunes protonation state at fixed bulk pH, implying significant shifts in pKa driven by local interfacial electric fields. Thus, it is possible that ATP3- is converting to ATP4- under an electric field, allowing complexation with KKKC2. Could the authors comment on this possibility? Perhaps some measurements can be done to know if this pKa/pH change is indeed leading to coacervation in this system by 1) checking if ATP4- (at pH > 8) can complex with KKKC2 in the absence of an e-field, and 2) adding borate salts to the droplets formed under an e-field to check their salt resistance.
3. To prevent the wetting of the glass surface by coacervate droplets, surface coatings such as BSA, PEG, silane, or PVP can be employed. An earlier paper studying coacervate droplets under an electric field did such coating to prevent droplet wetting (https://doi.org/10.1073/pnas.2203483119). This may allow observation and circulation of droplets for an extended period of time.

Reviewer #2

(Remarks to the Author)
I co-reviewed this manuscript with one of the reviewers who provided the listed reports. This is part of the Nature Communications initiative to facilitate training in peer review and to provide appropriate recognition for Early Career Researchers who co-review manuscripts.

Reviewer #3

(Remarks to the Author)
Designing active coacervate droplets has garnered significant interest for mimicking biological cellular compartments, particularly focusing on the regular creation of coacervates out of equilibrium. In this work, the authors propose an electricity-driven system for the transient formation, directional motion, and dissolution of complex coacervates. The design involves coupling a cysteine-containing peptide through electrochemical oxidation, with the double multivalency triggering ionic complexation with a polyanion, leading to coacervate formation at the anode. The resulting droplets migrate electrophoretically over a relatively long distance toward the cathode, where the electrocatalytic cycle dissolves the coacervates through reduction of the peptide. The free peptide and polyanion are then recovered, diffuse back to the anode, and react again, establishing a steady state in which droplets continuously form, move, and disappear in different regions of the cell. This steady cycling arises from the prevention of side product generation during the electrocatalytic process. The authors also demonstrate that the electrochemically formed droplets can spontaneously sequester solutes from the surrounding solution, enabling potential applications in electrically driven cargo transport and gradient formation. While the work generally validates the transient formation and movement of coacervate droplets powered by electricity, several important concerns remain regarding a deeper analysis of the coacervation process and further clarification of the related statements. These issues, outlined below, need to be thoroughly addressed before the study can be considered for publication in Nature Communications.
1.In the title, the authors refer to the system as a “Waste-Less System,” which does not seem to align well with the statement in the abstract that claims “no side-product.” Does the cycle completely eliminate side products? What potential by-products could arise during the electrocatalytic process? Further clarification on this point is recommended, including additional analysis of the system before and after recycling. For instance, an analysis of the pH and ionic strength of the solution, as well as qualitative and quantitative analysis using 1H NMR spectra to identify chemicals and their concentrations, would help to better understand any possible side products.
2.The control factors for phase separation are not sufficiently investigated in the first part of the study. The authors primarily rely on qualitative optical microscopy to illustrate coacervate formation. A more comprehensive investigation of the control factors (such as mixing ratio and ionic strength) using quantitative analysis, such as turbidity measurements and fluorescence imaging of the droplets, is recommended to fully define the phase separation in the system. Similarly, the dissociation of the coacervates could also be further clarified through quantitative analysis.
3.General capability of the proposed strategy: According to Figure 1, the key feature of the peptide design is the terminal thiol group of KKKC, which can be oxidized to double the charge and trigger phase separation. Do the other sequences in the peptide also play a role in regulating phase separation? What would happen if the terminal thiol group is retained but the peptide sequence is varied?
4.The details regarding the measurements of the coacervate droplets (such as Zeta potentials, size, morphology, etc.) in this dynamic and flowing system need to be supplemented.
5.Page 6, “The intensity of the color was significantly stronger within the coacervates than in the surrounding solution, indicating that the diffusion of MV·+ into the coacervate phase is a favorable process.” The authors are suggested to provide more quantitative analysis, for example, the partition coefficient of MV·+ to support this claim. Additionally, does the flow affect the partition coefficient compared to conventional coacervation?
6.The final part regarding the incubation of different fluorescent dyes by coacervates requires further study, as it serves as a key proof supporting the potential application of the proposed coacervate regulation. Ideally, the dye molecules should be classified into different categories, and the partitioning should be analyzed using both qualitative and quantitative methods to explore the principles behind selective encapsulation.
7.The quality of the images and figures could be improved in terms of both aesthetics and clarity.

Reviewer #4

(Remarks to the Author)
In this manuscript, authors have presented the relatively new approach to make coacervates through oxidation of cysteine amino acids in the design of tetrapeptide. This approach brings the way forward to avoid the formation of side-products during the coacervation process of out-of-equilibrium droplets, using electrocatalytically mediated oxidation and reduction. Authors have claimed the movement of coacervates, self-destruction and reformation within the same system. Overall, it is interesting study, and I'd recommend authors to improve the manuscript with following comments.
1. KKKC can have 3+ charges while pAA is a polymer and I'm wondering how the charges are balanced in final product (coacervates) and how exactly the deprotonation happens to bring the negative charges on pAA motif?
2. To make this more versatile, other oxidizing agents such as TCEP, DTT should be tried as well.
3. Movement of droplets is interesting, and I'd be more curious about the role of sodium and chloride ions and also counter ions from the salt of polymer. Do they play a crucial role for movement?
4. In figure 4, MV is supporting the reduction of peptide to resultantly destroy the droplets, after destruction is the reformation of coacervate time remain same or it is changed? can MV slow down the destruction through possibly cation-pi interactions?
5. In figure 4c, after 1 minute the blue color indicates the MV but droplets can be still seen in the image, would be great to detailed response why still coacervates remain unchanged.
6.In Figure S13, different dyes have been used to encapsulate inside coacervate droplets, authors should show partition

coefficient of each dye and how these dyes effect the movement and destruction/reformation of coacervates. Authors should also show the impact of these dyes on fluidity of coacervate droplets maybe FRAP could help to investigate this.

Version 1:

Reviewer comments:

Reviewer #1

(Remarks to the Author)
The study by Samokhvalova and coworkers is an ambitious one where they explored a system of coacervate that forms and gets destroyed by cyclic redox in response to electric potential.
After addressing several previous comments from reviewers, the authors have amended the article and the supporting information with additional studies and clarifications, which adds to the coherence and boldness of reported findings. It should also be noted that the language and nature of presentation and explanations of various findings are uncomplicated. However, some shortcoming are apparent. s
The electrodes and their potential conundrum remain despite additional study and new findings. Since the silver is playing a role, replacing those with inert ones could have been a way to easily assess the potential impact on the formation of the coacervates.
Although the claim is that it is waste-free, the authors do mention the degradation of the electrodes eventually causes the system to stop working. Rather than a qualitative assessment, a quantitative study would reveal if that were the case in actuality and also aid in revealing the potential role of silver ions.
As the authors mention of competing redox reactions as a potential inhibitor to the continuity of the formation, movement and destruction, some simple steps to prevent such could have been taken, such as removal of oxygen to show if the presence of such contaminant is actually the reason behind it.
Although qualitative assessment of MV is reported, quantitative assessment would strengthen the claim of MV being the electrocatalytic mediator. Correlating total charge passed with the amount of disulfide formed would allow estimation of Faradaic efficiency and clarify whether competing side reactions significantly contribute.

Reviewer #2

(Remarks to the Author)
I co-reviewed this manuscript with one of the reviewers who provided the listed reports. This is part of the Nature Communications initiative to facilitate training in peer review and to provide appropriate recognition for Early Career Researchers who co-review manuscripts.

Reviewer #3

(Remarks to the Author)
The revision has addressed most of my previous concerns; however, one point remains as indicated in Comment 2. How did the authors determine the charge mixing ratios of 1/1 and 2.5 as the representative cases? Are there any results to support that these ratios are ideal stoichiometric and non-stoichiometric mixing charge ratios? This selection appears somewhat arbitrary. A comprehensive screening of turbidity under a broader range of charge ratios could clarify the rationale for choosing these specific ratios for investigation.

Reviewer #4

(Remarks to the Author)
The authors have adequately addressed all of my concerns. I have no further questions and recommend this manuscript for publication in Nature Communications.

Version 2:

Reviewer comments:

Reviewer #1

(Remarks to the Author)
The authors have done a commendable job in addressing concerns regarding the replacement of electrodes and the influence of oxygen in the system. However, the role of silver in the coacervation process still requires further clarification. Since silver appears to participate in or influence the complexation process, a more detailed discussion regarding its exact role would strengthen the manuscript. In particular, clarification regarding possible Ag–thiol coordination effects and Ag^{+}-induced stabilization of the coacervate phase would be valuable.

Regarding the use of the term “waste-free”, I partially agree with the authors’ justification, especially considering similar terminology has been used in related previous studies. Nevertheless, some refinement in the terminology or additional clarification could help avoid ambiguity for readers from broader electrochemical or materials backgrounds.
The Faradaic efficiency experiments provide useful insight into the role of methyl viologen (MV), and the observed trends support its involvement in the electrochemical cycle. However, the relatively low Faradaic efficiencies also raise questions regarding the fate of the remaining electrons and the possible contribution of competing electrochemical reactions- which could be addressed in subsequent studies.
Overall, revisions have considerably improved both the scientific clarity and the presentation of the work.

Reviewer #2

(Remarks to the Author)
I co-reviewed this manuscript with one of the reviewers who provided the listed reports. This is part of the Nature Communications initiative to facilitate training in peer review and to provide appropriate recognition for Early Career Researchers who co-review manuscripts.

Reviewer #3

(Remarks to the Author)

Version 3:

Reviewer comments:

Reviewer #2

(Remarks to the Author)
[Note from the editor: Please also see attached PDF]

I co-reviewed this manuscript with one of the reviewers who provided the listed reports. This is part of the Nature Communications initiative to facilitate training in peer review and to provide appropriate recognition for Early Career Researchers who co-review manuscripts.

Open Access This Peer Review File is licensed under a Creative Commons Attribution 4.0 International License, which permits use, sharing, adaptation, distribution and reproduction in any medium or format, as long as you give appropriate credit to the original author(s) and the source, provide a link to the Creative Commons license, and indicate if changes were made.
In cases where reviewers are anonymous, credit should be given to 'Anonymous Referee' and the source.
The images or other third party material in this Peer Review File are included in the article’s Creative Commons license, unless indicated otherwise in a credit line to the material. If material is not included in the article’s Creative Commons license and your intended use is not permitted by statutory regulation or exceeds the permitted use, you will need to obtain

permission directly from the copyright holder.
To view a copy of this license, visit https://creativecommons.org/licenses/by/4.0/

We would like to thank all reviewers for their time, comments, and suggestions.

The changes in the main text and Supporting Information have been highlighted in yellow in the respective files. In addition, we have included in this document the new figures as a response to each comment.

Reviewer #1 (Remarks to the Author):

In this study, Samokhvalova and coworkers studied a unique system of coacervate, formed by the oxidation of a cysteine-containing polycationic peptide and a polyanion. By establishing the electrochemical oxidation of this cysteine-containing peptide, the authors are able to form coacervates by applying an electric field, rather than the conventional method of adding oxidizing agents. The authors then studied the motion of these droplets due to the electric field. The authors then went ahead to study the dissolution of these droplets by reduction of the cysteine-containing polycationic peptide, mediated by a reducing agent that is processed electrocatalytically, and studied the cyclic evolution of this process at steady state.

We thank the reviewer for their comments.

This steady state evolution of destruction and regeneration of droplets is not clear to me. While it is clearly visible that, in samples with MV present, there is a reduction in the size of the droplets near the cathode (Fig. 4c), the subsequent regeneration of droplets near the anode is not at all clear. Furthermore, the control videos are trimmed off at 6 or 8 hours, so one is unable to see if there are/aren't droplets at later times.
a. It would be useful to have the four videos, anode and cathode, with and without MV, displayed together in a single panel with synchronized time for the reader to appreciate the contrast between the two conditions.

We have recorded two new videos for the control samples (with and without MV), lasting up to 16 h. A synchronized comparison between these two videos and their equivalents with MV has been now included in Figure S16, as well as in a new Supplementary Video (Supplementary Video 3). We hope that this serves as a complement to the data shown in Figure 5.

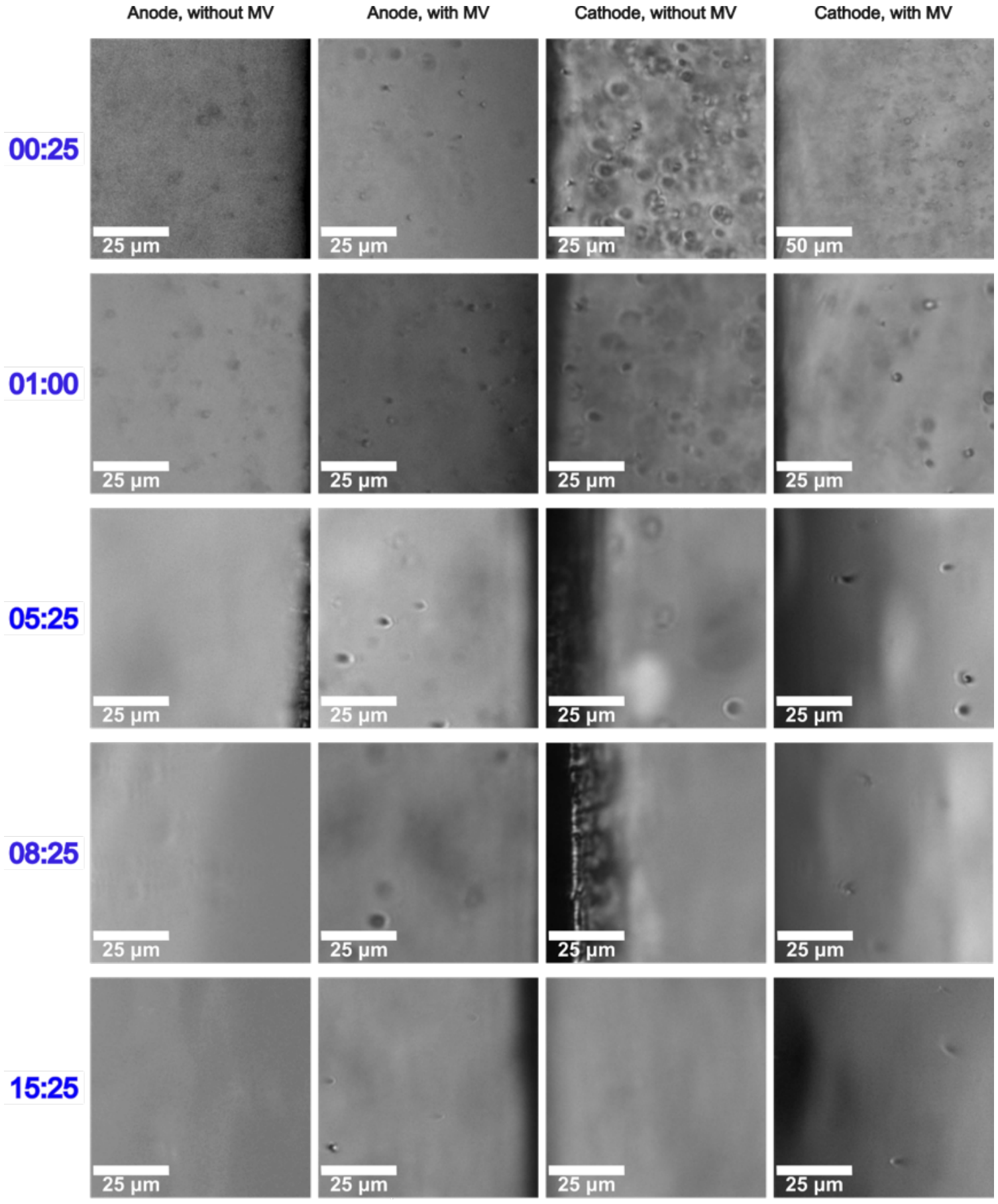

Figure S16. Micrographs of $(KKKC)_2$/pAA coacervates (2.5:1) next to the anode and cathode in presence and in absence of MV, at different times during the application of an electric potential (0.7 V). [KKKC] = 3.34 mM, [pAA] = 4 mM, [MV] = 6.68 mM. The size of the scale bars is indicated in the images. Times are reported as hh:mm.

b. A simple yet direct proof of regeneration would be to take the control system (without MV), allow it to achieve a steady state so that no droplets are present at the anode, and then add MV to show regeneration of droplets. A control study, where water without MV is added, must supplement this proof experiment to account for any mixing/convection flow of existing droplets.

We thank the reviewer for this suggestion, as this is indeed an excellent proof of the effect of MV. We have performed the proposed experiment in the following way. First, we have prepared two identical samples in absence of MV, and repeated the electrochemical cycle described in Figure 5, observing the formation of droplets for a few hours, followed by a drop in their number once that KKKC was depleted from the surface of the electrode. Once that practically no droplets were left on the proximity of the anode (< 5 droplets / 0.01 mm^2), we added either 16.7 μL of a stock solution of MV, or 16.7 μL of water, and recorded a timelapse in the same conditions for both samples. In the sample to which MV was added, we observed an increase in the number of droplets and a sustained steady state, while the control (with only water added) did not show a significant formation of new droplets

These results can be found in Figure S18 and Supplementary Video 4, and the corresponding discussion has been added to page 8 of the main text.

As a note, we followed the suggestion of another reviewer for this experiment, and prepared these samples in presence of a dye molecules that we used as a model cargo for the coacervates (Brooker's merocyanine), in order to show that the compartmentalization of guest molecules does not affect the formation and destruction of droplets. Both the MV sample and the water control contained this dye.

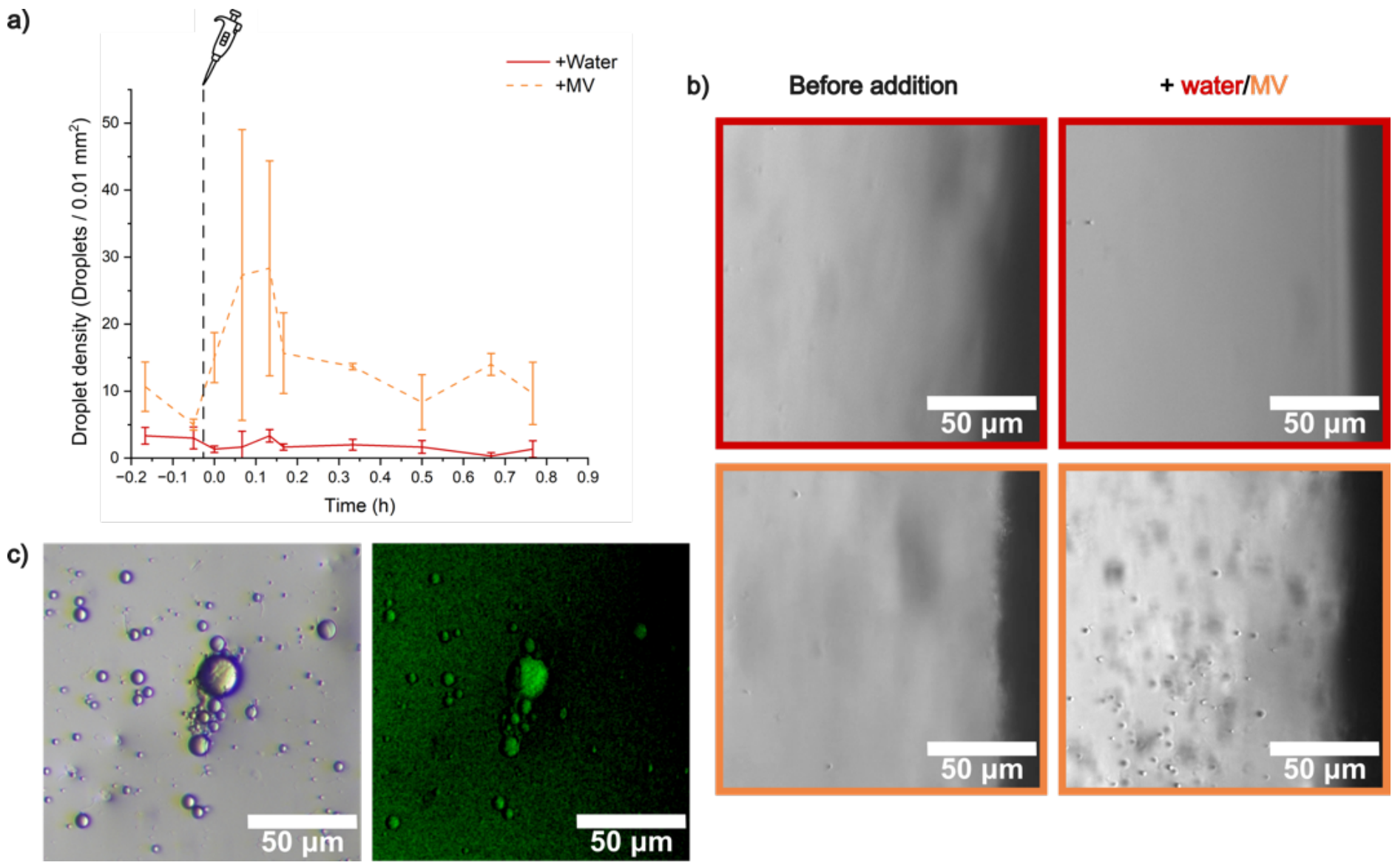

Figure S18. Regeneration of coacervates by addition of MV, after KKKC had been depleted from the anode. a) Average droplet density in the proximity of the anode before and after the addition of MV (orange dashed line). An identical sample, to which water was added instead of MV, is represented in red. The reported numbers correspond to the average of three 100 x 100 µm areas next to the surface of the electrode, and the error bars to their standard deviation. b) Micrographs of the area next to the anode in both samples, before and after addition of MV or water. c) Brightfield and fluorescence micrographs of coacervates in the bottom of the cell, showing the encapsulation of Brooker's merocyanine. [KKKC] = 3.34 mM, [pAA] = 4 mM, [MV] = 33.34 mM, [Brooker's merocyanine] = 0.95 µM. The scale bars are 50 µm.

While the concept and results are unique, the study is quite incomplete, and the manuscript does not contribute significantly to the understanding of these new systems.

Some other comments

1. In the caption of Figure 1, MV is not defined. After going through the manuscript, it became clear that it refers to Methyl Viologen. Please define it at the first occurrence for clarity.

We have added the complete name of MV to the caption of Figure 1, as well as its structure.

2. On page 3, the authors note that the KKKC/ATP system forms droplets under an electric field, while (KKKC)2/ATP did not form droplets in the absence of an electric field. Could this be because of the pKa shift of ATP in the presence of an applied electric field? Many studies, including this recent one

(https://pubs.acs.org/doi/10.1021/jacs.3c13633), have shown that applied potential tunes protonation state at fixed bulk pH, implying significant shifts in pKa driven by local interfacial electric fields. Thus, it is possible that ATP3- is converting to ATP4- under an electric field, allowing complexation with KKKC2. Could the authors comment on this possibility? Perhaps some measurements can be done to know if this pKa/pH change is indeed leading to coacervation in this system by 1) checking if ATP4- (at pH > 8) can complex with KKKC2 in the absence of an e-field, and 2) adding borate salts to the droplets formed under an e-field to check their salt resistance.

By the suggestion of the reviewer, we have screened the complexation between ATP and $KKKC_2$ at basic pHs, to ensure the complete deprotonation of ATP (Figure S7). No coacervates were observed in these conditions, which refutes the hypothesis about pKa modulation by the electric field.

However, when we performed the second proposed experiment (addition of borate salts to the $KKKC_2$/ATP coacervates formed by electrochemistry), we were surprised to discover that their dissolution only took place at very large salt concentrations (> 10 equivalents), much larger than what could be generated as a side-product of oxidation.

This led us to examine in more detail the phase diagram of the $KKKC_2$/ATP system (Figure S8). Remarkably, our experiments indicate that the reason why $KKKC_2$/ATP coacervates form in the electrochemical setup but not by chemical oxidation is the presence of Ag^+ ions, which can form by degradation of the electrode. As seen in Figure S8, Ag^+ ions increase the salt resistance of complex coacervates of both $KKKC_2$/ATP and $KKKC_2$/pAA (even though for the latter, oxidation leads to the formation of coacervates with or without silver). We hypothesize that the presence of Ag^+ ions leads to the formation of peptide-silver complexes with increased multivalency, which are more prone to coacervation. However, this is a completely unprecedented result, and determining the exact effect of silver requires further investigation beyond the scope of this publication.

In addition to Figures S7 and S8, we have removed the parts of the text where we referred to the salt by-products of $NaBO_3$ as the main difference between the chemical and electrochemical systems, and replaced them with a discussion of these new results (changes in page 3 and in the caption of Figure 2, as well as addition of reference [53]). We believe that these changes do not affect the main conclusions of the manuscript: oxidation is still the main driving force for coacervate formation, and most of the experiments of the manuscript were done on the $KKKC_2$/pAA system, for which Ag^+ does not have such a drastic effect.

We want to thank the reviewer for their suggestion, which has led to a better understanding of the $KKKC_2$/ATP system, as well as uncovered an interesting synergy between metal ions and coacervation which we will continue investigating in the future.

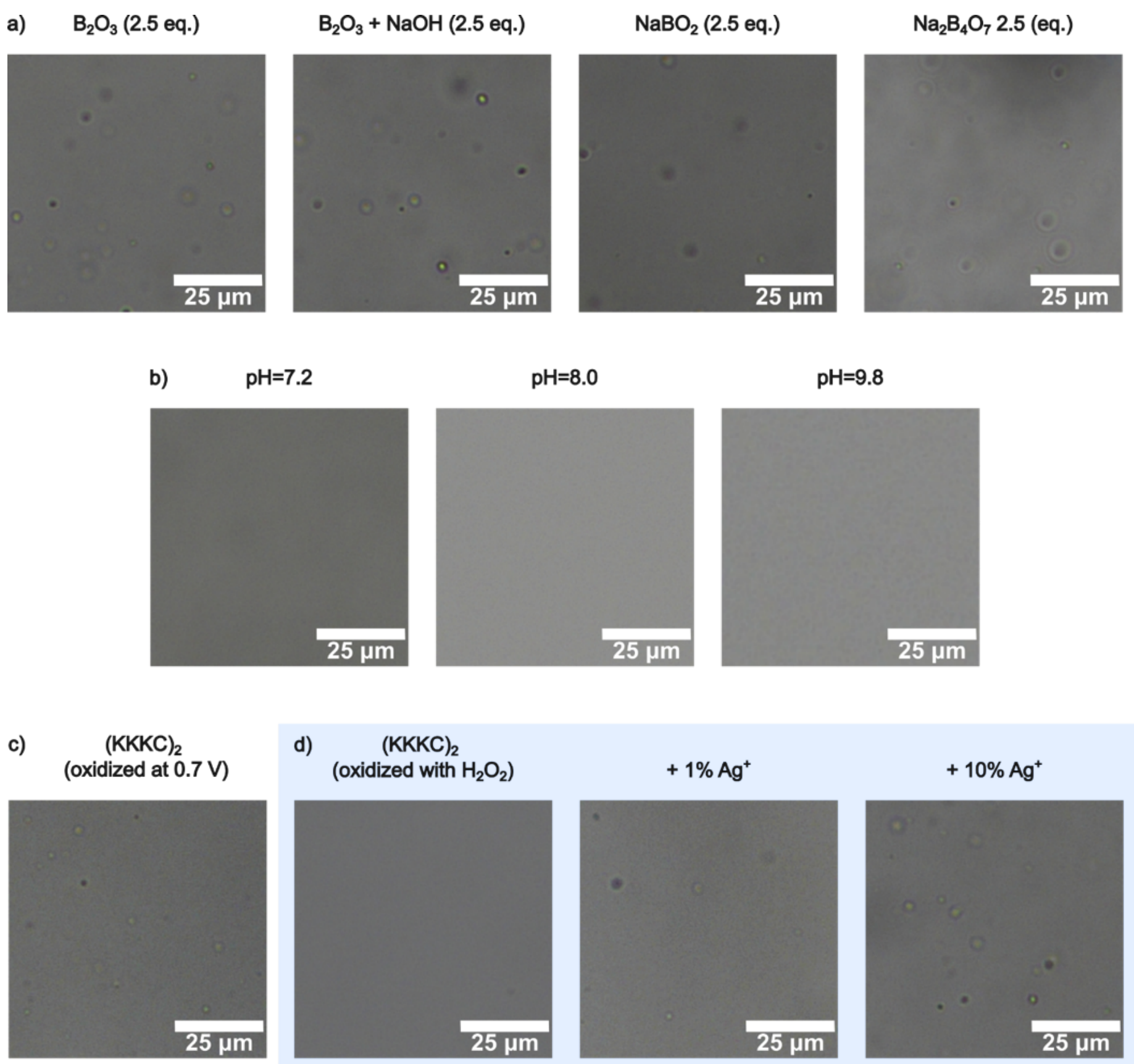

Figure S7. Micrographs of 1:1 KKKC:ATP mixtures in various conditions. a) $(KKKC)_2$:ATP coacervates prepared via electrochemical oxidation (0.7 V, 1 hour), followed by the addition of boric anhydride (B_2O_3), a mixture of B_2O_3 and NaOH, sodium metaborate ($NaBO_2$), or sodium tetraborate ($Na_2B_4O_7$) (3.35 mM of each salt). b) KKKC:ATP mixtures prepared at different pH values and incubated with sodium perborate (3.35 mM, 2.5 eq.) for 2 hours. c) $(KKKC)_2$:ATP coacervates prepared by mixing electrochemically-prepared $(KKKC)_2$ (0.7 V, 40 min) and ATP. d) $(KKKC)_2$:ATP mixtures prepared by chemical oxidation of KKKC with H_2O_2, in presence of different concentrations of Ag^+. Silver ions were added from a 5 mM stock solution of $AgNO_3$ (1 or 10 molar % of Ag^+ with respect to KKKC). [KKKC] = 1.34 mM, [ATP] = 1.34 mM (in monomer units). The scale bars are 25 µm.

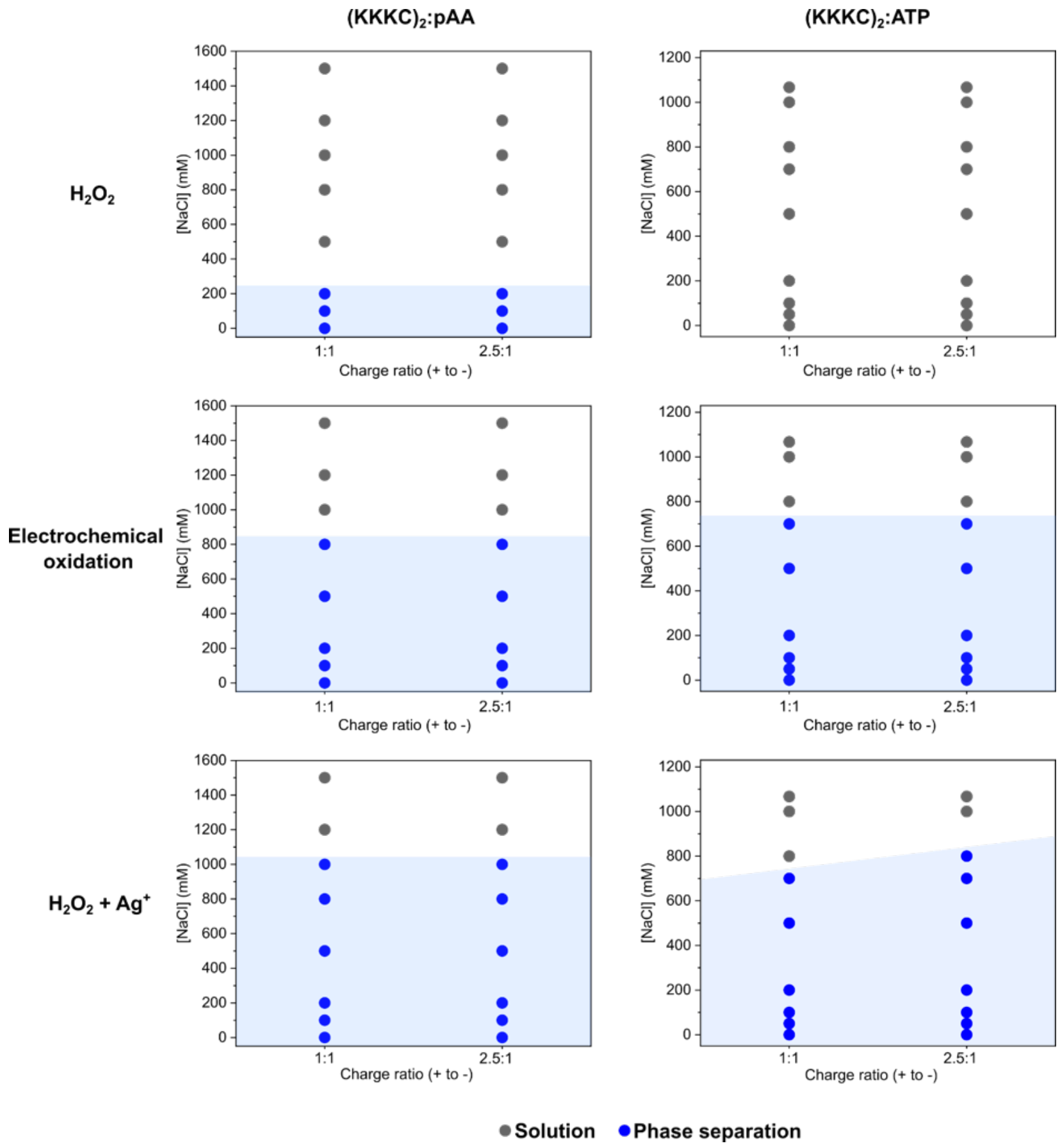

Figure S8. Phase diagrams of (KKKC)$_2$/pAA and (KKKC)$_2$/ATP coacervates prepared in three different conditions: 1) Chemical oxidation of KKKC with H_2O_2 (22 eq.) followed by mixing with ATP or pAA. 2) Electrochemical oxidation of KKKC followed by mixing with ATP or pAA. 3) Chemical oxidation of KKKC followed by addition of silver nitrate (10 molar % of Ag^+ with respect to KKKC). [KKKC] = 1.34 mM or 3.34 mM (for the 1:1 and 2.5:1 mixtures, respectively), [pAA] = 4 mM, [ATP] = 1.34 mM (in monomer units). The same titrations were repeated in reducing conditions (without H_2O_2 and with no electrochemical oxidation, in presence of 3.35 mM of TCEP), and no phase separation could be detected at any salt concentration or mixing ratio.

3. To prevent the wetting of the glass surface by coacervate droplets, surface coatings such as BSA, PEG, silane, or PVP can be employed. An earlier paper studying coacervate droplets under an electric field did such coating to prevent droplet wetting (https://doi.org/10.1073/pnas.2203483119). This may allow observation and circulation of droplets for an extended period of time.

The cited publication uses a microwell plate with a glass bottom, which indeed requires surface modification to avoid the adherence of the coacervates to it. In the new confocal experiments that we have included in the current manuscript (Figure S24), we have also used glass slides, and we have coated them with PVA. However, for most of the experiments in this publication, we have used a well plate with a polymeric surface (Ibidi 96-square well plate) to which the coacervates do not adhere very strongly even without surface modifications.

Nonetheless, we agree that for further experiments (especially, those done in smaller volumes) we will need to explore different surface functionalizations to minimize droplet wetting.

Reviewer #2 (Remarks to the Author):

I co-reviewed this manuscript with one of the reviewers who provided the listed reports. This is part of the Nature Communications initiative to facilitate training in peer review and to provide appropriate recognition for Early Career Researchers who co-review manuscripts.

We thank the reviewer for their contribution.

Reviewer #3 (Remarks to the Author):

Designing active coacervate droplets has garnered significant interest for mimicking biological cellular compartments, particularly focusing on the regular creation of coacervates out of equilibrium. In this work, the authors propose an electricity-driven system for the transient formation, directional motion, and dissolution of complex coacervates. The design involves coupling a cysteine-containing peptide through electrochemical oxidation, with the double multivalency triggering ionic complexation with a polyanion, leading to coacervate formation at the anode. The resulting droplets migrate electrophoretically over a relatively long distance toward the cathode, where the electrocatalytic cycle dissolves the coacervates through reduction of the peptide. The free peptide and polyanion are then recovered, diffuse back to the anode, and react again, establishing a steady state in which droplets continuously form, move, and disappear in different regions of the cell. This steady cycling arises from the prevention of side product generation during the electrocatalytic process. The authors also demonstrate that the electrochemically formed droplets can spontaneously sequester solutes from the surrounding solution, enabling potential applications in electrically driven cargo transport and gradient formation. While the work generally validates the transient formation and movement of coacervate droplets powered by electricity, several important concerns remain regarding a deeper analysis of the coacervation process and further clarification of the related statements. These issues, outlined below, need to be thoroughly addressed before the study can be considered for publication in Nature Communications.

We thank the reviewer for their comments and for the suggested experiments.

1.In the title, the authors refer to the system as a “Waste-Less System,” which does not seem to align well with the statement in the abstract that claims “no side-product.” Does the cycle completely eliminate side products? What potential by-products could arise during the electrocatalytic process? Further clarification on this point is recommended, including additional analysis of the system before and after recycling. For instance, an analysis of the pH and ionic strength of the solution, as well as qualitative and quantitative analysis using 1H NMR spectra to identify chemicals and their concentrations, would help to better understand any possible side products.

We refer to this system as “Waste-less” as a comparison to other chemically fueled reaction cycles, maintaining the terminology from that field. In these reaction cycles, an exergonic chemical reaction is used to populate a thermodynamically unstable state, driving the system out-of-equilibrium. The most commonly used terminology for these systems uses “fuel” to describe the reagents of this exergonic reaction, and “waste” to describe its products. Waste accumulation refers to a common problem in these systems, in which the products of the exergonic reaction accumulate in the reaction medium, which interferes with the reaction cycle over time. Examples of waste accumulation can be found in Donau et al. *Nat. Commun.* **2020** (Ref. [22] in the text, Figure S5 of their publication), or Nakashima et al. *Soft Matter* **2018** (Ref. [27], Figure 4c of their publication). Chen et al, *Chem. Sci.* **2023** (Ref. [34]) describes some recent strategies to address this problem by tuning the reaction conditions.

Electrically-fueled systems, in principle, avoid waste accumulation, as the driving force keeping the system out-of-equilibrium is not the conversion between high-energy fuel molecules and low-energy waste molecules, but instead an electric potential that is introduced externally. In other words, only electrons enter or leave the system, and all the individual reactions happening in the cell cancel each other. Electrically-fueled systems have been described as waste-free before (Barpuzary et al. *J. Am. Chem. Soc.* **2023**, ref. [37]), a terminology that we follow here.

So, in that sense, our system is waste-less by design: no side-products/waste should form in the chemical reaction cycle itself. However, we agree with the reviewer that, in practice, the composition of the medium can change over the long experimental times: due to electrode degradation, water oxidization / reduction on the electrode surface, or water evaporation. In order to characterize these changes in detail, we have measured the conductivity and pH of the system over the course of a 21-hour electrochemical experiment (Figure S19). Our results show a negligible change in conductivity during this time, indicating that practically no ions are being accumulated in the medium. The pH does increase during these 21 h, going from 7 to 8, possibly due to the reduction of dissolved O_2 at the beginning of the reaction (see note [45] on the main text). However, this change in pH does not significantly affect coacervate formation in our systems. (Figure S20).

In addition to the pH and conductivity experiments, we have analyzed the reaction mixture by ^{1}H-NMR before and after 21 h of continuous electrochemical cycling (Figure S21). Doing quantitative analysis on these spectra is difficult, as the system is a mixture of four different compounds (KKKC, pAA, MV and buffer), out of which two undergo phase separation and coalesce (so their relative signals change over time). Nonetheless, we could not detect the presence of any significant impurities in the 21 h spectrum, and we could still observe the signals corresponding to pAA, oxidized KKKC and MV (with a slight shift attributed to the change in pH).

Besides the new Supplementary Figures, we have included a discussion on this characterization and the waste-less nature of our system in Page 8.

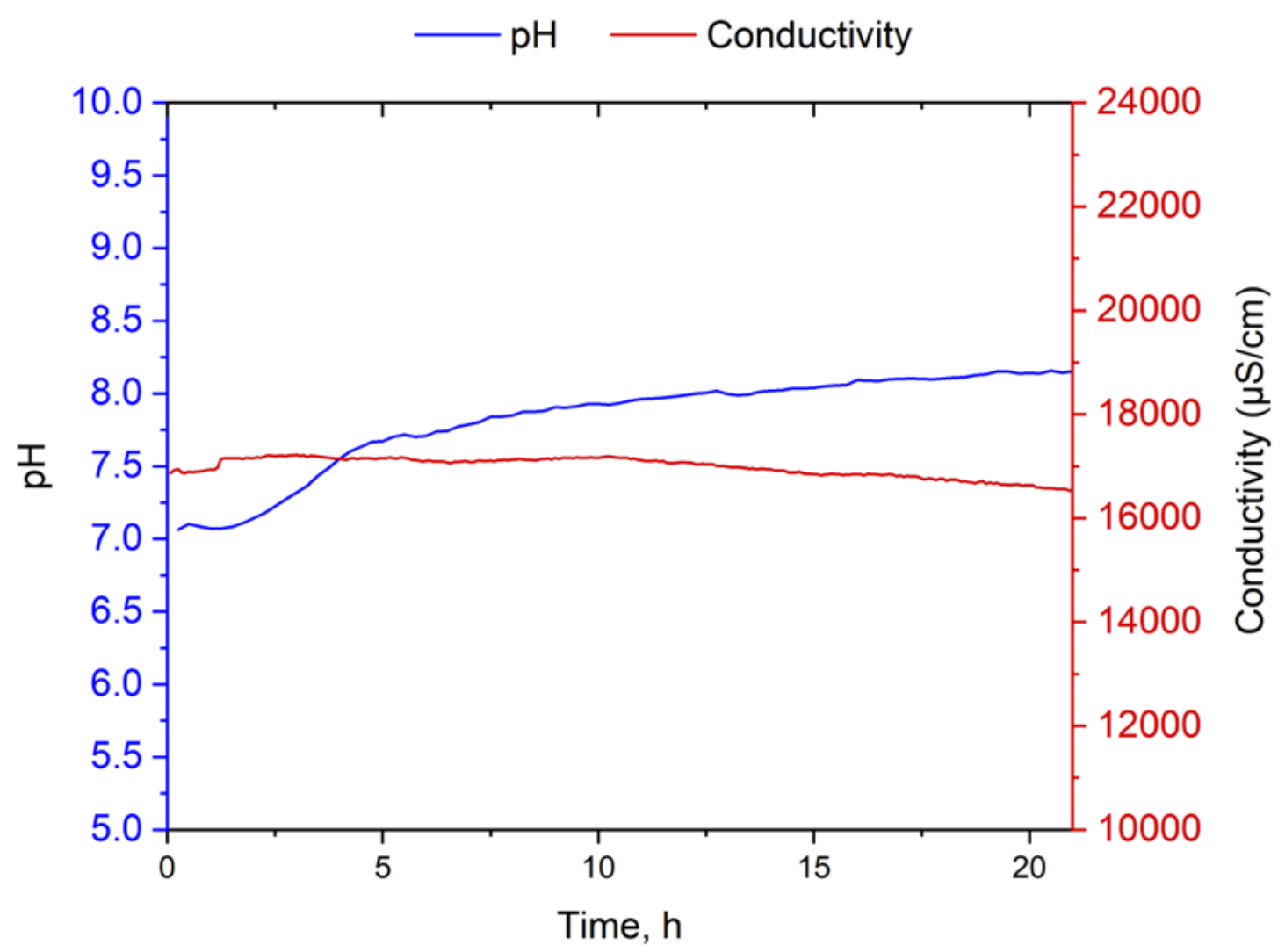

Figure S19. pH and conductivity monitored over a 21-hour electrochemical experiment (0.7 V) in a 2.5:1 KKKC/pAA system in presence of MV. [KKKC] = 3.34 mM, [pAA] = 4 mM, [MV] = 6.68 mM.

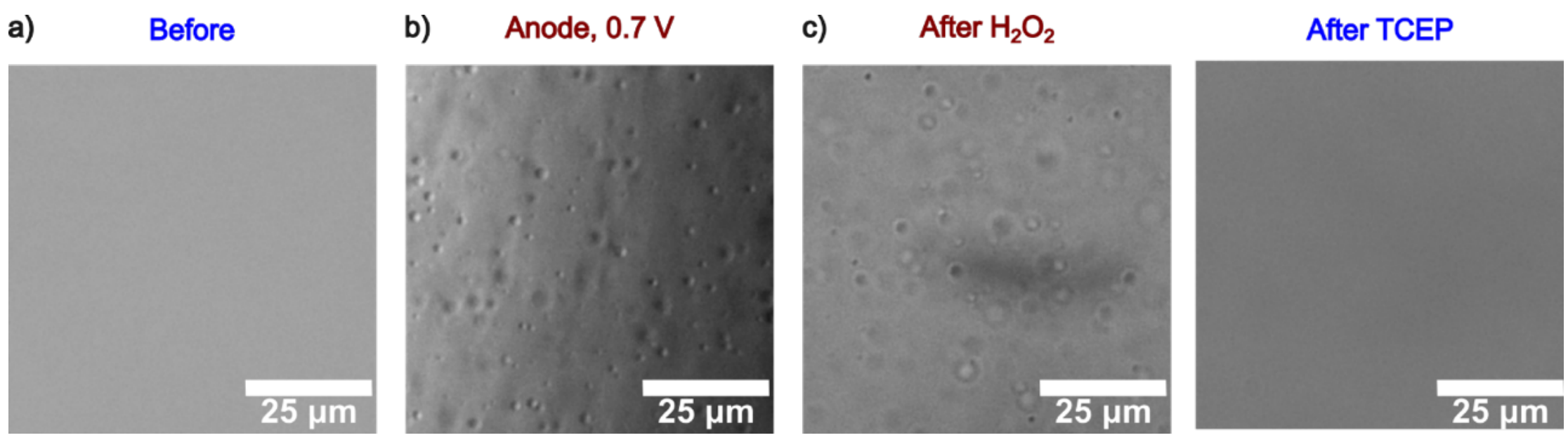

Figure S20. Electrochemical oxidation and reduction of KKKC-based coacervates prepared with pH = 8.1. Micrographs of a 2.5:1 KKKC:pAA sample with pH = 8.1 a) immediately after preparation ; b) in the proximity of the surface of the anode, 2 minutes after the application of a 0.7 V potential; c) 2 minutes after the addition of H_2O_2 (29.4 mM, 8.8 eq.) to a freshly prepared sample, and d) immediately after the addition of TCEP (32.1 mM, 9.6 eq.). [KKKC] = 3.34 mM, [pAA] = 4 mM (in monomer units). All samples contain Tris buffer (pH = 8.1, 50 mM) and NaCl (100 mM). All scale bars are 25 µm.

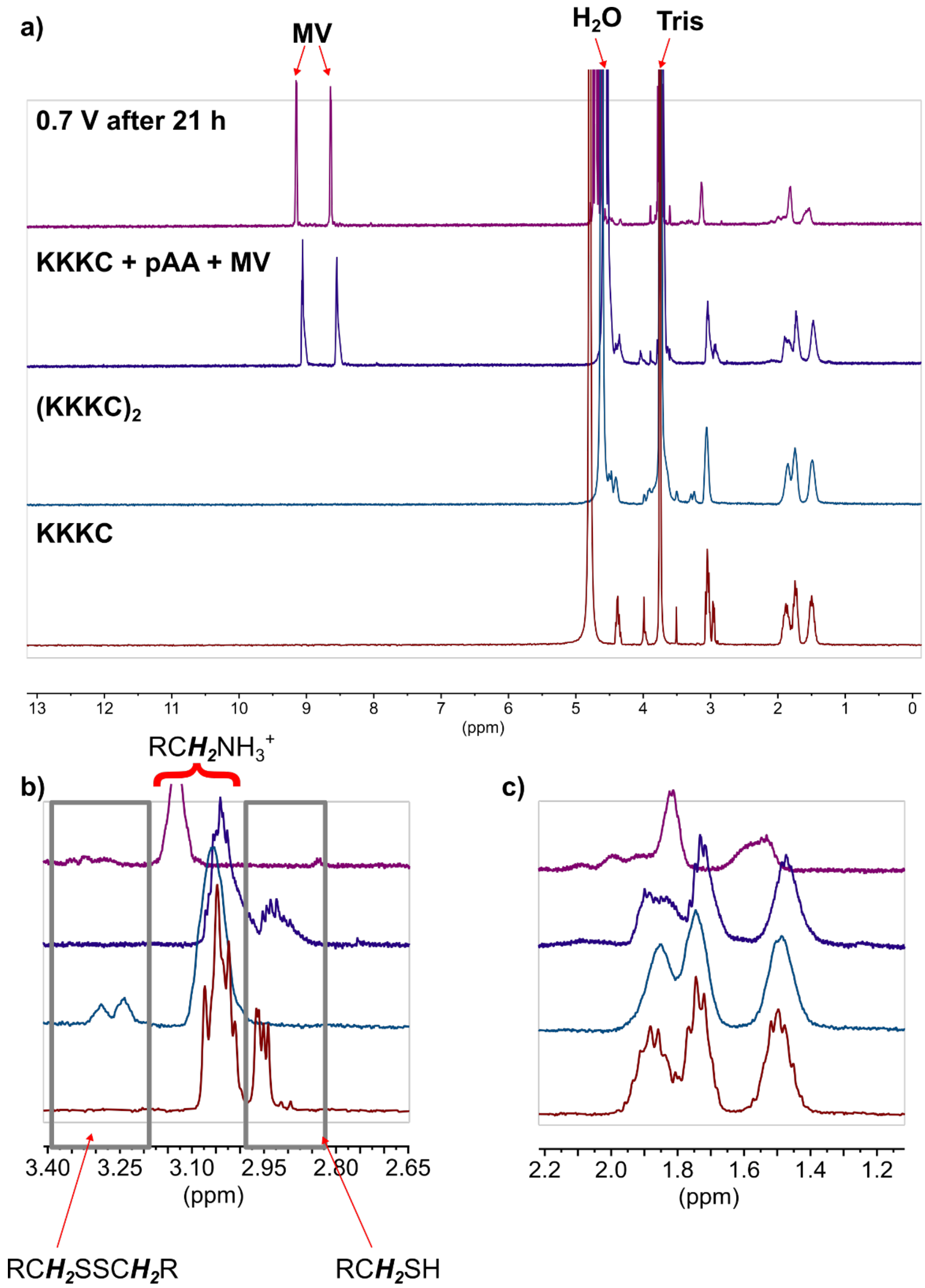

Figure S21. ^{1}H-NMR spectra (300 MHz, D_2O) corresponding to a 2.5:1 KKKC/pAA mixture after continuous electrochemical cycling (0.7 V) for 21 hours in presence of MV; a freshly-prepared mixture before application of the potential, $(KKKC)_2$ obtained by electrochemical oxidation, and fresh KKKC solution. The full spectra are reported (a), as well as insets showing b) the signals corresponding to the CH_2 protons next to the thiol/disulfide groups and amino groups of the side chains of KKKC and c) the aliphatic region, where the rest of the side chain signals overlap with those coming from the backbone of pAA.

2.The control factors for phase separation are not sufficiently investigated in the first part of the study. The authors primarily rely on qualitative optical microscopy to illustrate coacervate formation. A more comprehensive investigation of the control factors (such as mixing ratio and ionic strength) using quantitative analysis, such as turbidity measurements and fluorescence imaging of the droplets, is recommended to fully define the phase separation in the system. Similarly, the dissociation of the coacervates could also be further clarified through quantitative analysis.

We have now used turbidity measurements to characterize the phase separation of the $KKKC_2$/pAA and $KKKC_2$/ATP systems at different mixing ratios. The phase diagrams resulting from these studied can be found in Figure S8. We have also performed the same titrations in completely reduced systems (KKKC/pAA and KKKC/ATP), not observing coacervate formation at any salt concentration.

Over the course of the revisions, we have discovered that phase separation in these systems is not only influenced by ionic strength (as it is the case for most coacervates), but also by the presence of silver ions in the solution. To the best of our knowledge, this is a unique feature of this system, which we attribute to the formation of peptide-silver complexes with increased multivalency. To characterize that effect, we have also repeated the turbidimetric titrations in presence of $AgNO_3$ (Figure S8, panels 5 and 6).

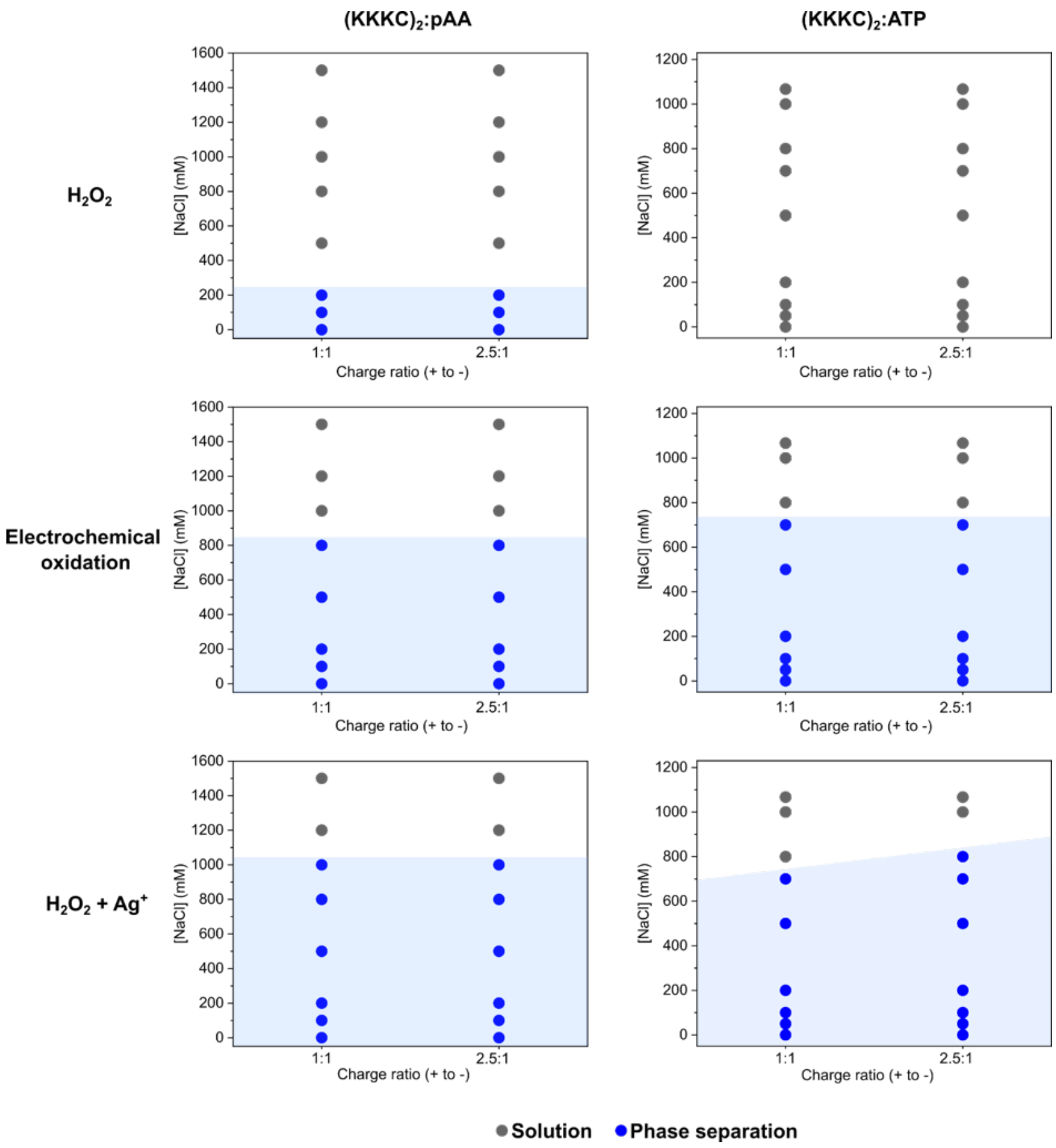

Figure S8. Phase diagrams of $(KKKC)_2$/pAA and $(KKKC)_2$/ATP coacervates prepared in three different conditions: 1) Chemical oxidation of KKKC with H_2O_2 (22 eq.) followed by mixing with ATP or pAA. 2) Electrochemical oxidation of KKKC followed by mixing with ATP or pAA. 3) Chemical oxidation of KKKC followed by addition of silver nitrate (10 molar % of Ag^+ with respect to KKKC). [KKKC] = 1.34 mM or 3.34 mM (for the 1:1 and 2.5:1 mixtures, respectively), [pAA] = 4 mM, [ATP] = 1.34 mM (in monomer units). The same titrations were repeated in reducing conditions (without H_2O_2 and with no electrochemical oxidation, in presence of 3.35 mM of TCEP), and no phase separation could be detected at any salt concentration or mixing ratio.

3.General capability of the proposed strategy: According to Figure 1, the key feature of the peptide design is the terminal thiol group of KKKC, which can be oxidized to double the charge and trigger phase separation. Do the other sequences in the peptide also play a role in regulating phase separation? What would happen if the terminal thiol group is retained but the peptide sequence is varied?

We thank the reviewer for this question: it is indeed in our plans to study different peptide sequences in this process. The effect of the peptide sequence in the phase-separation of peptide polyelectrolytes has been previously studied for different systems, notably by the group of Sarah Perry (See Chang et al. *Nat. Commun.* **2017** https://doi.org/10.1038/s41467-017-01249-1, or Sathyavageeswaran et al. *Biomacromolecules* **2025** https://doi.org/10.1021/acs.biomac.5c00773). We expect similar trends in our redox-sensitive systems: increasing the length of the peptide chain or including hydrophobic amino acids should increase the salt resistance of the coacervates, for example. In addition to this, increasing the charge of the peptides should also influence the speed at which the coacervates move from one electrode to another, by changing their surface charge (See van Haren et al. *Nat. Commun.* **2024**, ref [42]). Finally, for further studies involving the compartmentalization of cargo molecules and their active transport, the peptide sequence could also be changed to tune the partition constant (e.g. by including aromatic amino-acids to facilitate the partition of aromatic molecules).

We are currently studying different peptide sequences in this context, and our preliminary studies seem to confirm these hypotheses. However, a thorough characterization is outside of the scope of this proof-of-concept study, and will be done in a separate publication.

4.The details regarding the measurements of the coacervate droplets (such as Zeta potentials, size, morphology, etc.) in this dynamic and flowing system need to be supplemented.

We have characterized the size and morphology of the different coacervate droplets produced in this study, and added this information to the Supporting information (Figure S10, discussion in page 4). In summary, all coacervates have a similar size (4-8 μm in diameter) except for the ones formed from $KKKC_2$ and ATP in a 1:1 ratio, which are significantly smaller. All droplets are spherical when they are formed and when they are travelling through the solution (their circularities are never significantly different than 1), and they spread and coalesce with each other when they accumulate on the bottom of the plate (Figure S11). Shortly, they behave in the way expected for an emulsion formed by LLPS.

We have also characterized the size and circularity of the coacervates during the 21-h electrochemical experiments in presence of MV. While they all remained as spherical droplets, we observed a slight increase in their size over time (from ~5 to ~10 μm) (Figure S22). The reason behind this change could be related to a change in the composition of the solution in the proximity of the anode (as KKKC and pAA might not diffuse back from the cathode at the same speed), or to a selection based on the speed of the droplets (small droplets are known to move more slowly than large ones in an electric field, so small droplets could remain in the close proximity of the anode until they are large enough to move to the other electrode). We believe that characterizing this phenomenon in detail merits another study, however, we have included our experiments in the Supporting Information, and discussed our findings in the main text. (Page 8)

In terms of the Z-potential – recent work from Spruijt (van Haren et al. *Nat. Commun.* **2024**, ref [42]) has shown that it cannot be calculated for coacervates using a standard setup: the Smoluchowski equation that is used in standard Z-sizers assumes that the moving particles are rigid, which is not the case for coacervates. Determining the exact Z-potential using a more appropriate method would require an extensive physicochemical characterization of the coacervates (in particular, their viscosity would need to be measured), which is difficult for coacervates due to their small volume.

For this reason, we prefer to not give incorrect or approximated Z-values for our systems, and we report instead their relative movement speed in an electric field (Figure 2), which gives a direct comparison between their charges. We have included this discussion to the text (Page 5).

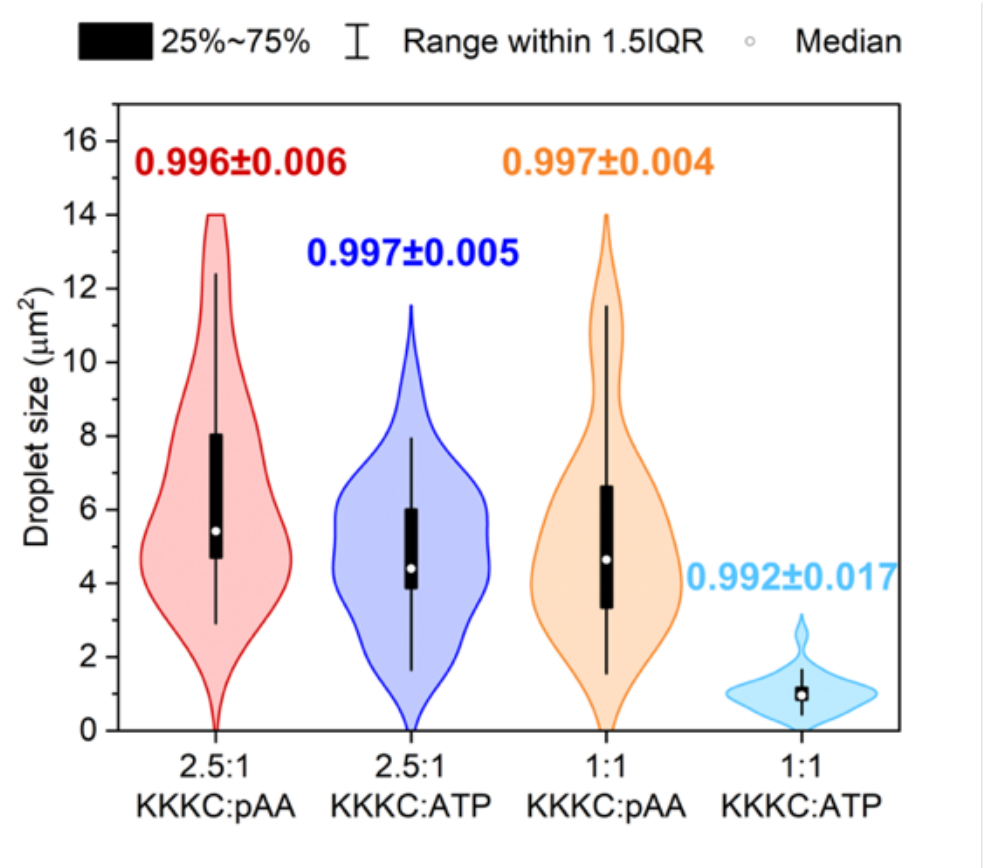

Figure S10. Size distributions of coacervates prepared by the electrochemical oxidation (0.7 V) of KKKC in presence of either pAA or ATP in different mixing ratios. 25 droplets were analyzed for each sample. The circularity of the droplets is reported above the corresponding violin plots (as average ± standard deviation). [KKKC] = 1.34 mM or 3.34 mM (for 1:1 and 2.5:1 mixtures respectively), [pAA] = 4 mM, [ATP] = 1.34 mM (in monomer units). The samples contained Tris buffer (50 mM, pH = 7.4) and NaCl (10 mM for ATP samples, 100 mM for pAA samples).

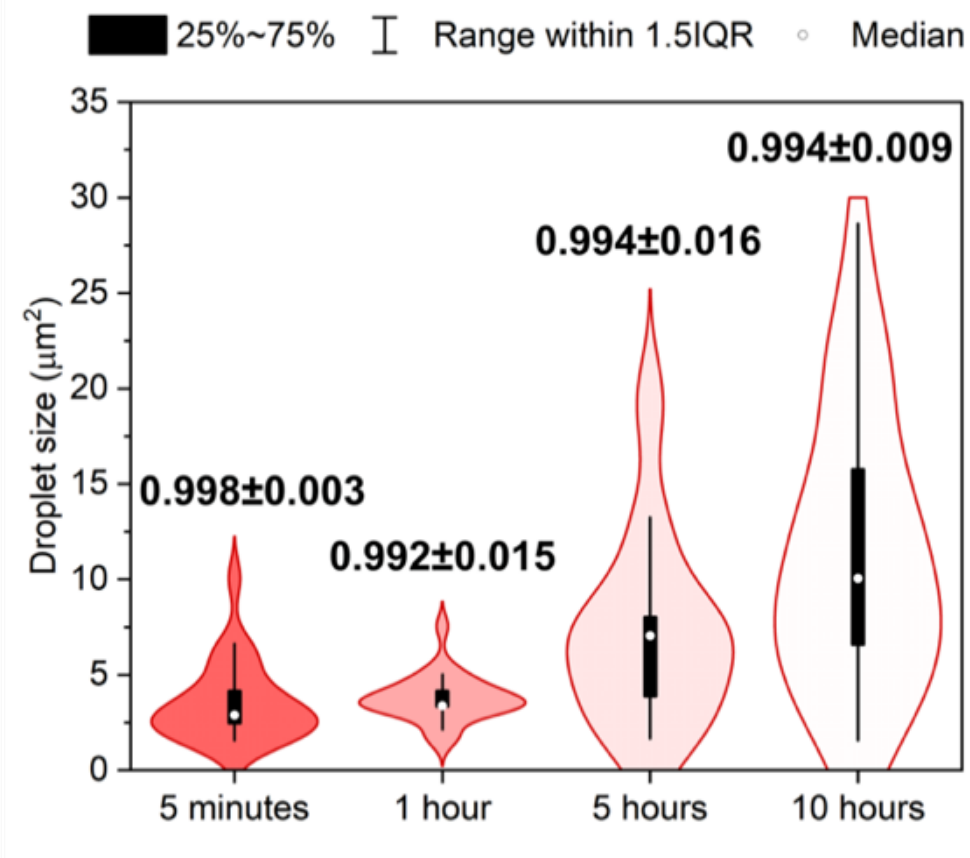

Figure S22. Size distributions of the coacervates obtained at different times during the electrochemical cycle (0.7V) in presence of MV. 25 droplets were analyzed at each time. The circularity of the droplets is reported above the corresponding violin plot (as average ± standard deviation). [KKKC] = 3.34 mM, [pAA] = 4 mM in monomer units, [MV] = 6.68 mM. The samples contained Tris buffer (50 mM, pH = 7.4) and NaCl (10 mM for ATP samples, 100 mM for pAA samples).

5.Page 6, "The intensity of the color was significantly stronger within the coacervates than in the surrounding solution, indicating that the diffusion of MV·+ into the coacervate phase is a favorable process." The authors are suggested to provide more quantitative analysis, for example, the partition coefficient of MV·+ to support this claim. Additionally, does the flow affect the partition coefficient compared to conventional coacervation?

We cannot quantify the partition of $MV^{\cdot+}$ into our coacervates – as $MV^{\cdot+}$ quickly reacts with the disulfide groups and dissolves the coacervate phase (Figure 4), so an equilibrium in concentration between the two phases cannot be reached. However, we believe that the blue color observed inside of the coacervate phase in Figure 4c is enough proof to (qualitatively) say that this partition is favorable. In addition, our proposed mechanism (reduction of MV to $MV^{\cdot+}$, and subsequent reaction of the radical cation with the disulfides in the coacervate) is also supported by our CV experiments and the experiments in Figure 5 – a steady state is only reached in presence of MV.

About the second part of the question, regarding whether the movement of coacervates affects the partition. This is a very interesting point. To the best of our knowledge, the composition of the coacervate phase does not change when the electric field is applied, so the partition coefficient of MV between the two phases should remain the same. However, the partition coefficient is a thermodynamic quantity that assumes that the concentration of solute is in equilibrium between the two phases. When the electric potential is applied, several concentration gradients appear: the concentration of MV decreases in the proximity of the cathode, KKKC and pAA are depleted from the area next to the anode (as the coacervates are being formed and moving away by electrophoresis), and KKKC and pAA accumulate on the cathode surface as well. The combination of these different concentration gradients is likely to change the partition of different molecules, not because of a change in Kp but because the system is out-of-equilibrium. This discussion has been added to the main text in the context of the compartmentalization and transport of dyes. (Page 8)

6.The final part regarding the incubation of different fluorescent dyes by coacervates requires further study, as it serves as a key proof supporting the potential application of the proposed coacervate regulation. Ideally, the dye molecules should be classified into different categories, and the partitioning should be analyzed using both qualitative and quantitative methods to explore the principles behind selective encapsulation.

We have studied the partition of the different dyes in all the coacervates described in this study, using confocal microscopy. These results are now shown in Figure S24 and Figure S25, where we have organized the partition coefficients by dye and droplet charge. There does not seem to be a clear correlation between the charge of the dyes, the charge of the coacervates, and extent of compartmentalization. However, we are continuing to work in this direction for our future studies, together with a systematic screening of peptide sequences as mentioned above.

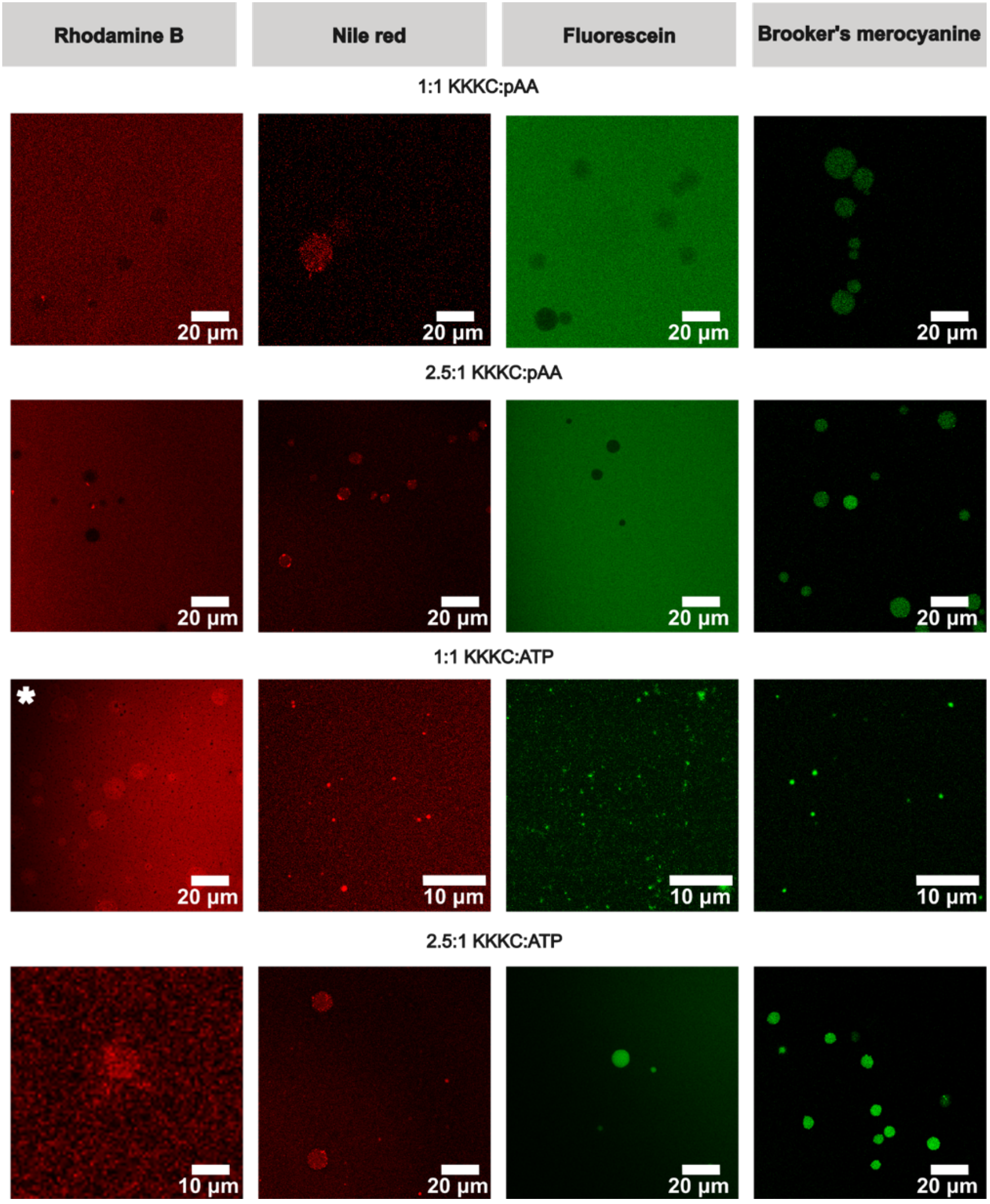

Figure S24. Confocal microscopy images for KKKC/pAA and KKKC/ATP coacervates in presence of neutral (Nile Red, Brooker's merocyanine), and charged (fluorescein and Rhodamine B) dyes. All samples except for 1:1 KKKC:ATP with Rhodamine B were prepared in the same conditions and with the same concentrations as those in Figure S23, followed by deposition on a PVA-coated slide and incubation for one (for pAA coacervates) or two (for ATP coacervates) days before imaging. For the 1:1 KKKC:ATP sample containing Rhodamine B (marked with an asterisk), the concentrations of KKKC, ATP, and dye were doubled to facilitate imaging.

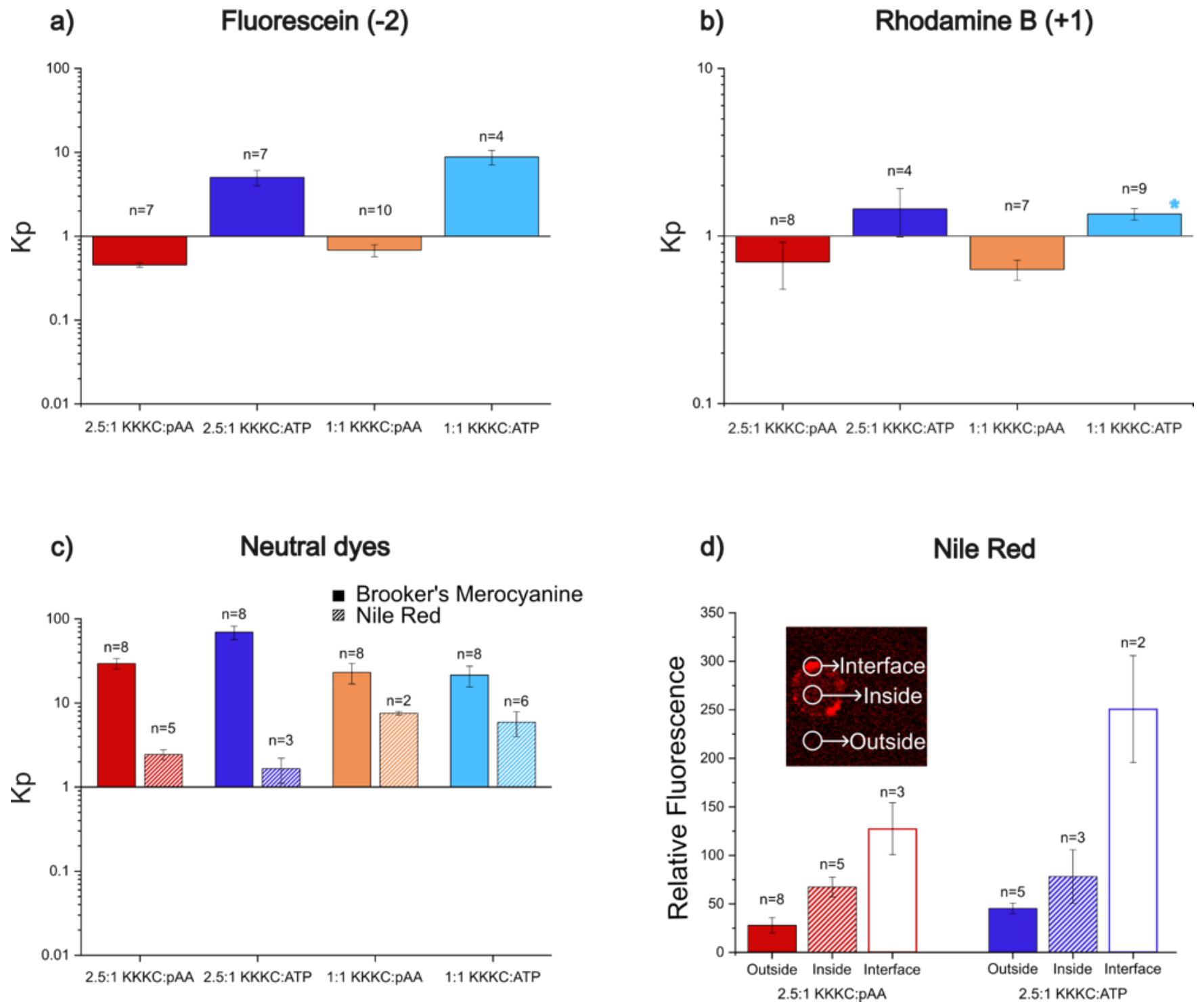

Figure S25. Partition coefficient (*Kp*) of a) a negatively-charged dye (Fluorescein), b) a positively charged dye (Rhodamine B) and c) two neutral dyes (Brooker's merocyanine and Nile Red), calculated from the confocal images on Figure S24. d) Relative fluorescence intensity of Nile Red outside, inside and on the border of the coacervate droplets, illustrating the higher accumulation of the dye molecules in the interface. The reported values correspond to the average of 2 to 9 samples (indicated over the bar) and the error bars represent the standard deviation. The asterisk in the 1:1 KKKC:ATP sample for Rhodamine B indicates that the concentration of polyelectrolytes and dye was doubled in this sample (see Figure S24)

7.The quality of the images and figures could be improved in terms of both aesthetics and clarity.

Reviewer #4 (Remarks to the Author):

In this manuscript, authors have presented the relatively new approach to make coacervates through oxidation of cysteine amino acids in the design of tetrapeptide. This approach brings the way forward to avoid the formation of side-products during the coacervation process of out-of-equilibrium droplets, using electrocatalytically mediated oxidation and reduction. Authors have claimed the movement of coacervates, self-destruction and reformation within the same system. Overall, it is interesting study, and I'd recommend authors to improve the manuscript with following comments.

We thank the reviewer for their comments and for the suggested experiments.

1. KKKC can have 3+ charges while pAA is a polymer and I'm wondering how the charges are balanced in final product (coacervates) and how exactly the deprotonation happens to bring the negative charges on pAA motif?

Typically, an imbalance in the length of the two components of a complex coacervate creates an excess of the charge of the longest component. This is due to the different dissociation entropies of a small molecule and a large one: larger ones are more difficult to dissociate due to cooperativity. (Majee et al. *Phys. Rev. Res.* **2024,** https://doi.org/10.1103/PhysRevResearch.6.033138) However, this effect is often compensated or overcome by other interactions, and multiple "length-imbalanced" coacervates have been described to be enriched on the smaller component (van Haren et al. *Nat. Commun.* **2024**, ref [42]).

In our case, mixing $KKKC_2$ and pAA in a 1:1 ratio in terms of charges results in coacervates that are very close to electric neutrality, as shown by Figure 3c. This indicates that either there is no imbalance in composition arising from the difference in lengths, or that this effect is compensated by another factor that favors the incorporation of $KKKC_2$ in the coacervate phase.

In terms of the protonation/deprotonation of the different components, the pH in all of the experiments remained between 7 and 8, while the pKas of the Lysine and acrylic acid groups are ~10 and ~6, respectively. Therefore, both of the partners should be completely charged in the range of pHs used.

2. To make this more versatile, other oxidizing agents such as TCEP, DTT should be tried as well.

We thank the reviewer for this suggestion. In addition to studying the oxidation of KKKC with $NaBO_3$ (which we already had in the initial version of this work), we now have studied other oxidizers/reducing agents: H_2O_2 for oxidation and TCEP for reduction. Our experiments show that KKKC can be oxidized and reduced by these chemicals, leading to coacervate formation and destruction in an analogous way to our electrochemical observations. We now include these results in Figure S1 (Discussion in page 2)

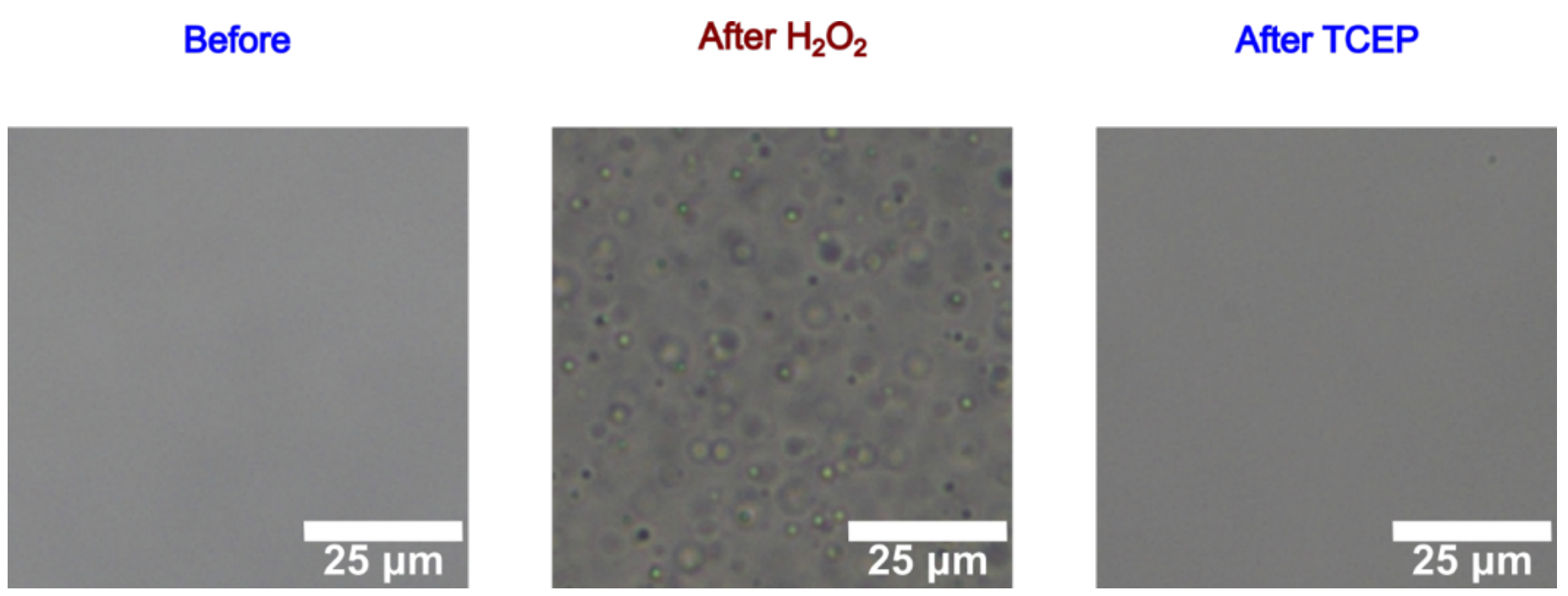

Figure S1. Micrographs of a sample containing KKKC and pAA in a 2.5:1 ratio – after mixing, immediately after the addition of H_2O_2 (29.4 mM, 8.8 eq.), and immediately after addition of TCEP (32.1 mM, 9.6 eq.). The concentrations of polyelectrolytes were [KKKC] = 3.34 mM, [pAA] = 4 mM (in monomer units). The samples contained Tris buffer (50 mM, pH = 7.4) and NaCl (100 mM). The scale bars are 25 µm.

3. Movement of droplets is interesting, and I'd be more curious about the role of sodium and chloride ions and also counter ions from the salt of polymer. Do they play a crucial role for movement?

The movement of coacervate droplets in an electric field was studied in detail in a recent publication by Spruijt (van Haren et al. *Nat. Commun.* **2024**, ref [42]). Their work shows that increasing the ion concentration normally increases the mobility of coacervate droplets. This is due to charge screening effects on the surface of the

coacervates: the mobility of the coacervates is inversely proportional to the Debye length (k^{-1}), which is in turn inversely proportional to the total ion concentration of the solution.

4. In figure 4, MV is supporting the reduction of peptide to resultantly destroy the droplets, after destruction is the reformation of coacervate time remain same or it is changed? can MV slow down the destruction through possibly cation-pi interactions?

We have compared the chemical oxidation and reduction of KKKC (with H_2O_2 and TCEP, respectively) in presence and in absence of MV (Figure S1 and S15). In both cases, the formation and destruction of the droplets was almost immediate, and the droplets were not visually different with and without MV. This indicates that the effect of MV in the internal structure of the coacervates must be minimal. This is consistent with our electrochemical experiments (Figure 5), in which the presence of MV does not significantly change the rate of formation of coacervates in the surface of the anode. The discussion on this topic has been added to Page 6.

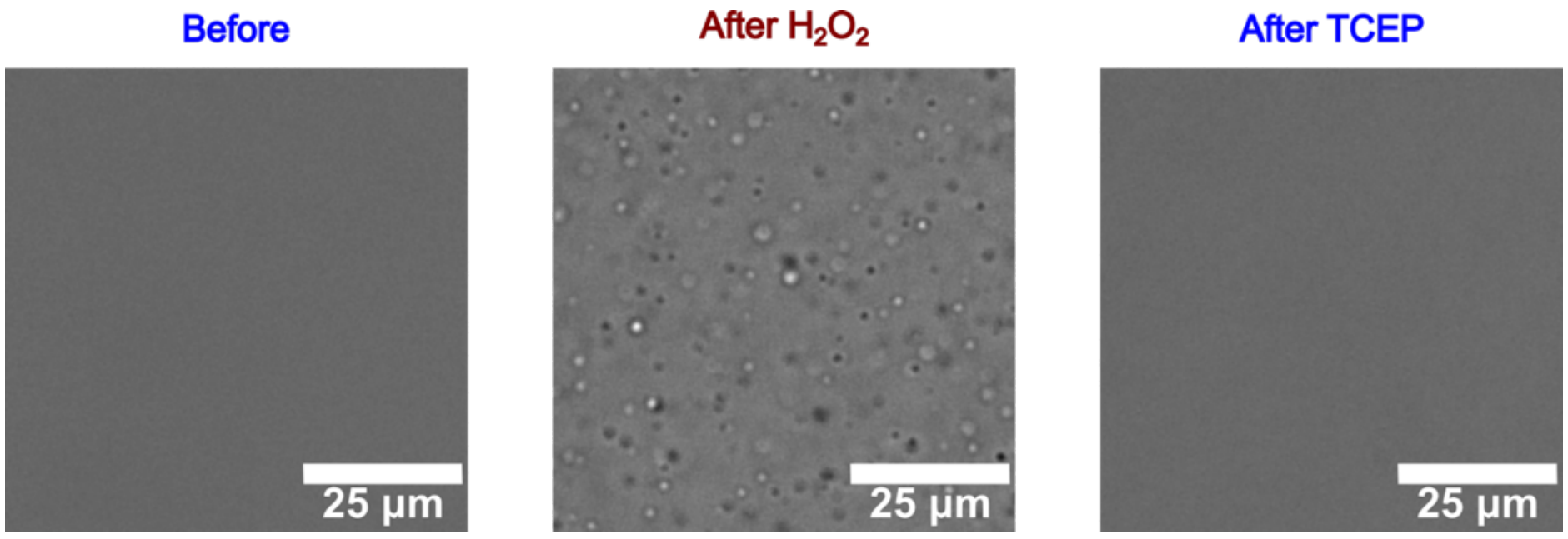

Figure S15. Micrographs of a sample containing KKKC and pAA in a 2.5:1 ratio and MV. The micrographs were recorded upon mixing; immediately after the addition of H_2O_2 (29.4 mM, 8.8 eq.); and immediately after the addition of TCEP (32.1 mM, 9.6 eq.). [KKKC] = 3.34 mM, [pAA] = 4 mM (in monomer units), [MV] = 6.68 mM. The samples contain Tris buffer (50 mM, pH = 7.4) and NaCl (100 mM). The scale bars are 25 µm. No significant differences can be observed between the coacervates formed in this case and those formed without MV (Figure S1).

5. In figure 4c, after 1 minute the blue color indicates the MV but droplets can be still seen in the image, would be great to detailed response why still coacervates remain unchanged.

The coacervates after 1 minute were significantly smaller than those at the beginning of the experiment (see panel d for a quantitative analysis of their relative sizes). We take this as an indication of their dissolution.

6.In Figure S13, different dyes have been used to encapsulate inside coacervate droplets, authors should show partition coefficient of each dye and how these dyes effect the movement and destruction/reformation of coacervates. Authors should also show the impact of these dyes on fluidity of coacervate droplets maybe FRAP could help to investigate this.

In order to probe the effect of dye compartmentalization in the formation and destruction of coacervates, and in combination with a suggestion from Reviewer 1, we performed an experiment in presence of Brooker's merocyanine. In this experiment, we applied an electric potential in absence of MV, observing the formation, movement, and accumulation of coacervates as described in Figure 5. MV was then added to this solution, observing the regeneration of coacervate droplets in the anode and the establishment of a steady state. (Figure S18). The presence of Brooker's merocyanine did not seem to impact these processes, and the dye remained compartmentalized at the end of the cycle.

In order to study the fluidity of the coacervates in presence of the different dyes, we recorded timelapses of different coacervate samples in their presence. For this experiment, we only selected the dyes that were compartmentalized inside of the coacervates – Brooker's Merocyanine and Nile Red for KKKC/pAA and Rhodamie B, Nile Red, Fluorescein and Brooker's Merocyanine for KKKC/ATP. In all of the cases, we could observe different droplets growing and merging in the bottom of the plate, indicating that they remained liquid upon dye compartmentalization (Figures S27 and S28)

Finally, we added TCEP to these dye-containing coacervates, observing their immediate reduction in all cases. This confirms that the compartmentalization of these dyes does not significantly change the dynamics of the coacervate phase, nor its redox-responsiveness (Figures S29 and S30).

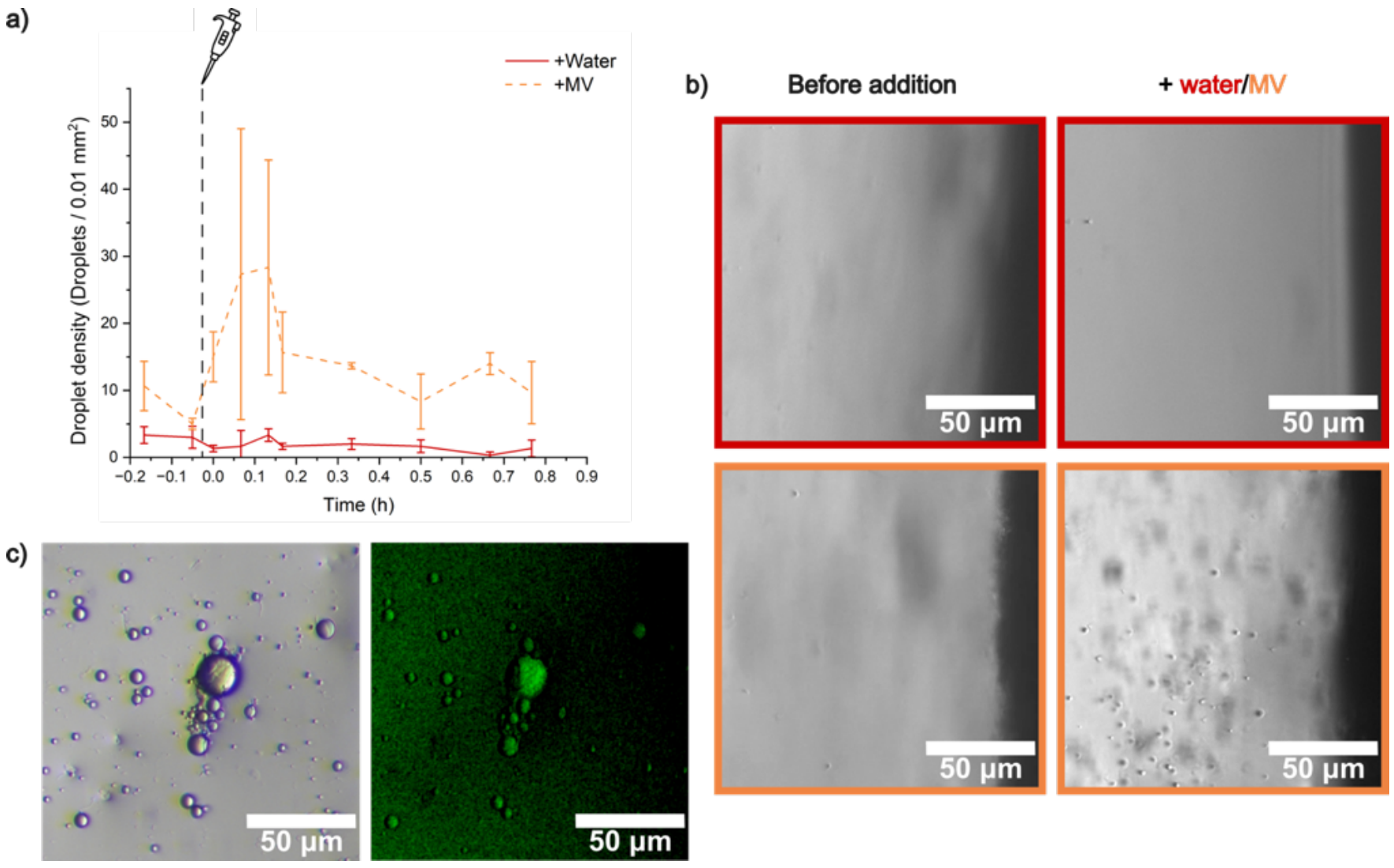

Figure S18. Regeneration of coacervates by addition of MV, after KKKC had been depleted from the anode. a) Average droplet density in the proximity of the anode before and after the addition of MV (orange dashed line). An identical sample, to which water was added instead of MV, is represented in red. The reported numbers correspond to the average of three 100 x 100 µm areas next to the surface of the electrode, and the error bars to their standard deviation. b) Micrographs of the area next to the anode in both samples, before and after addition of MV or water. c) Brightfield and fluorescence micrographs of coacervates in the bottom of the cell, showing the encapsulation of Brooker's merocyanine. [KKKC] = 3.34 mM, [pAA] = 4 mM, [MV] = 33.34 mM, [Brooker's merocyanine] = 0.95 µM. The scale bars are 50 µm.

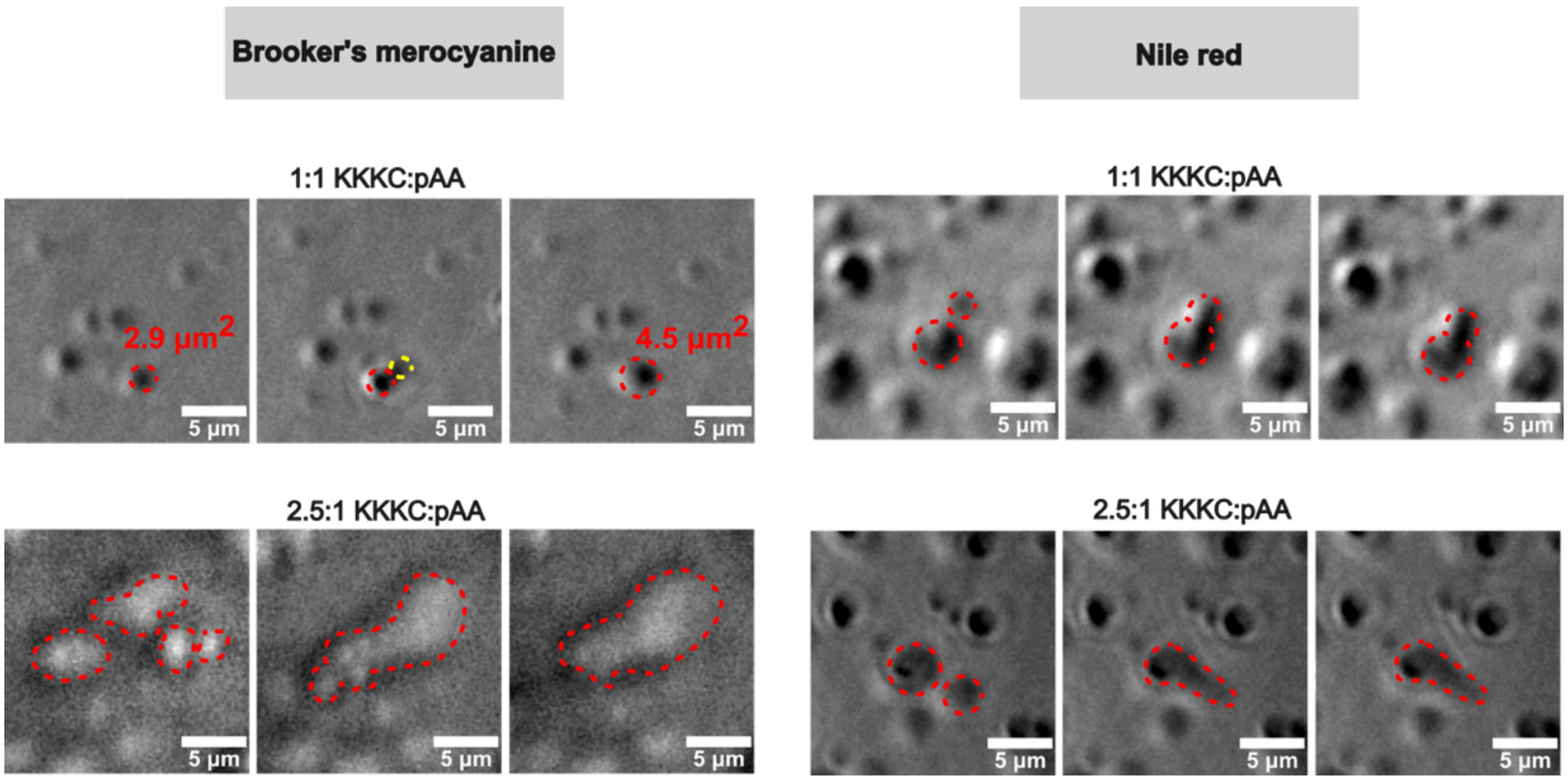

Figure S27. Merging of pAA-based coacervates containing Brooker's merocyanine and Nile Red dyes. In all cases, the three micrographs represent a series of frames separated by 2 minutes. The merging droplets have been outlined to aid visualization. The coacervates were prepared by mixing $(KKKC)_2$ with a solution containing pAA and the corresponding dye. [KKKC] = 1.34 mM or 3.34 mM, [pAA] = 4 mM in monomer units. The concentration of the dyes was 0.95 µM. The scale bars are 5 µm.

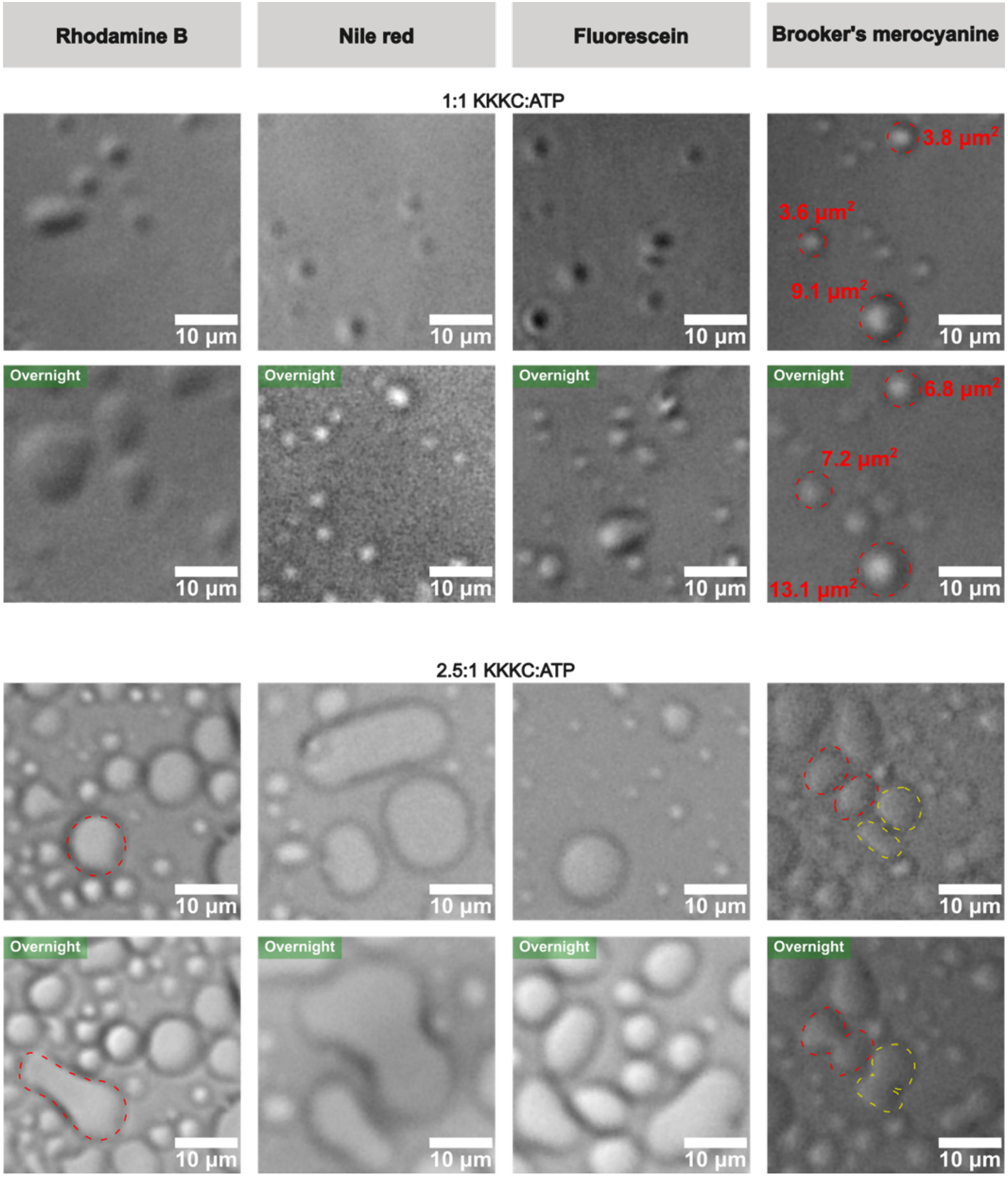

Figure S28. Merging of ATP-based coacervates containing Rhodamine B, Nile Red, Fluorescein and Brooker's merocyanine. The coacervates were obtained via electrochemical oxidation of 1:1 and 2.5:1 KKKC:ATP mixtures (0.7 V) and incubated with the dyes overnight. For all the samples the micrographs were recorded at exactly the same positions immediately after deposition and overnight. Some droplets have been outlined to aid with visualization. [KKKC] = 1.34 mM or 3.34 mM, [ATP] = 1.34 mM. The concentration of all dyes was 0.95 µM. The scale bars are 10 µm.

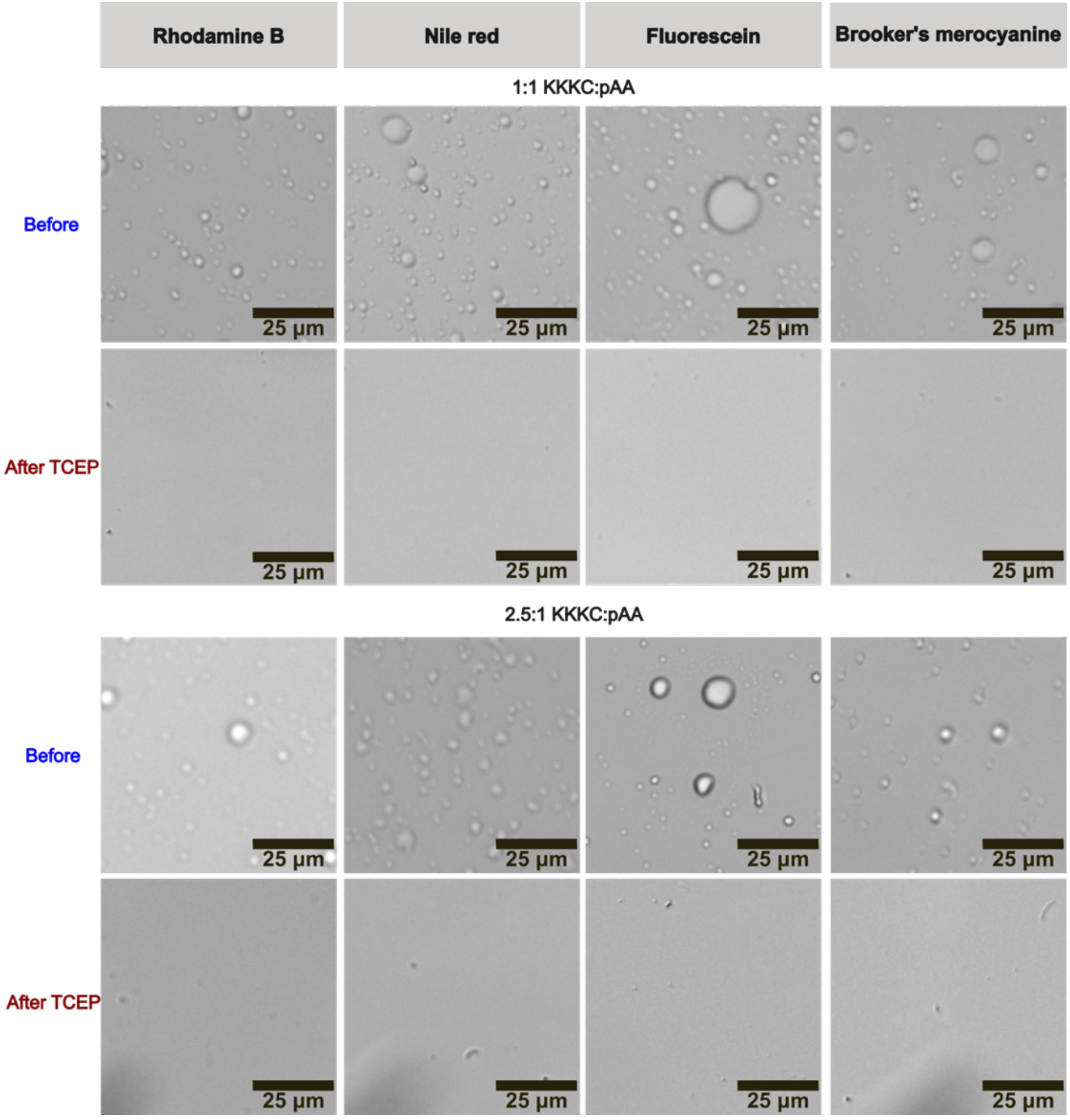

Figure S29. Dissolution of pAA-based coacervates in presence of different dyes upon addition of TCEP (19.6 mM). The coacervates were prepared via electrochemical oxidation of 1:1 and 2.5:1 KKKC:pAA mixtures (0.7 V) and incubated with Rhodamine B, Nile Red, Fluorescein or Brooker's merocyanine overnight. The micrographs before and after TCEP addition were recorded in the same positions of the well-plate. [KKKC] = 1.34 mM or 3.34 mM, [pAA] = 4 mM in monomer units. The concentration of all dyes was 0.95 µM. The scale bars are 25 µm.

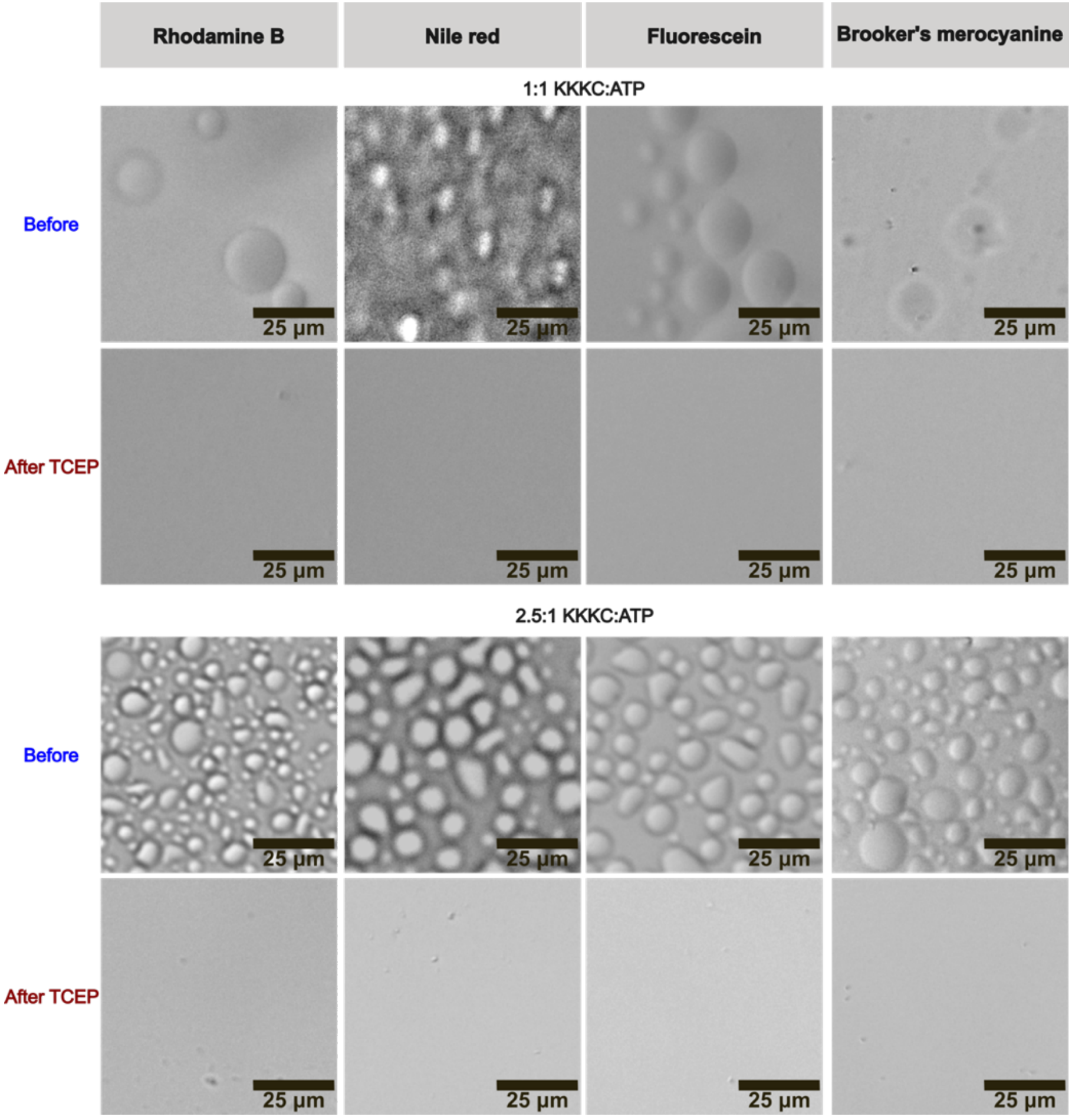

Figure S30. Dissolution of ATP-based coacervates in presence of different dyes upon addition of TCEP (19.6 mM). The coacervates were prepared via electrochemical oxidation of 1:1 and 2.5:1 KKKC:ATP mixtures (0.7 V) and incubated with Rhodamine B, Nile Red, Fluorescein and Brooker's merocyanine overnight. The micrographs before and after TCEP addition were recorded in the same positions of the well-plate. For 1:1 KKKC:ATP the concentrations of the polyelectrolytes were: [KKKC] = 3.34 mM, [ATP] = 3.34 mM and the concentration of all dyes was 1.8 µM. For 2.5:1 KKKC:ATP the concentrations of the polyelectrolytes were: [KKKC] = 3.34 mM, [ATP] = 1.34 mM and the concentration of all dyes was 0.95 µM. The scale bars are 25 µm.

We thank the reviewers for their comments and suggestions.

The changes in the main text and SI have been highlighted in yellow in the respective files. In addition, we have included the new figures in this document as a response to each comment.

Reviewer #1 (Remarks to the Author):

The study by Samokhvalova and coworkers is an ambitious one where they explored a system of coacervate that forms and gets destroyed by cyclic redox in response to electric potential.

After addressing several previous comments from reviewers, the authors have amended the article and the supporting information with additional studies and clarifications, which adds to the coherence and boldness of reported findings. It should also be noted that the language and nature of presentation and explanations of various findings are uncomplicated. However, some shortcoming are apparent. s The electrodes and their potential conundrum remain despite additional study and new findings. Since the silver is playing a role, replacing those with inert ones could have been a way to easily assess the potential impact on the formation of the coacervates.

We have tried to replace the silver electrodes in the coacervate formation experiments, substituting them for inert glassy carbon electrodes (Figure S6). Unfortunately, we could not observe the formation of any coacervates with these electrodes. This lines up with our previous CV studies, in which thiol oxidation only took place when silver was used as a working electrode (Figures S5 and S7).

The general consensus in the literature is that cysteines are difficult to oxidize on the surface of commercial solid electrodes and require a high overpotential (see references 46-49 in the main text), and most groups working on the electrochemistry of cysteine develop their own surface-modified electrodes (see https://doi.org/10.1016/j.snb.2010.02.051 or https://doi.org/10.1021/ac0703707). In our case, silver electrodes seem to be an exception to this rule (probably due to its high affinity for thiols).

We agree with the reviewer that it would be preferrable to find electrodes that are *both* inert and sufficiently reactive with cysteine. However, this is not at all trivial. Although interesting in the long-term, we believe that this optimization is not necessary as this stage, since the focus of this manuscript is the coacervates themselves and the out-of-equilibrium chemical reaction cycle.

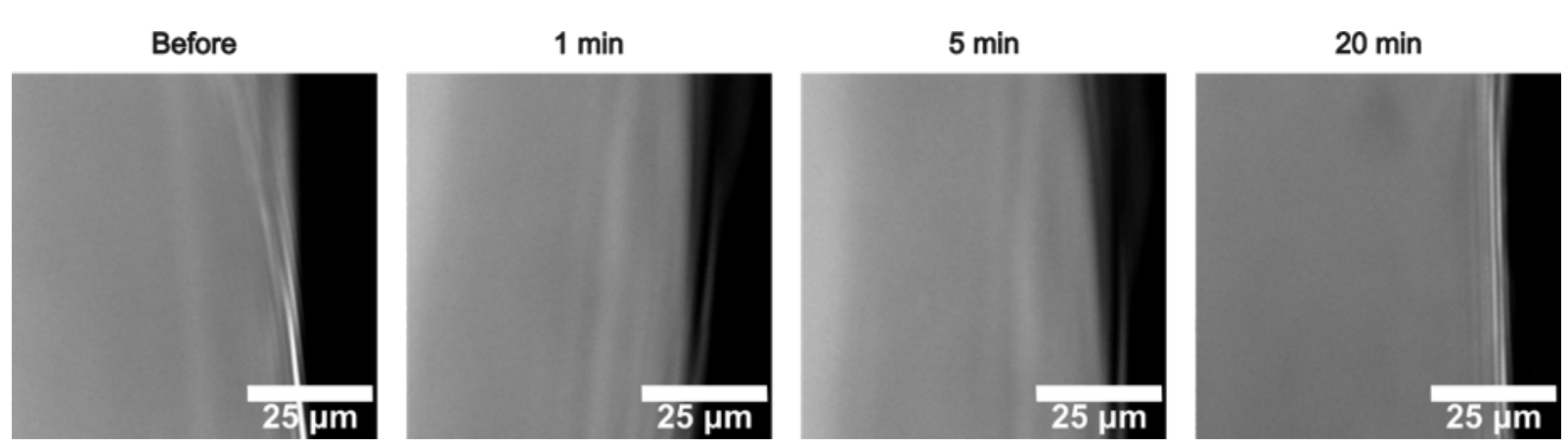

Figure S6. Micrographs of 2.5:1 KKKC:pAA mixtures in an electrochemical setup with glassy carbon flat electrodes. The micrographs were recorded next to the surface of the anode, before and during the application of an electric potential (0.7 V). [KKKC] = 3.34 mM, [pAA] = 4 mM (in monomer units). No coacervate droplets were detected throughout the experiment. The scale bars are 25 µm.

Although the claim is that it is waste-free, the authors do mention the degradation of the electrodes eventually causes the system to stop working. Rather than a qualitative assessment, a quantitative study would reveal if that were the case in actuality and also aid in revealing the potential role of silver ions.

As we discussed with another reviewer in the previous round, we use the term "Waste-free" in comparison to other chemically-fueled out-of-equilibrium systems. In this context, "waste" is commonly used to refer to the side-products *of the chemical reaction cycle itself*. Some examples of "waste accumulation" can be found in Donau et al. *Nat. Commun.* **2020** (Ref. [22] in the text, Figure S5 of their publication), Nakashima et al. *Soft Matter* **2018** (Ref. [27], Figure 4c of their publication), or Chen et al, *Chem. Sci.* **2023** (Ref. [34]), in which the authors discuss in depth this problem and some solutions).

While it is true that electrode degradation generates side-products, these side-products do not come from the reaction cycle itself: KKKC, pAA, and MV remain intact as proven by NMR, conductivity, and pH measurements (See Figures S24 and S26). Therefore, we believe that we are justified in using the term “waste-free” here, as it has been previously done to describe similar electricity-fueled systems (Barpuzary et al. *J. Am. Chem. Soc.* **2023**, ref. [37] in our text)

Even if our electrochemical “fueling” comes with its own set of challenges which end up disrupting the system, it is worth remarking the difference in timescales: our system stops working after > 16 h, two orders of magnitude longer than the lifetime of an individual droplet between formation and dissolution (10 minutes). For context, most out-of-equilibrium systems that suffer from waste accumulation stop working after ~5-10 cycles of formation-destruction (see examples above).

Regarding the role of silver ions in our system, we have obtained some new information about the mechanism in which they stabilize coacervates. By the suggestion of another reviewer, we have traced the phase diagram of our coacervates at different peptide:polyelectrolyte mixing ratios (Figure S10, see discussion below). As it can be seen in that Figure, the stabilizing effect of Ag^+ is stronger for coacervates that are close to charge-neutrality, or have a small excess of $(KKKC)_2$. This is consistent with our hypothesis that silver ions bind preferentially to the peptides and not to the polyanions, acting as transient crosslinks that stabilize the coacervate structure. However, these crosslinks are weak compared to electrostatic repulsions – since adding too much of either peptide or polyanion quickly dissolves the coacervates.

We plan to characterize the binding of silver to the coacervates in more detail in the near future, through a combination of quantitative experiments (e.g. ITC) and molecular dynamics simulations. However, we believe that this is enough work for a separate study, and that it is not necessary to back up the claims of this manuscript: the process that causes the coacervates to form and dissolve here is not silver, but the oxidation and reduction of KKKC

As the authors mention of competing redox reactions as a potential inhibitor to the continuity of the formation, movement and destruction, some simple steps to prevent such could have been taken, such as removal of oxygen to show if the presence of such contaminant is actually the reason behind it.

In order to study the effect of oxygen in our system, we have repeated the experiment from Figure 5 (steady state experiments), but degassing the solution beforehand, and sealing our experimental setup to prevent exchange with air. The results of this new experiment are shown in Figure S23 and Supplementary Video 5.

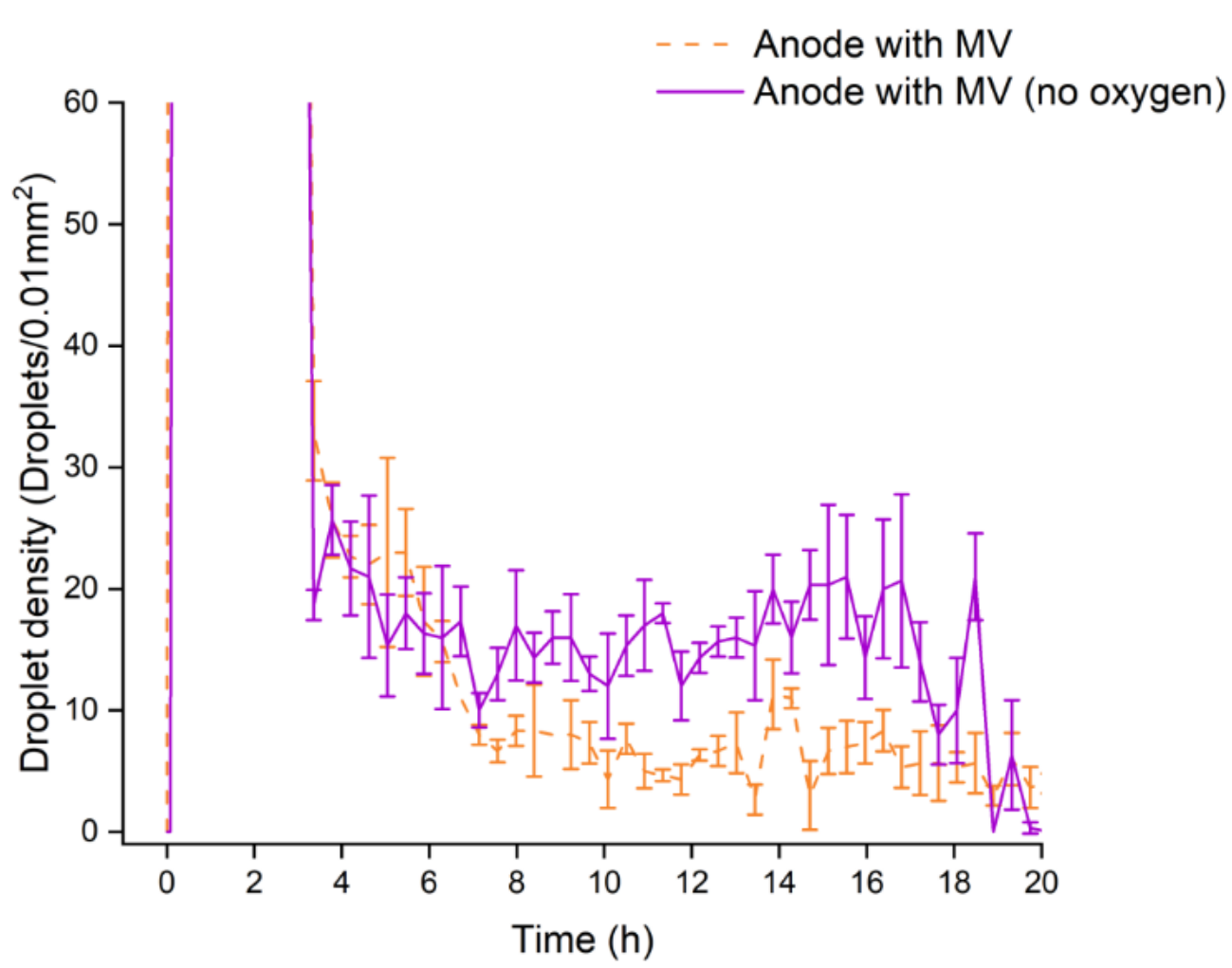

Figure S23. Effect of oxygen on the electrochemically-driven steady state. The line represents the average number of droplets per 0.01 mm^2, counted manually in three separate areas in the proximity of the anode. The error bars represent the standard deviation.

The result of two different experiments are reported here: one of them using degassed solutions and a sealed experimental setup (see Methods), and another one prepared without specific precautions to remove oxygen from the system. Both samples contained [KKKC] = 3.34 mM, [pAA] = 4 mM and [MV] = 6.68 mM. The potential applied was 0.7 V in both cases

The degassed sample reached a steady state similar to those described in Figure 5. In this case, the steady state contained a higher number of coacervates than for the non-degassed samples, confirming that oxygen acts as a contaminant that hampers the sustained formation of coacervates. There are two possible reasons for this: 1) The reduction of oxygen in the cathode competes with the reduction of MV, preventing the regeneration of thiols. 2) Oxygen accelerates the passivation of the silver electrodes, due to the formation of silver oxides.

Nonetheless, the steady state in the degassed sample was still lost after ~18 h, a time comparable to what we had observed in the experiments where O_2 was not removed. This could be further improved by optimizing the electrode chemistry to prevent their degradation, or by working in a completely closed cell to avoid water evaporation. However, as discussed above, we believe that these optimizations are not critical at this stage: the steady state decays after many cycles of the chemical reaction, showing a significant improvement over the state-of-the-art of chemically-fueled systems.

Although qualitative assessment of MV is reported, quantitative assessment would strengthen the claim of MV being the electrocatalytic mediator. Correlating total charge passed with the amount of disulfide formed would allow estimation of Faradaic efficiency and clarify whether competing side reactions significantly contribute.

We have studied the Faradaic efficiency of KKKC oxidation in presence and in absence of MV. The results are summarized in Figure S22

The Faradaic efficiency was calculated from two factors: the total charge passed through the system and the progress of the electrochemical reaction KKKC + e^- -> ½ $(KKKC)_2$ (which we derive from the concentration of thiols). However, it is important to note that in the samples containing MV, the disulfides that form in the anode can react with the $MV^{\cdot+}$ produced in the cathode, converting back to thiols and reducing the apparent conversion. Therefore, the apparent Faradaic efficiency reported here is an underestimation, lower than the real one.

Keeping this in mind, we interpret these results in the following way:

In absence of MV, the Faradaic efficiency is very low (~10-20%), mostly due to a low conversion of thiols. Adding MV provides a new counter-reaction to thiol oxidation, and results in a significant increase in apparent Faradaic efficiency (which goes up to 30-40% over the first two minutes of the experiment). After longer periods of time, the $MV^{\cdot+}$ formed at the cathode diffuses throughout the entire solution, reducing the disulfides even further, and the apparent Faradaic efficiency decreases.

This experiment confirms a double role of MV, which promotes the initial oxidation of thiols, but also their regeneration next to the anode, by acting as an electrocatalyst for the reduction of disulfides.

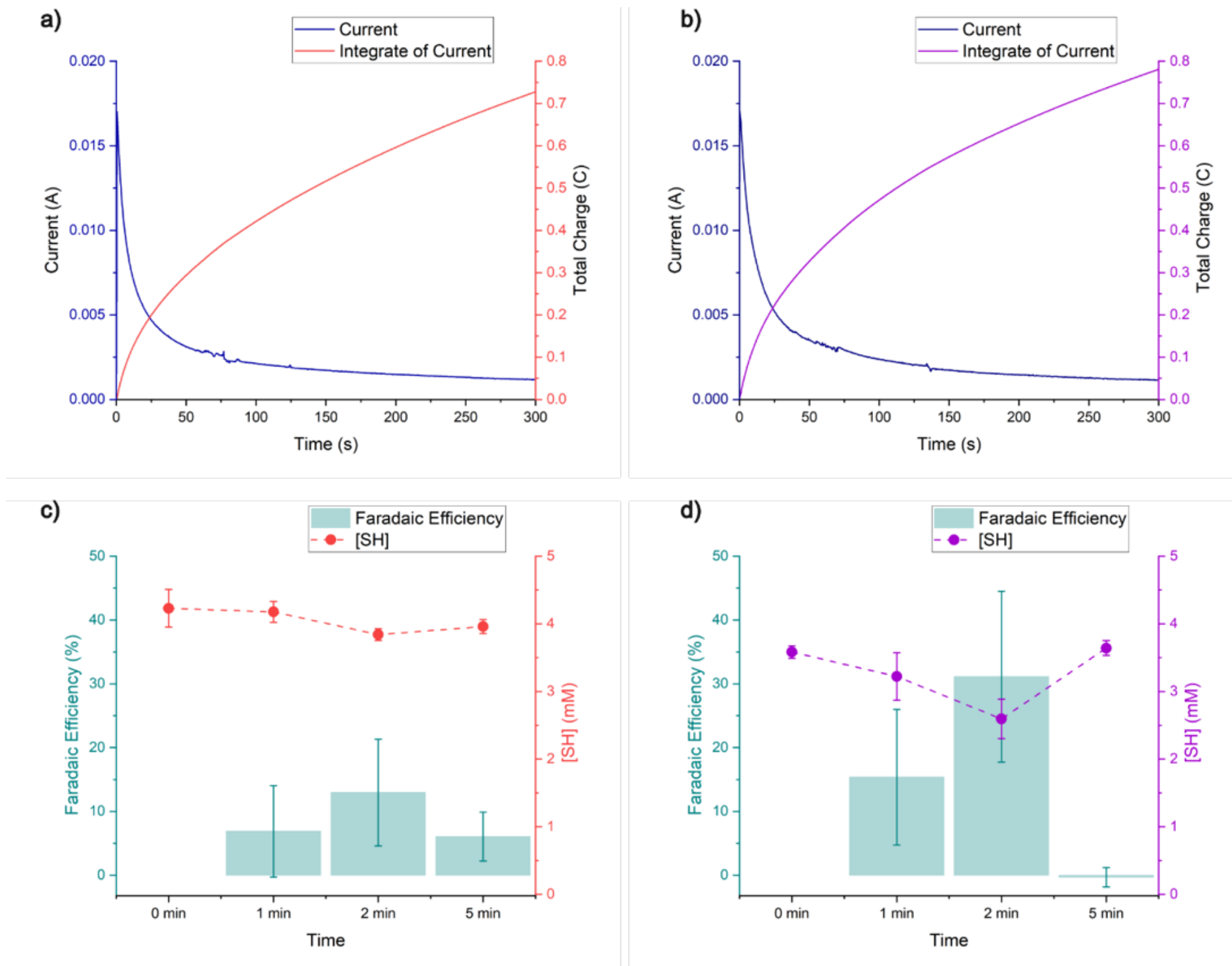

Figure S22. Chronoamperometric curve and total charge passed through the 2.5:1 KKKC:pAA system at 0.7 V a) in absence and b) in presence of MV. Faradaic efficiency of the 2.5:1 KKKC:pAA system and concentration of thiol groups of KKKC c) in absence and d) in presence of MV. The error bars represent the standard deviation between triplicated experiments. [KKKC] = 3.34 mM, [pAA] = 4 mM, [MV] = 6.68 mM. Note that both the conversion and Faradaic efficiencies represented in panel d) correspond only to "observed" values, since KKKC is simultaneously being consumed by oxidation and produced by reaction with $MV^{\cdot+}$. Therefore, the real conversion and Faradaic efficiency are higher than the values shown in this panel.

As an additional experiment to quantify the reaction between MV and $(KKKC)_2$, we prepared six coacervate samples by chemical oxidation of KKKC in presence of pAA. We then applied an electric potential as usual and added MV to three of these samples, and water to the other three. We monitored the concentration of thiols in all of the cases by UV-Vis spectroscopy, using a quantitative Ellman's assay (see Methods). As we show in Figure S21, the concentration of thiols doubled in the sample to which MV was added, and remained constant in the control – further confirming that MV mediates the regeneration of KKKC.

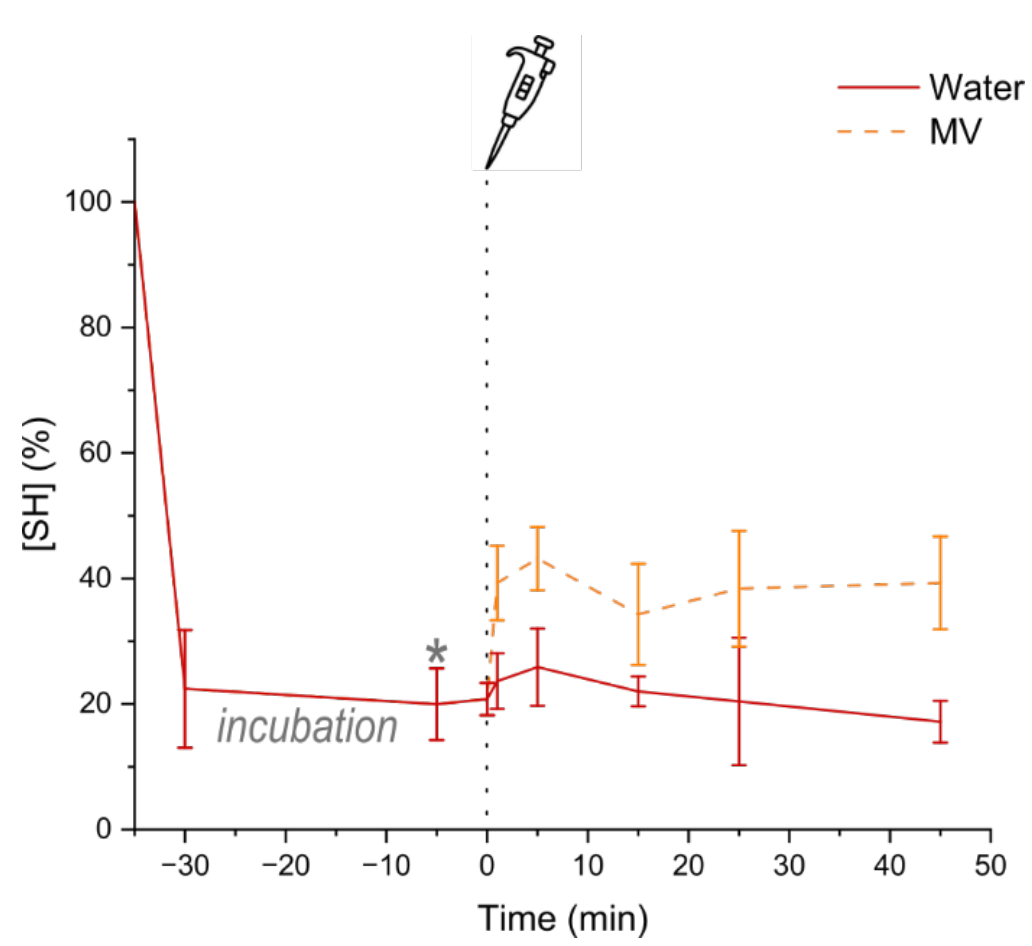

Figure S21. Regeneration of KKKC by the reaction between MV and $(KKKC)_2$ in an oxidized system. When MV was added to a coacervate sample and an electric potential was applied, the concentration of thiols (quantified by UV-Vis spectroscopy using the Ellman's assay) doubled. However, when an identical volume of water was added to the system, no increase in thiol concentration was observed. The asterisk marks the starting time of application of an electric potential (0.7 V), and the dashed line represents the time in which either water or MV were added. The error bars represent the standard deviation between triplicated experiments. [KKKC] = 3.34 mM, [pAA] = 4 mM, [MV] = 33.34 mM.

Reviewer #2 (Remarks to the Author):

I co-reviewed this manuscript with one of the reviewers who provided the listed reports. This is part of the Nature Communications initiative to facilitate training in peer review and to provide appropriate recognition for Early Career Researchers who co-review manuscripts.

Reviewer #3 (Remarks to the Author):

The revision has addressed most of my previous concerns; however, one point remains as indicated in Comment 2. How did the authors determine the charge mixing ratios of 1/1 and 2.5 as the representative cases? Are there any results to support that these ratios are ideal stoichiometric and non-stoichiometric mixing charge ratios? This selection appears somewhat arbitrary. A comprehensive screening of turbidity under a broader range of charge ratios could clarify the rationale for choosing these specific ratios for investigation.

Previous studies (references 42-44 in the main text) have shown that the electrophoretic mobility of complex coacervates is correlated to the ratio between positive and negative charges. We initially selected the 1:1 and 2.5:1 ratios as representative examples of coacervates that would be neutral and positively charged, and therefore would stay stationary or move towards the cathode. This was confirmed in the experiments shown in Figure 3.

Nonetheless, we have now characterized the formation of coacervates across a broader range of mixing ratios, measuring their stability against salt (Figure S10). Interestingly, the 1:1 and 2.5:1 coacervates were among the most stable ones in all cases.

Additionally, the phase diagrams resulting from these experiments provide new information about the interactions between $(KKKC)_2$ coacervates and silver ions. For samples without Ag^+, the mixing ratio does not have a strong influence on the stability of the coacervates. Adding Ag^+ stabilizes the coacervates, and this stabilization is stronger for coacervates that are either neutral or slightly positively charged. This is consistent with our initial hypothesis: Ag^+ binds to $(KKKC)_2$, providing transient crosslinks that stabilize the coacervate structure. However, these crosslinks are weak compared to electrostatic repulsions, since adding a large excess of either positive or negative charges quickly results in coacervate dissolution.

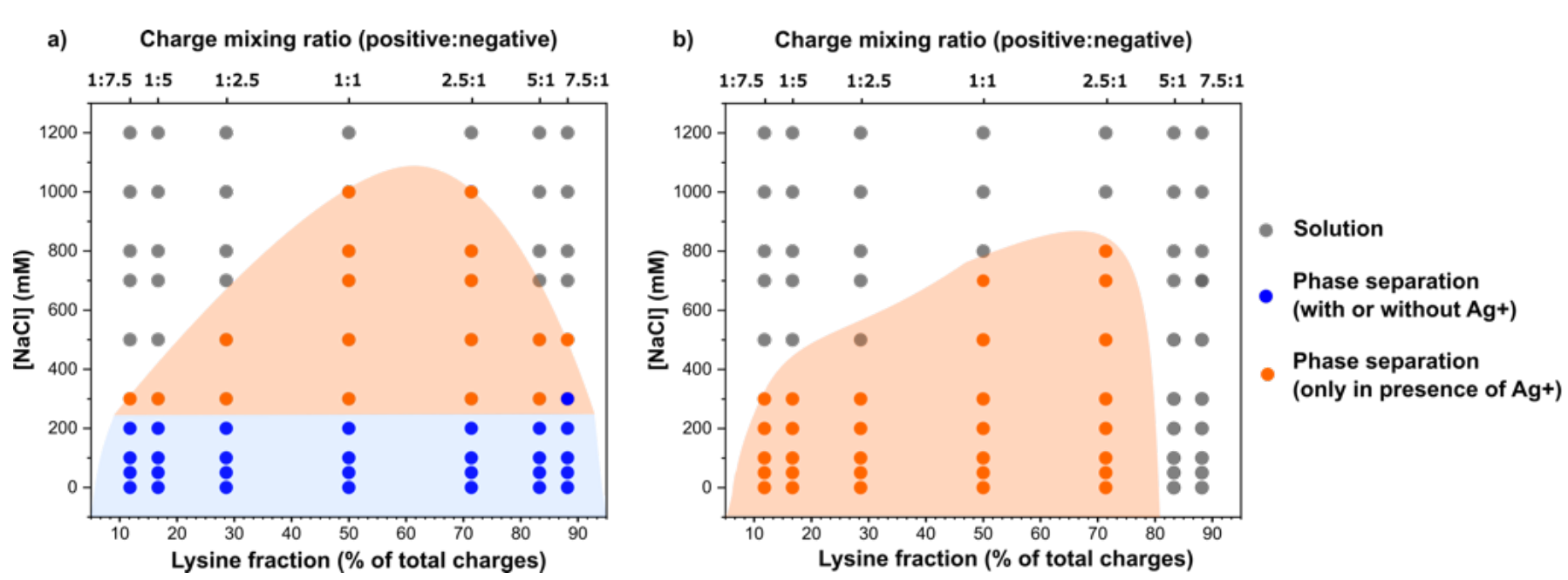

Figure S10. Phase diagrams of $(KKKC)_2$/pAA and $(KKKC)_2$/ATP coacervates prepared with various charge ratios for the systems a) $(KKKC)_2$/pAA, and b) $(KKKC)_2$/ATP. The samples in presence of Ag^+ contained 0.01 equivalents of $Ag(NO)_3$ with respect to KKKC.

Reviewer #4 (Remarks to the Author):

The authors have adequately addressed all of my concerns. I have no further questions and recommend this manuscript for publication in Nature Communications.

Reviewer #1 (Remarks to the Author):

The authors have done a commendable job in addressing concerns regarding the replacement of electrodes and the influence of oxygen in the system. However, the role of silver in the coacervation process still requires further clarification. Since silver appears to participate in or influence the complexation process, a more detailed discussion regarding its exact role would strengthen the manuscript. In particular, clarification regarding possible Ag–thiol coordination effects and Ag^+-induced stabilization of the coacervate phase would be valuable.
Regarding the use of the term “waste-free”, I partially agree with the authors’ justification, especially considering similar terminology has been used in related previous studies. Nevertheless, some refinement in the terminology or additional clarification could help avoid ambiguity for readers from broader electrochemical or materials backgrounds.
The Faradaic efficiency experiments provide useful insight into the role of methyl viologen (MV), and the observed trends support its involvement in the electrochemical cycle. However, the relatively low Faradaic efficiencies also raise questions regarding the fate of the remaining electrons and the possible contribution of competing electrochemical reactions- which could be addressed in subsequent studies.
Overall, revisions have considerably improved both the scientific clarity and the presentation of the work.

We thank the reviewers for their comments. We have now expanded the discussion on these two topics in the main text:

- Regarding the “waste-free” terminology, we have expanded the corresponding section of the introduction (page 2), explicitly defining what we mean by “waste” and why these systems have been described as “waste-free” before.
- We have also added a paragraph in the discussion (page 9) summarizing our previous exchanges with the reviewer: why the side-reactions happening in the electrode are fundamentally different than waste accumulation in other out-of-equilibrium systems, and why this distinction is important

- Regarding the role of silver ions on the stability of the coacervate phase, we now describe in detail what is our current hypothesis (Page 3): Ag^+ binds to the disulfide groups of $(KKKC)_2$, linking multiple peptides together. This creates a new polycation with increased multivalency, favoring coacervate formation and stabilizing the coacervate phase. We also include a new reference ([54] D. Carrillo, “Organodisulphide complexes” *Coordination Chemistry Reviews* **1992**, *119*, 137–169.), which describes the formation of a coordination complex between Ag^+ and disulfides. This complex links together 4 disulfides and two silver nuclei (see below). If such a complex (or a similar one) was formed between $KKKC_2$ and Ag^+, as we hypothesize, it would be highly charged, explaining the observed stabilization of the coacervate phase.
- Finally, we also clarify in page 3 that the majority of experiments of the paper were performed with the KKKC:pAA system and a salt concentration of 100 mM. As shown in Figure 10, in these conditions coacervates form upon oxidation with or without silver – therefore, the effect of silver ions in our results should be minimal.

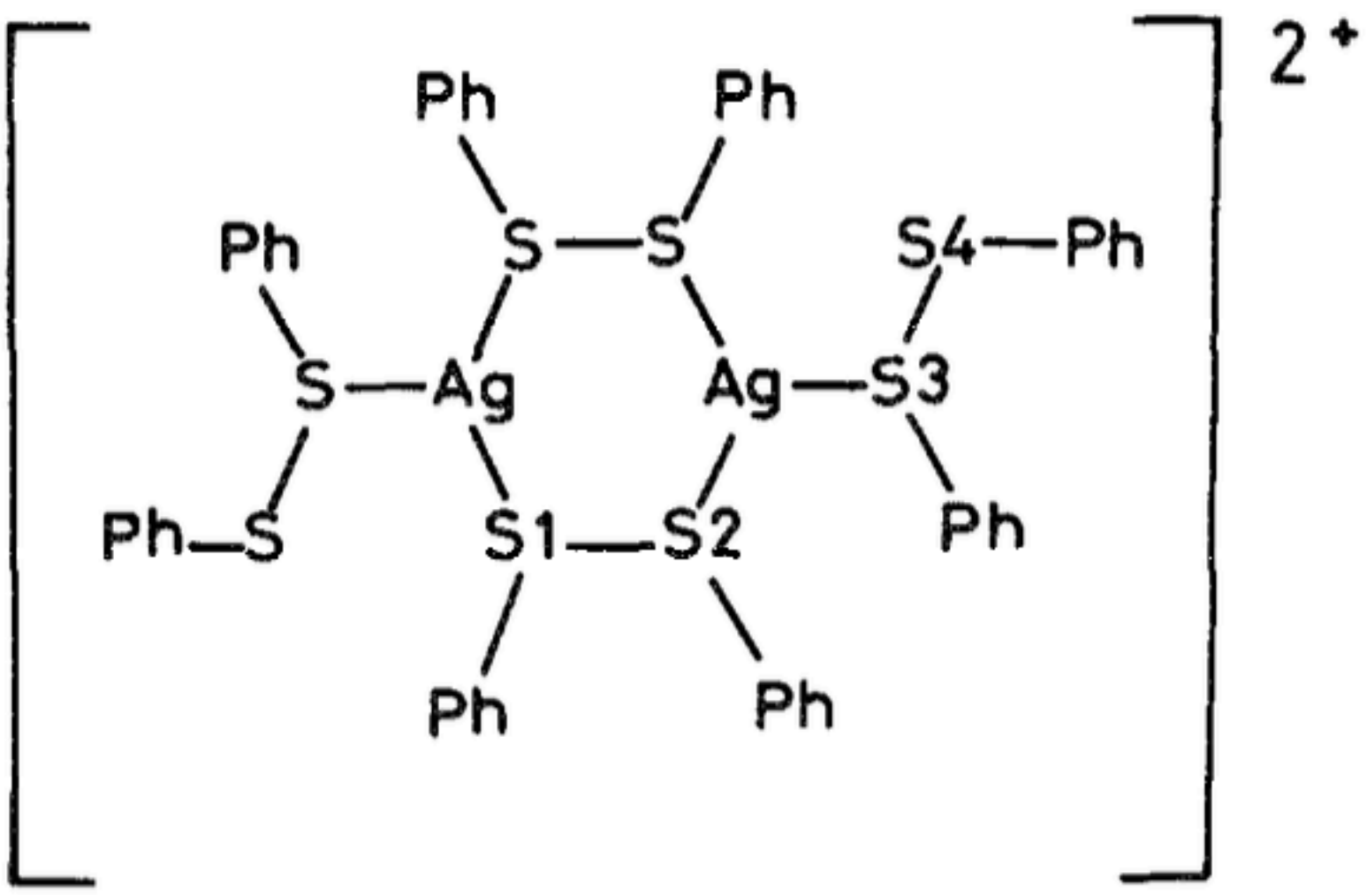

III

Reviewer #2 (Remarks to the Author):

I co-reviewed this manuscript with one of the reviewers who provided the listed reports. This is part of the Nature Communications initiative to facilitate training in peer review and to provide appropriate recognition for Early Career Researchers who co-review manuscripts.

The authors have addressed all my concerns and comments, and I have no further questions/comments. I support the publication of this manuscript in Nature Communications.
